# Supplementary material for: The overlap of genetic susceptibility to schizophrenia and cardiometabolic disease can be used to identify metabolically different groups of individuals
Source: Sci Rep. 2021 Jan 12;11:632. doi: 10.1038/s41598-020-79964-x (PMC7804422; doi:10.1038/s41598-020-79964-x)

The overlap of genetic susceptibility to schizophrenia and cardiometabolic disease can be used to identify metabolically different groups of individuals

Rona J. Strawbridge[1,2,3*], Keira J. A. Johnston [1,4,5], Mark E. S. Bailey [5], Damiano Baldasarre[6,7], Breda Cullen [1], Per Eriksson[3], Ulf deFaire[8], Amy Ferguson[1,9], Bruna Gigante[8], Philippe Giral[10], Nicholas Graham[1], Anders Hamsten[3], Steve E. Humphries[11], Sudhir Kurl[12], Donald M. Lyall[1], Laura M. Lyall[1], Jill P. Pell[1], Matteo Pirro[13], Kai Savonen[14,15], Andries J. Smit[16], Elena Tremoli[7], Tomi-Pekka Tomainen[17], Fabrizio Veglia[7], Joey Ward[1], Bengt Sennblad[18] and Daniel J. Smith[1]

[1]Institute of Health and Wellbeing, University of Glasgow, Glasgow, UK
[2]Health Data Research, UK
[3]Department of Medicine Solna, Karolinska Institute, Stockholm, Sweden.
[4]Deanery of Molecular, Genetic and Population Health Sciences, College of Medicine and Veterinary Medicine, University of Edinburgh, Scotland, UK
[5]School of Life Sciences, College of Medical, Veterinary & Life Sciences, University of Glasgow, Scotland, UK
[6]Department of Medical Biotechnology and Translational Medicine, Università degli Studi di Milano, Milan, Italy
[7]Centro Cardiologico Monzino, IRCCS, Milan, Italy
[8]Cardiovascular Medicine Unit, Department of Medicine Solna, Karolinska Institutet, Stockholm, Sweden.
[9]Usher Institute, University of Edinburgh, UK
[10]Assistance Publique - Hopitaux de Paris; Service Endocrinologie-Metabolisme, Groupe Hôpitalier Pitie-Salpetriere, Unités de Prévention Cardiovasculaire, Paris, France.
[11]Centre for Cardiovascular Genetics, Institute Cardiovascular Science, University College London, London, UK
[12] Institute of Public Health and Clinical Nutrition, University of Eastern Finland, Kuopio, Finland
[13]Internal Medicine, Angiology and Arteriosclerosis Diseases, Department of Clinical and Experimental Medicine, University of Perugia, Perugia, Italy.
[14]Foundation for Research in Health Exercise and Nutrition, Kuopio Research Institute of Exercise Medicine, Kuopio, Finland.
[15]Department of Clinical Physiology and Nuclear Medicine, Kuopio University Hospital, Kuopio, Finland.
[16]Department of Medicine, University Medical Center Groningen and University of Groningen, Groningen, The Netherlands
[17]Public Health and Clinical Nutrition, Department of Medicine, University of Eastern Finland, Kupiou, Finland
[18]Department of Cell and Molecular Biology, National Bioinformatics Infrastructure Sweden, Science for Life Laboratory, Uppsala University, Uppsala, Sweden

Supplemental data

- Full list of contributors to the IMPROVE study
- Supplemental Methods
- Supplemental Tables
- Supplemental Figures

Supplemental members of the IMPROVE study group

- Centro Cardiologico Monzino, IRCCS, Milan Italy: E. Tremoli, D. Baldassarre, M. Amato, B. Frigerio, A. Ravani, D. Sansaro, D. Coggi, F. Veglia, C. Tedesco, N. Capra, A. Bonomi.

- Department of Medical Biotechnology and Translational Medicine, Università di Milano, Milan, Italy: D. Baldassarre.

- Dipartimento di Scienze Farmacologiche e Biomolecolari, Università di Milano, Milan, Italy: Laura Calabresi, C.R. Sirtori.

- Cardiovascular Medicine Unit, Department of Medicine Solna, Karolinska Institutet. Per Eriksson, Angela Silveira, Bruna Gigante.

- Division of Cardiovascular and Nutritional Epidemiology, Institute of Environmental Medicine, Karolinska Institutet. Karin Leander, Federica Laguzzi.

- Cardiovascular Medicine Unit, Department of Medicine Solna, Karolinska Institutet. Anders Hamsten.

- Division of Cardiovascular and Nutritional Epidemiology, Institute of Environmental Medicine, Karolinska Institutet. Ulf de Faire.

- Cardiovascular Genetics, Institute Cardiovascular Science, University College of London, Rayne Building, University Street, London, United Kingdom. Steve E. Humphries, J. Cooper, J. Acharya.

- Foundation for Research in Health Exercise and Nutrition, Kuopio Research Institute of Exercise Medicine, Kuopio, Finland: K. Savonen, K. Huttunen, E. Rauramaa, I.M. Penttila, J. Törrönen.

- Department of Medicine, University Medical Center Groningen, Groningen & Isala Clinics Zwolle, Department of Medicine; the Netherlands: Andries J. Smit, A.I. van Gessel, A.M van Roon, A. Nicolai, D.J. Mulder, G.H. Smeets.

- Sorbonne Université, INSERM UMR1166, cardiovascular prevention unit, AP-HP, Groupe Hôpitalier Pitié-Salpetriere, Paris, France: Philippe Giral, Anatole Kontush, Alain Carrié, Antonio Gallo.

- Institute of Public Health and Clinical Nutrition, University of Eastern Finland, Kuopio Campus: Sudhir Kurl, J. Karppi, T. Nurmi, K. Nyyssönen, T.P. Tuomainen, J.Tuomainen, J. Kauhanen.

- Internal Medicine, Angiology and Arteriosclerosis Diseases, Department of Medicine, University of Perugia, Perugia, Italy: M. Pirro, M.R. Mannarino, G. Vaudo, V. Bianconi, D. Siepi, G. Lupattelli.

Supplemental Methods

Validation of clustering:

To test the generality of our results with respect to clustering method, we additionally analysed our IMPROVE data using PCA and t-SNE. Both these methods are based on identifying principal components, in contrast to MDS, which is based on a pairwise distance analysis. While the principal components the end result for PCA, t-SNE uses them in a neighbourhood analysis and then (iteratively) estimates a lower dimension mapping of the samples that optimately retain this neighborhood.

tSNE analysis

A tSNE ananlysis was performed using the R package Rtsne . Default settings the number of iterations (max_iter=1000), while forthe balance between speed and accuracy, we used a valuetheta=0.1, that is, tending more towards accuracy.. As the values for perplexity and number of PCA dimensions used in the input can influence the results, a number of different settings were compared: For perplexity (which can be thought of as controlling the number of neighbours considered), three values were chosen (25, 50 and 100). For the number of principle components to use as input (initial_dims), three values were tested (2, 5 and 10).

PCA analysis:

PCA was also performed using the R package prcomp.

Supplemental Tables

- Supplementary Table 1: IMPROVE clusters based on different MAF filters
- Supplementary Table 2: Demographic characteristics of the UKB2 MH and no MH participants, by cluster
- Supplementary Table 3: Genetic loci associated with SCZ, MDD, BD and present on the illumine CardioMetabo and Immuno chips.
- Supplementary Table 4: Genetic loci associated with eye colour.

Supplemental Figures

- Supplemental Figure 1: Results of MDS analysis in IMPROVE, using the loci in common between CMD and SCZ with MAF >1%, where latitude is a) 43 ˚N, b) 45 ˚N, c) 48 ˚N, d) 53 ˚N, e) 59 ˚N, f) 62 ˚N.
- Supplementary Figure 2: Results of tSNE analysis with dimensions set to 2 and perplexity to A) 25, B) 50 and C) 100, or dimensions set to 5 and perplexity to D) 25, E) 50 and F) 100, or dimensions set to 5, perplexity to G) 25, H) 50 and I) 100. Individuals (each dot) is coloured/numbered according to the group assignment from MDS analysis.
- Supplementary Figure 3: Results of PCA analysis. Individuals (each dot) is coloured/numbered according to the group assignment from MDS analysis.
- Supplementary Figure 4: Results of MDS analysis in UKB2 using all SCZ-associated loci.
- Supplementary Figure 5: Results of MDS analysis in UKB2 using eye colour-associated loci.

STable1: IMPROVE clusters based on different MAF filters

		MAF >10%		
		1	2	3
	1	1160	0	0
	2	0	1375	1
MAF >1%	3	0	0	519

STable 2: Demographic characteristics of the UKB2 MH and no MH participants, by cluster

		UKB2 no MH					UKB2 MH				
		1	2	3	.	P	1	2	3	.	
	N (%)	2970 (17.5)	7761 (45.8)	4966 (29.3)	1254 (7.4)		1198 (37.1)	1491 (46.2)	499 (15.4)	42 (1.3)	
	Male (%)	1507 (50.7)	3932 (50.7)	2468 (49.7)	665 (47.0)	0.515	460 (38.4)	544 (36.5)	168 (33.7)	28 (66.7)	0.175
baseline	age	55.5 (7.5)	55.4 (7.5)	55.3 (.74)	55.4 (7.5)	0.241	54.1 (7.4)	54.5 (7.4)	53.5 (7.2)	52.5 (6.9)	**0.037**
	weight	76.9 (14.4)	77.1 (14.6)	76.7 (14.8)	77.8 (14.8)	0.189	77.0 (15.8)	76.7 (15.8)	76.4 (14.8)	77.5 (16.4)	0.783
	waist	88 (12)	88 (12)	88 (13)	89 (13)	0.209	88 (14)	88 (14)	88 (13)	89 (16)	0.820
	hip	102 (8)	102 (8)	102 (8)	102 (8)	0.491	103 (9)	103 (9)	103 (9)	103 (10)	0.926
	whr	0.86 (0.09)	0.86 (0.09)	0.86 (0.09)	0.87 (0.09)	0.178	0.85 (0.09)	0.85 (0.09)	0.85 (0.09)	0.86 (0.11)	0.493
	bmi	26.5 (4.0)	26.5 (4.1)	26.5 (4.2)	26.7 (4.1)	0.277	27.0 (4.6)	27.1 (4.7)	27.1 (4.6)	27.4 (4.8)	0.947
	sbp	136 (17)	137 (18)	135 (18)	137 (18)	**<0.001**	133 (18)	134 (17)	131 (17)	134 (14)	**0.002**
	dbp	82 (10)	82 (10)	81 (10)	82 (10)	**0.012**	81 (10)	81 (10)	80 (9)	82 (9)	**0.023**
	sbp_adj	138 (19)	139 (20)	137 (19)	139 (20)	**0.001**	135 (19)	136 (19)	133 (19)	137 (15)	**<0.001**
	dbp_adj	83 (11)	83 (11)	83 (11)	84 (11)	**0.035**	83 (11)	83 (11)	81 (10)	83 (10)	**0.005**
	t2d	53 (1.8)	164 (2.1)	124 (2.5)	28 (2.2)	0.096	31 (2.6)	35 (2.4)	10 (2.0)	0 (0)	0.613
	htn	1296 (45.2)	3539 (47.6)	2079 (43.9)	576 (45.9)	**<0.001**	471 (40.5)	617 (42.6)	168 (34.5)	19 (45.2)	**0.007**
	htn_meds	430 (14.5)	1065 (13.8)	663 (13.4)	193 (15.4)	0.376	164 (13.7)	239 (16.2)	59 (11.9)	7 (16.7)	**0.037**
	lipid-lowering	241 (17.6)	642 (17.9)	445 (19.7)	124 (9.9)	0.141	91 (21.4)	101 (21.2)	29 (19.5)	3 (7.1)	0.875
	ish	42 (1.4)	105 (1.4)	68 (1.4)	13 (1.0)	0.970	9 (0.8)	17 (1.1)	5 (1.0)	0 (0)	0.590
	current smoking	153 (5.2)	471 (6.1)	291 (5.9)	73 (5.8)	0.132	87 (7.3)	121 (8.1)	35 (7.0)	3 (7.1)	0.677
	Ever smoker	1091 (36.7)	3004 (38.7)	1892 (38.1)	519 (41.4)	0.176	518 (43.2)	635 (42.6)	209 (41.9)	22 (52.4)	0.846
followup	age	63.6 (7.5)	63.4 (7.5)	63.4 (7.4)	63.4 (7.5)	0.340	62.1 (7.4)	61.5 (7.3)	61.5 (7.3)	60.8 (6.6)	**0.039**
	weight	76.1 (14.6)	76.3 (14.8)	75.9 (14.8)	77.0 (15.0)	0.162	76.9 (16.0)	76.4 (16.0)	7.77 (15.6)	79.5 (16.6)	0.596
	waist	88 (12)	88 (12)	88 (12)	89 (12)	0.280	89 (13)	89 (13)	89 (13)	92 (14)	0.641
	hip	101 (8)	101 (8)	101 (9)	101 (9)	0.581	102 (10)	103 (10)	103 (10)	105 (9)	0.861
	whr	0.87 (0.09)	087 (0.09)	0.87 (0.08)	0.88 (0.08)	0.540	0.87 (0.09)	0.86 (0.09)	0.86 (0.09)	0.87 (0.11)	0.382
	bmi	26.4 (4.1)	26.4 (4.3)	26.4 (4.3)	26.7 (4.3)	0.390	27.1 (4.7)	27.2 (4.9)	27.3 (5.0)	28.3 (4.9)	0.811
	sbp	138 (18)	138 (18)	138 (18)	138 (17)	0.488	135 (18)	136 (18)	133 (17)	137 (21)	0.052
	dbp	78 (10)	79 (10)	78 (10)	79 (10)	0.124	78 (11)	81 (11)	79 (11)	80 (10)	0.281

sbp_adj	141 (20)	141 (20)	141 (20)	142 (20)	0.488	138 (20)	139 (20)	136 (20)	141 (22)	**0.023**
dbp_adj	81 (11)	81 (11)	81 (11)	81 (11)	0.211	81 (12)	81 (11)	79 (11)	82 (11)	0.072
t2d	119 (4.0)	317 (4.1)	209 (4.21)	60 (4.8)	0.898	68 (5.7)	65 (4.4)	28 (5.6)	1 (2.4)	0.248
htn	1819 (61.8)	4848 (63.0)	3063 (62.3)	802 (64.0)	0.439	705 (59.1)	883 (59.7)	266 (53.9)	28 (66.7)	0.066
htn_meds	672 (22.7)	1775 (23.0)	1086 (22.0)	296 (23.6)	0.425	261 (21.9)	371 (25.0)	97 (19.5)	10 (23.8)	**0.023**
lipid-lowering	644 (25.4)	1746 (26.1)	1119 (26.1)	303 (24.2)	0.762	273 (26.8)	347 (28.3)	116 (26.9)	8 (19.1)	0.702
ish	48 (1.6)	134 (1.7)	93 (1.9)	16 (1.3)	0.681	11 (0.9)	25 (1.7)	6 (1.2)	0 (0)	0.223
mm_mean_imt	0.689 (0.129)	0.685 (0.126)	0.682 (0.124)	0.688 (0.128)	0.183	0.670 (0.124)	0.671 (0.116)	0.664 (0.122)	0.670 (0.120)	0.167
mm_max_imt	0.923 (0.215)	0.917 (0.208)	0.913 (0.2009	0.921 (0.208)	0.419	0.897 (0.202)	0.903 (0.120)	0.889 (0.209)	0.920 (0.225)	0.105
current smoking	89 (3.0)	254 (3.3)	163 (3.3)	42 (3.4)	0.653	56 (4.7)	77 (5.2)	25 (5.0)	2 (4.8)	0.887
Ever smoker	1041 (35.1)	2853 (36.8)	1804 (36.3)	491 (39.2)	0.285	499 (41.7)	609 (40.9)	189 (37.9)	22 (52.4)	0.442

Where: *, adjusted to provide estimates of treatment-naïve levels; na, not available

STable 3: SNPs in loci associated with SCZ, MDD or BD available on the CardioMetabo and Immuno chips

SCZ			MDD			BD		
Chr	SNP	BP	Chr	SNP	BP	Chr	SNP	BP
1	rs10910077	2390331	1	rs11587140	8260834	1	rs11205277	149892872
1	rs2252865	8422676	1	rs11121129	8268095	1	rs11205303	149906413
1	rs10779702	8423510	1	rs17032305	8270490	1	rs113759410	149907259
1	rs894875	8432136	1	rs11589993	8284497	1	rs4926400	149931541
1	rs6678140	8436802	1	rs423909	8285186	1	rs10888575	150169211
1	rs2708633	8439625	1	rs11121152	8357221	1	rs12086155	150189738
1	rs3765971	8445360	1	rs12711517	8394236	1	rs6679147	150192450
1	rs2784736	8460284	1	rs10399665	8407287	1	rs6655935	150210538
1	rs301795	8467353	1	rs2252865	8422676	1	rs12139286	150262455
1	rs301789	8469337	1	rs10779702	8423510	1	rs17648797	150338002
1	rs1763839	8473336	1	rs894875	8432136	2	rs4907237	97190497
1	rs301798	8488565	1	rs6678140	8436802	2	rs6576973	97218367
1	rs172531	8495590	1	rs2708633	8439625	2	rs7599598	97351840
1	rs301801	8495945	1	rs3765971	8445360	2	rs12466906	97359442
1	rs17385721	8543081	1	rs2784736	8460284	2	rs2872633	97366599
1	rs10798927	30413903	1	rs301795	8467353	2	rs3731940	97377883
1	rs1498243	30415026	1	rs301789	8469337	2	rs6758151	97388884
1	rs11583665	30419456	1	rs1763839	8473336	2	rs6727384	97400324
1	rs267700	30430366	1	rs301798	8488565	2	rs2271893	97405440
1	rs1009080	30431560	1	rs172531	8495590	2	rs6746896	97410949
1	rs4949526	30432219	1	rs301801	8495945	2	rs2314398	97413488
1	rs6694545	30437268	1	rs17385721	8543081	2	rs968470	97423626
1	rs3001723	44037685	1	rs10864360	8724522	2	rs7586317	97447831
1	rs11210892	44100084	1	rs10864361	8726721	2	rs6576984	97454705
1	rs660899	44117006	1	rs2630391	72564879	2	rs7608661	97459920
1	rs6424546	73776673	1	rs11209910	72565630	2	rs1318597	97463095
1	rs17568889	73847888	1	rs1905978	72565800	2	rs7582249	97463529
1	rs12120482	73849893	1	rs17588241	72567009	2	rs4907251	97484814
1	rs10875071	97814678	1	rs17588282	72567065	2	rs10182965	97562551
1	rs4950028	97821081	1	rs10493493	72569309	2	rs7589232	97566148
1	rs12563828	97830721	1	rs2630392	72569613	2	rs7592101	97579507
1	rs4378243	98395881	1	rs2821251	72569980	2	rs17829596	165985503
1	rs10875121	98417446	1	rs11589787	72570425	2	rs10930152	166001183
1	rs11165934	98417967	1	rs1486091	72571364	2	rs7586796	166014227
1	rs9887831	98431639	1	rs1352082	72571656	2	rs10803795	166109764
1	rs12067700	98436232	1	rs2821249	72571841	2	rs7600082	166117860
1	rs12067567	98441888	1	rs78851102	72572054	2	rs353112	166137083
1	rs11165939	98443509	1	rs2630393	72572724	2	rs1849811	166278012
1	rs4573558	98456907	1	rs113385727	72572991	2	rs17249048	166368762
1	rs1938570	98461954	1	rs1486093	72573363	2	rs2221999	166383350
1	rs10747495	98470605	1	rs1564970	72573798	2	rs10427253	166399158
1	rs9324387	98488030	1	rs56062633	72574617	2	rs2581102	194399232
1	rs2391905	98489735	1	rs2821248	72575560	2	rs2581113	194446558
1	rs1938568	98491732	1	rs10789333	72577056	2	rs1585653	194458310
1	rs1625579	98502934	1	rs2630395	72577177	2	rs16835263	194479605

1	rs2660304	98512127	1	rs142453386	72577736	2	rs4531946	194664395
1	rs1702292	98524960	1	rs34709497	72577950	2	rs13418354	194675382
1	rs1702291	98526167	1	rs2821245	72578695	2	rs7606858	194681933
1	rs2660300	98528211	1	rs12402629	72578709	3	rs7616895	36643821
1	rs2660299	98528452	1	rs2821244	72579146	3	rs17035408	36687900
1	rs1782812	98547502	1	rs12124902	72579373	3	rs12107591	36751198
1	rs1198588	98552832	1	rs12026323	72579734	3	rs9820240	36796647
1	rs10888575	150169211	1	rs1486095	72580511	3	rs7620657	36800257
1	rs12086155	150189738	1	rs2630396	72580670	3	rs4678902	36802741
1	rs6679147	150192450	1	rs188374711	72580896	3	rs17035576	36817025
1	rs6655935	150210538	1	rs76828664	72581409	3	rs1876471	36819260
1	rs6670165	177280121	1	rs2630397	72581966	3	rs7624701	36826151
1	rs2761437	207923081	1	rs2630399	72582554	3	rs1507870	36830901
1	rs2796268	207925192	1	rs77944304	72582779	3	rs13072940	36842623
1	rs2724384	207930203	1	rs1385912	72583375	3	rs931913	36843149
1	rs4844390	207934849	1	rs61765575	72583521	3	rs11707149	36850490
1	rs17006738	207936103	1	rs2630400	72583763	3	rs925135	36854253
1	rs6671947	207936491	1	rs1486096	72584184	3	rs9834970	36856030
1	rs11118555	207940853	1	rs116419443	72584230	3	rs906482	36856328
1	rs11118580	207959070	1	rs7549778	72585353	3	rs1553656	36859705
1	rs12568382	207966393	1	rs78590455	72585560	3	rs4072458	36862680
1	rs2294911	207988603	1	rs2821241	72586952	3	rs4624519	36862980
1	rs2235768	207993339	1	rs7556006	72587186	3	rs9917659	36864160
1	rs2490393	243522906	1	rs12128707	72588119	3	rs4789	36869439
1	rs10803138	243555219	1	rs114030538	72588658	3	rs11129735	36870230
1	rs7513566	243588601	1	rs17588812	72588669	3	rs7652637	36871507
1	rs10927013	243589156	1	rs2821239	72590193	3	rs12637912	36871894
1	rs12140431	243596284	1	rs2630404	72590286	3	rs3732386	36871993
1	rs2039839	243598670	1	rs2630405	72590672	3	rs3796186	36876154
1	rs6703335	243608967	1	rs2630406	72590865	3	rs9811916	36878086
1	rs3006925	243609927	1	rs72682716	72590964	3	rs4678909	36878563
1	rs3006917	243641971	1	rs2821238	72591103	3	rs4234258	36878970
1	rs1121276	243710407	1	rs2821237	72591526	3	rs9821223	36883369
1	rs1352162	243717682	1	rs116766293	72591856	3	rs9882911	36885392
1	rs10927039	243721726	1	rs4649954	72593174	3	rs4678910	36887973
1	rs320308	243780198	1	rs12141628	72593767	3	rs17807744	36894048
1	rs4614244	244000874	1	rs115554598	72594798	3	rs11706780	36896235
2	rs1460255	57978858	1	rs2821236	72595125	3	rs11129743	36963420
2	rs11682175	57987593	1	rs12132602	72597051	3	rs6806158	36966067
2	rs1106090	58068741	1	rs2821235	72599170	3	rs9849555	36968309
2	rs10167352	58099420	1	rs7413319	72599191	3	rs4647206	37037716
2	rs118197020	58130326	1	rs72939385	72601349	3	rs4647226	37043796
2	rs2717001	58137618	1	rs1385911	72601854	3	rs1799977	37053568
2	rs2717002	58142647	1	rs34124399	72603500	3	rs6778735	52565100
2	rs2678880	58158283	1	rs1385921	72606298	3	rs13066644	52565247
2	rs2717023	58161355	1	rs2630437	72606562	3	rs13066389	52565317
2	rs2717024	58161397	1	rs12137663	72607638	3	rs7614424	52566354
2	rs2678889	58164223	1	rs79169630	72608372	3	rs7636227	52566682

2	rs2717031	58166468	1	rs115940302	72608554	3	rs12489732	52566820
2	rs2717040	58169494	1	rs80106349	72608658	3	rs7614981	52566914
2	rs1568253	58170004	1	rs12139652	72609333	3	rs12489828	52567014
2	rs1518395	58208074	1	rs76865614	72609647	3	rs28661185	52567188
2	rs6732310	58210239	1	rs57316908	72609955	3	rs4687626	52569098
2	rs2312147	58222928	1	rs191755848	72611701	3	rs60611300	52569949
2	rs2056610	58334799	1	rs1545933	72611715	3	rs76685449	52570330
2	rs1025756	58334925	1	rs114210627	72611772	3	rs7638808	52572056
2	rs848293	58382490	1	rs11209921	72612371	3	rs6445528	52572447
2	rs10445895	58390538	1	rs11587895	72612615	3	rs150774614	52572844
2	rs17049408	58431129	1	rs56256157	72612638	3	rs60823713	52573101
2	rs17049420	58449838	1	rs7517923	72614077	3	rs74620519	52573350
2	rs6761301	58455672	1	rs11587434	72615118	3	rs1133415	52575831
2	rs17049423	58461903	1	rs2821300	72618918	3	rs7643568	52578474
2	rs13411119	58478179	1	rs17589316	72619339	3	rs6802671	52579288
2	rs11689535	146423803	1	rs2422137	72619961	3	rs3199918	52581435
2	rs6724787	149449185	1	rs2821299	72621151	3	rs2878628	52584715
2	rs10183992	149452801	1	rs12124523	72621463	3	rs2251219	52584787
2	rs1913807	162803308	1	rs76290274	72624560	3	rs1961958	52585990
2	rs4664442	162828001	1	rs11209928	72627031	3	rs60682984	52586125
2	rs2909443	162846439	1	rs2821296	72629795	3	rs78235335	52586331
2	rs116302758	162904013	1	rs77773765	72631108	3	rs2336142	52588655
2	rs13023106	185601503	1	rs2901653	72631846	3	rs80332599	52590568
2	Chr2:185309826	185601581	1	rs2630426	72632796	3	rs6778844	52596398
2	rs76903200	185601641	1	rs2630427	72632799	3	rs33964154	52596914
2	rs10167859	185603817	1	rs2630429	72633545	3	rs2289249	52597664
2	rs79761896	185604892	1	rs2630430	72634140	3	rs13060048	52598608
2	rs13026742	185604982	1	rs1157072	72634912	3	rs13063160	52602274
2	rs13001215	185605401	1	rs2630431	72634986	3	rs6786043	52604861
2	rs6434100	185606004	1	rs34029663	72635339	3	rs10510759	52607288
2	rs62176236	185607418	1	rs12134600	72635996	3	rs79658570	52608529
2	rs34481141	185607757	1	rs116255288	72636440	3	rs74489188	52608672
2	rs2170203	185609004	1	rs61765613	72637154	3	rs77716837	52609520
2	rs116000373	185609452	1	rs2630433	72638228	3	rs2028220	52610513
2	rs1480484	185609510	1	rs2821295	72639856	3	rs34735556	52611554
2	rs1480485	185609755	1	rs12117225	72640353	3	rs11718509	52614670
2	rs115082372	185609871	1	rs79908654	72640442	3	rs11720243	52618018
2	rs62176237	185610069	1	rs2630410	72641885	3	rs75551918	52618197
2	rs72901857	185612603	1	rs2821293	72642650	3	rs7614498	52618941
2	rs114566971	185614749	1	rs61765616	72643496	3	rs12629699	52619836
2	rs13384546	185616128	1	rs12036585	72644116	3	rs185116496	52621825
2	rs13417466	185618111	1	rs10889943	72644212	3	rs3852066	52621839
2	rs76430972	185619139	1	rs12748679	72644585	3	rs2590838	52622086
2	rs191173608	185619499	1	rs6699841	72645850	3	rs144941471	52622568
2	rs10190062	185621495	1	rs4344270	72645960	3	rs35225119	52623465
2	rs10172363	185623051	1	rs10789334	72647903	3	rs13065744	52623772
2	rs16826189	185623122	1	rs12726084	72649154	3	rs77711437	52624509
2	rs72901873	185625279	1	rs11209935	72650254	3	rs2336146	52626646

2	rs10206254	185626176	1	rs74824226	72650546	3	rs2577831	52628056
2	rs10206426	185626298	1	rs6668604	72650925	3	rs2118540	52629386
2	rs2369595	185626426	1	rs78828429	72651233	3	rs2164884	52629633
2	rs77224444	185627257	1	rs113630461	72652464	3	rs12107484	52632093
2	rs13396662	185627999	1	rs2298152	72654730	3	rs62253703	52632357
2	rs4278895	185629633	1	rs2821291	72654816	3	rs114597651	52634050
2	rs6712125	185629756	1	rs2298151	72655369	3	rs2276824	52637486
2	rs72901885	185630024	1	rs6672863	72655636	3	rs34341044	52637631
2	rs72901889	185632346	1	rs2821290	72656205	3	rs3774366	52641255
2	rs6728615	185632513	1	rs1342862	72657058	3	rs78410573	52641568
2	rs10177035	185633046	1	rs2153929	72658214	3	rs71299610	52641703
2	rs6732176	185633109	1	rs34289700	72660048	3	rs3774365	52642487
2	rs72901891	185633794	1	rs2821288	72661104	3	rs12487591	52642936
2	rs115682664	185633796	1	rs6656980	72661288	3	rs3755806	52643685
2	rs72901893	185634241	1	rs34840702	72662959	3	rs6803492	52649090
2	rs10194277	185634659	1	rs7518919	72663249	3	rs13083798	52649748
2	rs10174301	185635853	1	rs112593979	72663293	3	rs10510760	52650348
2	rs6759491	185636177	1	rs12141242	72663576	3	rs77810318	52650919
2	rs62176239	185636567	1	rs2821287	72663761	3	rs62253734	52653645
2	rs6716151	185636617	1	rs1342874	72664938	3	rs60256157	52656491
2	rs72901901	185637164	1	rs35944729	72666431	3	rs75246353	52656781
2	rs17617913	185638875	1	rs2821285	72667196	3	rs13064064	52657002
2	rs78011041	185639136	1	rs2821284	72669322	3	rs61538294	52659594
2	rs10196799	185640728	1	rs9425076	72669968	3	rs1561337	52659963
2	rs116775825	185641160	1	rs7349073	72670841	3	rs79457385	52660127
2	rs12693389	185641495	1	rs2821283	72673061	3	rs184514749	52661018
2	rs72903709	185641564	1	rs2797540	72673194	3	rs7622694	52663882
2	rs10176668	185641635	1	rs1858601	72674434	3	rs78506463	52664168
2	rs6434102	185642580	1	rs1858600	72674489	3	rs78842975	52667856
2	rs13018902	185642880	1	rs74653947	72676153	3	rs2083180	52668119
2	rs17431119	185642960	1	rs12725081	72682718	3	rs17052357	52668638
2	rs17431132	185644113	1	rs1675356	72683226	3	rs13068293	52672167
2	rs17431153	185644845	1	rs61765640	72684333	3	rs11130312	52675055
2	rs13010468	185645106	1	rs115533964	72686098	3	rs11130313	52676190
2	rs10497657	185645840	1	rs34274959	72686292	3	rs13099479	52677478
2	rs10497658	185646020	1	rs1194286	72688503	3	rs13085895	52678270
2	rs13017390	185646076	1	rs1194285	72689238	3	rs2028216	52680823
2	rs1987025	185647595	1	rs183010826	72689886	3	rs2289250	52682946
2	rs17584494	185647985	1	rs1675353	72691347	3	rs33967311	52683864
2	rs72903727	185648068	1	rs72682744	72692101	3	rs934839	52683898
2	rs10497659	185648256	1	rs12125336	72692630	3	rs80214269	52688746
2	rs116774374	185649149	1	rs34940719	72693786	3	rs12486847	52691112
2	rs10497660	185649196	1	rs1205484	72695547	3	rs2590846	52692359
2	rs1317210	185649555	1	rs78152876	72696649	3	rs56665533	52692725
2	rs1320080	185650005	1	rs77516608	72696845	3	rs78059582	52693216
2	rs17431258	185650079	1	rs11209938	72697178	3	rs6804145	52694198
2	rs72903735	185650668	1	rs985227	72697459	3	rs11715796	52695650
2	rs72903737	185651328	1	rs2797543	72701100	3	rs7651709	52695861

2	rs10195510	185652653	1	rs1194270	72702313	3	rs72950432	52696352
2	rs6716235	185655092	1	rs1194269	72703860	3	rs11130314	52697082
2	rs12693391	185655189	1	rs1194267	72704526	3	rs79993196	52697566
2	rs4635495	185655317	1	rs185945186	72705230	3	rs71299613	52698805
2	rs34327168	185656085	1	rs113371150	72707049	3	rs1992193	52699089
2	rs34963004	185656366	1	rs1194265	72707226	3	rs2577842	52700984
2	rs34452663	185656920	1	rs1026567	72708584	3	rs73078833	52703691
2	rs72903752	185659789	1	rs1026566	72709042	3	rs12496476	52708737
2	rs62176259	185663735	1	rs72682752	72710713	3	rs73078835	52709711
2	rs4294964	185664579	1	rs9425080	72711900	3	rs114180403	52709810
2	rs72903765	185664802	1	rs17525406	72712003	3	rs180905584	52709850
2	rs62178060	185665080	1	rs2066416	72712404	3	rs62255368	52710172
2	rs72903769	185665389	1	rs1194284	72712529	3	rs72950437	52714400
2	rs6751335	185669127	1	rs2126077	72712607	3	rs78892252	52715311
2	rs10211213	185670083	1	rs1194283	72713037	3	rs3733039	52719088
2	rs13012893	185670332	1	rs9424976	72718610	3	rs1108842	52720080
2	rs74961493	185670496	1	rs17591264	72723236	3	rs11177	52721305
2	rs13012514	185672183	1	rs2000228	72723863	3	rs35911561	52722562
2	rs72903776	185672750	1	rs34723450	72724409	3	rs1315803266	52723309
2	rs72903778	185673214	1	rs77642121	72725232	3	rs6762813	52726695
2	rs72903783	185673390	1	rs9424977	72725344	3	rs2289247	52727257
2	rs72903786	185674302	1	rs1620977	72729142	3	rs4532127	52727736
2	rs17509336	185677806	1	rs17525824	72731621	3	rs6976	52728804
2	rs17431278	185678006	1	rs1841500	72732323	3	rs56156188	52730699
2	rs72903792	185679980	1	rs1460947	72732482	3	rs139683420	52730946
2	rs10201360	185680799	1	rs1460948	72732574	3	rs2164885	52731269
2	rs13408744	185681052	1	rs9424978	72732923	3	rs11130317	52731483
2	rs72903798	185681546	1	rs1380995	72733841	3	rs13081155	52732429
2	rs17584522	185681730	1	rs116244409	72735434	3	rs3075809	52733082
2	rs13386789	185681984	1	rs12137231	72735458	3	rs6765687	52733106
2	rs62174661	185687954	1	rs34376163	72738699	3	rs8906	52739520
2	rs35105890	185688786	1	rs12737564	72739527	3	rs6617	52740182
2	rs189530005	185689599	1	rs12127789	72740073	3	rs79633493	52741032
2	rs4544394	185689671	1	rs114308590	72740282	3	rs3755798	52741160
2	rs72905716	185690060	1	rs11584542	72741382	3	rs34017441	52742413
2	rs9973631	185690911	1	rs11584611	72741829	3	rs115738369	52743289
2	rs72905722	185691444	1	rs9425081	72742235	3	rs34182518	52743854
2	rs13424507	185691625	1	rs3101340	72744144	3	rs13079063	52744460
2	rs9973668	185692497	1	rs34874247	72744950	3	rs11235	52745087
2	rs13028349	185693549	1	rs1870676	72744996	3	rs145014080	52747486
2	rs76585359	185695954	1	rs34579341	72745962	3	rs2878726	52748271
2	rs16826219	185696114	1	rs77709389	72746633	3	rs11716747	52748857
2	rs72905730	185696271	1	rs3101341	72747844	3	rs35526119	52749334
2	rs12693395	185696607	1	rs76659454	72749726	3	rs60770874	52750199
2	rs116351004	185697406	1	rs3128341	72749848	3	rs116493126	52750325
2	rs17509517	185700607	1	rs3101338	72750353	3	rs7624716	52751534
2	rs62174663	185700770	1	rs34361149	72750470	3	rs115745575	52753308
2	rs72905736	185701858	1	rs11209943	72750500	3	rs56196917	52756045

2	rs115103722	185702685	1	rs3101337	72751134	3	rs72960240	52759133
2	rs7605689	185705856	1	rs3101336	72751185	3	rs13063138	52759254
2	rs72905739	185706202	1	rs2613499	72751552	3	rs13087538	52763453
2	rs10180597	185708432	1	rs2815765	72752230	3	rs6769720	52764350
2	rs7603015	185708987	1	rs2613498	72752939	3	rs10780035	52764624
2	rs10187190	185710178	1	rs61765650	72753112	3	rs73078847	52764787
2	rs72905751	185713166	1	rs2568953	72753956	3	rs2268027	52766606
2	rs72905753	185713304	1	rs61765651	72754314	3	rs767418	52767427
2	rs57491014	185717491	1	rs2613496	72754788	3	rs1231194	52769047
2	rs7578316	185718167	1	rs2568952	72755105	3	rs11130323	52770277
2	rs80268465	185719122	1	rs114171655	72759564	3	rs56276589	52772198
2	rs12693396	185719174	1	rs2815757	72764289	3	rs34184137	52774017
2	rs75530945	185720402	1	rs2568956	72764376	3	rs75321479	52778210
2	rs55941754	185720926	1	rs2568957	72764430	3	rs2268026	52778347
2	rs72905773	185721156	1	rs78778221	72764809	3	rs60058353	52778409
2	rs13386192	185722380	1	rs9425089	72765082	3	rs2072390	52780509
2	rs72905777	185722863	1	rs2568958	72765116	3	rs6762788	52792311
2	rs7593307	185724193	1	rs9425090	72765300	3	rs1029871	52797634
2	rs12693397	185724435	1	rs9424981	72766085	3	rs72960281	52802871
2	rs72905782	185724464	1	rs185397415	72811839	3	rs13071584	52804487
2	rs72905784	185724642	1	rs11209948	72811904	3	rs998909	52805093
2	rs13396624	185725062	1	rs78095791	72811957	3	rs11717836	52807736
2	rs72905785	185725119	1	rs2815753	72812324	3	rs72960285	52807816
2	rs72905786	185725574	1	rs2815752	72812440	3	rs1014969	52808341
2	rs7585171	185725617	1	rs2613494	72812657	3	rs3755799	52809193
2	rs72905792	185726138	1	rs2568961	72812747	3	rs2019065	52809525
2	rs72905794	185726221	1	rs6682389	72813057	3	rs62255400	52812231
2	rs62200788	185727845	1	rs1460943	72813129	3	rs75097760	52814643
2	rs72905797	185727861	1	rs1432639	72813218	3	rs36012032	52814709
2	rs7558878	185729162	1	rs1460942	72813256	3	rs2710323	52815905
2	rs11890843	185729304	1	rs1460940	72814617	3	rs187624109	52816588
2	rs13398732	185730587	1	rs2815749	72814783	3	rs2710322	52817593
2	rs17509601	185731901	1	rs2815748	72816147	3	rs3774354	52817675
2	rs17509608	185732578	1	rs6690399	72817099	3	rs3774355	52817778
2	rs13401381	185734749	1	rs17526561	72818226	3	rs11715487	52818301
2	rs7588907	185735599	1	rs2012697	72819612	3	rs2239551	52818579
2	rs17509622	185735643	1	rs2815747	72821126	3	rs2268023	52819327
2	rs7564941	185735813	1	rs12143571	72822096	3	rs2535630	52819883
2	rs10199843	185736625	1	rs12117438	72822479	3	rs678	52820981
2	rs6709436	185738633	1	rs137935824	72822917	3	rs1042779	52821011
2	rs11902536	185738735	1	rs990871	72823713	3	rs2286798	52821177
2	rs1366845	185739226	1	rs6687024	72824860	3	rs111477914	52821666
2	rs77689531	185741079	1	rs12143898	72826172	3	rs68094128	52821993
2	rs17431603	185741744	1	rs78120369	72826945	3	rs2239550	52822509
2	rs115166888	185741898	1	rs6660464	72826949	3	rs2300149	52822921
2	rs72907715	185742038	1	rs76611872	72827037	3	rs2239549	52823126
2	rs1366843	185742271	1	rs76455638	72827501	3	rs4687550	52823430
2	rs1820847	185742474	1	rs12139531	72827776	3	rs4687551	52823448

2	rs4586602	185742794	1	rs115720372	72829015	3	rs2270197	52824095
2	rs72907717	185742905	1	rs11581382	72829347	3	rs1076425	52825462
2	rs13411652	185743241	1	rs11209950	72829680	3	rs1075653	52825528
2	rs1429427	185743953	1	rs72943001	72831388	3	rs9324	52825585
2	rs34233935	185744192	1	rs12123328	72833201	3	rs7549	52825912
2	rs10206265	185744352	1	rs74820801	72833505	3	rs2071506	52826276
2	rs112869258	185745353	1	rs2613505	72835410	3	rs746694	52826620
2	rs112372472	185745500	1	rs2613504	72835740	3	rs2071507	52826707
2	rs72907724	185748032	1	rs7531118	72837239	3	rs2071508	52826846
2	rs72907728	185749875	1	rs11209951	72837490	3	rs4687654	52827566
2	rs34044253	185750030	1	rs72684612	72837777	3	rs2239699	52827915
2	rs11899082	185754482	1	rs10789336	72838406	3	rs9881468	52828628
2	rs114385979	185754801	1	rs1993709	72838529	3	rs56009954	52829607
2	rs150700380	185755435	1	rs4322186	72838561	3	rs2240921	52830764
2	rs13429132	185756725	1	rs2613503	72839774	3	rs2240920	52831009
2	rs13428955	185756744	1	rs79547900	72841201	3	rs62255413	52831386
2	rs12693399	185757011	1	rs72684614	72842892	3	rs2240919	52831701
2	rs12693400	185758412	1	rs72945111	72845176	3	rs2535629	52833219
2	rs6715910	185758901	1	rs72945113	72845264	3	rs3617	52833805
2	rs34700435	185758973	1	rs114116549	72859448	3	rs9848554	52836337
2	rs12999259	185759675	1	rs114950370	72860308	3	rs17331151	52844534
2	rs113113034	185760985	1	rs1460939	72861567	3	rs4687657	52852538
2	rs6718067	185761264	1	rs12123766	72861847	3	rs2239547	52855229
2	rs72907741	185761731	1	rs7551291	72865128	3	rs2276816	52860816
2	rs7584503	185761922	1	rs1599334	72866574	3	rs2276817	52860936
2	rs116715697	185762217	1	rs78086129	72867555	3	rs4687554	52864135
2	rs6722425	185762587	1	rs115781967	72871716	3	rs13083728	52868445
2	rs6710286	185762811	1	rs76827956	72872889	3	rs1573815	52870132
2	rs3931790	185763376	1	rs1838662	72874257	3	rs1139106	52871101
2	rs7562384	185763445	1	rs12406365	72874258	3	rs3733047	52871929
2	rs72907747	185765063	1	rs12139940	72875061	3	rs6445539	52874296
2	rs1344707	185766001	1	rs10789338	72875201	3	rs11720228	52877270
2	rs7603001	185766816	1	rs11209961	72875646	3	rs13080929	52885112
2	rs7590852	185766912	1	rs290703	72876286	3	rs3796352	52913279
2	rs12693401	185767283	1	rs290702	72876346	3	rs3733034	52952433
2	rs72907756	185767291	1	rs115771589	72877798	3	rs10510761	52965255
2	rs1366840	185767854	1	rs17092655	72877899	3	rs11710394	53015839
2	rs10931155	185768559	1	rs72684636	72878664	3	rs2581824	53022408
2	rs72907759	185769129	1	rs17092663	72879614	3	rs2255107	53060637
2	rs72907760	185769130	1	rs115781252	72880148	3	rs9847710	53062661
2	rs10497661	185769199	1	rs17092672	72880428	3	rs2581806	53063360
2	rs77076543	185769746	1	rs7532281	72882343	3	rs697962	107618914
2	rs10497662	185769921	1	rs115335794	72882463	3	rs709514	107620896
2	rs1835172	185770241	1	rs11209963	72883303	3	rs4855779	107646728
2	rs4666994	185771745	1	rs76696481	72884606	3	rs9845309	107708956
2	rs4666995	185771790	1	rs10789339	72884712	3	rs1344877	107737386
2	rs115515236	185772643	1	rs11209964	72885087	3	rs6790456	107739094
2	rs72907766	185772976	1	rs11209964	72885098	3	rs7622560	107744023

2	rs66970879	185773132	1	rs2590942	72885281	3	rs11712272	107747672
2	rs66726164	185773141	1	rs7549534	72885969	3	rs7635063	107818777
2	rs66473204	185773243	1	rs6673910	72886559	3	rs7640424	107820063
2	rs17431742	185773401	1	rs112265677	72887660	3	rs17828045	107821546
2	rs1114248	185774139	1	rs116152102	72896151	3	rs6795280	107876232
2	rs78773484	185777032	1	rs79499349	72896317	3	rs12632400	107941614
2	rs72893066	185778156	1	rs72945146	72896381	3	rs12493468	108012420
2	rs725617	185778262	1	rs2422221	72896654	4	rs1523562	162088541
2	rs1344706	185778428	1	rs980243	72897106	4	rs2872188	162107994
2	rs13009002	185778602	1	rs112590630	72897477	4	rs13110266	162129844
2	rs72893068	185778772	1	rs77981679	72897673	4	rs6536580	162134859
2	rs115456739	185779904	1	rs12143162	72897687	4	rs1444285	162163105
2	rs4666998	185780221	1	rs1445582	72897985	4	rs7700001	162200085
2	rs13423388	185780225	1	rs76195116	72898425	4	rs9308024	162207646
2	rs72893071	185781197	1	rs782257	72899059	4	rs12510294	162234287
2	rs62198463	185781300	1	rs113837642	72899299	4	rs976972	162244482
2	rs73043287	185782186	1	rs1445581	72899359	4	rs7689446	162263362
2	rs1583048	185783141	1	rs12404629	72899477	4	rs2314143	162282165
2	rs1366839	185783666	1	rs74909863	72900414	4	rs12505871	162369349
2	rs1366838	185783896	1	rs992956	72901141	4	rs12512161	162371431
2	rs115151279	185784128	1	rs114086144	72901383	4	rs17041096	162383466
2	rs17662626	193984621	1	rs12133881	72906742	4	rs4373120	162411391
2	rs6738721	198182334	1	rs2121056	72906786	4	rs4518198	162426629
2	rs6434926	198185078	1	rs782239	72908137	4	rs16999330	162460563
2	rs3097385	198199297	1	rs111616364	72908144	4	rs4501178	162477082
2	rs3849361	198288826	1	rs112397929	72908616	4	rs10517746	162521710
2	rs3731570	198317783	1	rs7534013	72909011	5	rs1505088	7360543
2	rs17731449	198465840	1	rs4649956	72909435	5	rs12514566	7391462
2	rs2045245	198485341	1	rs12026130	72910098	5	rs13153439	7398546
2	rs13000656	198522126	1	rs10889955	72910182	5	rs7733194	7441829
2	rs10497807	198585087	1	rs782252	72911829	5	rs17228455	7458019
2	rs13012441	198603121	1	rs12119736	72912656	5	rs7712809	7459043
2	rs13425988	198622378	1	rs7535539	72913499	5	rs17229159	7475407
2	rs700648	198622636	1	rs7547113	72913603	5	rs10462842	7517897
2	rs700651	198631714	1	rs111760100	72915131	5	rs2173701	7540884
2	rs4550664	198691405	1	rs112914908	72915699	5	rs7720767	7546394
2	rs700680	198707950	1	rs113421220	72915720	5	rs7700862	7646404
2	rs700688	198723833	1	rs782246	72915804	5	rs2973322	7652518
2	rs4850812	198743655	1	rs782245	72915844	5	rs877886	7659279
2	rs1464211	198798630	1	rs782243	72916538	5	rs1428526	7689267
2	rs1518367	198807015	1	rs782242	72916574	5	rs4235587	7734493
2	rs2166517	200190213	1	rs78931181	72916577	5	rs1460970	7794738
2	rs1348812	200236425	1	rs12759396	72921381	5	rs6864771	7797291
2	rs16831510	200305460	1	rs75365549	72921464	5	rs16879141	7801371
2	rs17590956	200720420	1	rs77328742	72923408	5	rs13162758	7801608
2	rs17529746	200726221	1	rs782221	72924057	5	rs3756636	80546499
2	rs769950	200730028	1	rs78568434	72924558	5	rs1039326	80609768
2	rs281783	200751582	1	rs4650138	72924689	5	rs6885028	80612085

2	rs1124639	200775744	1	rs782219	72924810	5	rs10056565	80619046
2	rs10498163	225434445	1	rs6698502	72925024	5	rs6881927	80629372
2	rs12052869	233591551	1	rs782218	72925397	5	rs6452399	80631511
2	rs8192539	233632766	1	rs61771778	72926855	5	rs10371	80631642
2	rs7593642	233682722	1	rs1838658	72928467	5	rs6885431	80633196
2	rs6751801	233697200	1	rs79334904	72930595	5	rs4235653	80637529
2	rs778341	233728801	1	rs7534853	72934011	5	rs7735423	80644174
2	rs2675968	233736244	1	rs6692258	72934320	5	rs10942262	80675496
2	rs778370	233743363	1	rs10889956	72936345	5	rs12187063	80871602
2	rs709937	233744021	1	rs1822981	72936795	5	rs7720445	80901915
2	rs2675954	233750627	1	rs1822980	72936801	5	rs447574	80937152
3	rs17620999	2561556	1	rs949773	72937078	6	rs10942953	72269473
3	rs11128821	17330693	1	rs1807758	72937523	6	rs2087092	72269581
3	rs283932	17364413	1	rs7530496	72937842	6	rs72651689	72269625
3	rs17200795	17402592	1	rs10889957	72938027	6	rs80295642	72270151
3	rs12496758	17644775	1	rs10493498	72938249	6	rs61160900	72270782
3	rs7620425	17848474	1	rs1418621	72938679	6	rs10455238	72271845
3	rs11707149	36850490	1	rs7536869	72939855	6	rs72651700	72273244
3	rs925135	36854253	1	rs10789340	72940273	6	rs72651701	72273258
3	rs9834970	36856030	1	rs61771792	72940536	6	rs28822989	72273801
3	rs906482	36856328	1	rs80022132	72941784	6	rs12216174	72274288
3	rs1553656	36859705	1	rs74089203	72941970	6	rs72653105	72274711
3	rs4072458	36862680	1	rs112552181	72942362	6	rs116067179	72274843
3	rs4624519	36862980	1	rs80214517	72942781	6	rs79206107	72274870
3	rs9917659	36864160	1	rs12408088	72942878	6	rs60965402	72275033
3	rs4789	36869439	1	rs79197341	72944025	6	rs75117691	72275124
3	rs11129735	36870230	1	rs7550173	72944617	6	rs59637520	72275466
3	rs7652637	36871507	1	rs11209972	72944714	6	rs2101382	72275927
3	rs12637912	36871894	1	rs12033462	72947342	6	rs72653112	72276608
3	rs3732386	36871993	1	rs75129760	72947691	6	rs1855431	72278314
3	rs3796186	36876154	1	rs1031188	72948540	6	rs78851178	72278433
3	rs9811916	36878086	1	rs17092792	72948800	6	rs11963624	72278541
3	rs4678909	36878563	1	rs75324010	72949049	6	rs76683562	72278990
3	rs4234258	36878970	1	rs707444	72949290	6	rs9358960	72279021
3	rs9821223	36883369	1	rs78506678	72949727	6	rs77354299	72279402
3	rs9882911	36885392	1	rs4649957	72949748	6	rs72653118	72279846
3	rs4678910	36887973	1	rs1822979	72951344	6	rs4707922	72281932
3	rs17807744	36894048	1	rs17097721	72951777	6	rs16881513	72282786
3	rs11706780	36896235	1	rs78765738	72952053	6	rs9342853	72282925
3	rs41292372	52541937	1	rs17092800	72952117	6	rs9351828	72286607
3	rs1010554	52542919	1	rs782208	72953022	6	rs6914250	72286838
3	rs9835659	52544470	1	rs782207	72953251	6	rs72653119	72287771
3	rs115217609	52545370	1	rs76179389	72953323	6	rs16881519	72288333
3	rs7644763	52546487	1	rs74546891	72953435	6	rs80241482	72289367
3	rs2015971	52546820	1	rs813004	72954611	6	rs6927647	72289651
3	rs740903	52548818	1	rs2218500	72954641	6	rs1486596	72290080
3	rs41292858	52548873	1	rs6692386	72954883	6	rs12210828	72290462
3	rs79979130	52551010	1	rs4545290	72955823	6	rs75793671	72290713

3	rs66824127	52551265	1	rs10493499	72955990	6	rs9446417	72291479
3	rs67409736	52551284	1	rs812462	72956037	6	rs9446418	72291534
3	rs56960576	52553167	1	rs782205	72956078	6	rs12214683	72292465
3	rs186689880	52553628	1	rs61771794	72956259	6	rs60181928	72292687
3	rs191127022	52553629	1	rs61771795	72956912	6	rs1032166	72293273
3	rs13081028	52555316	1	rs61771798	72958702	6	rs12528750	72293746
3	rs9853056	52555957	1	rs72684685	72958905	6	rs12529063	72293855
3	rs35325270	52556369	1	rs782233	72984094	6	rs6925362	72294350
3	rs4434138	52556890	1	rs17543217	72994090	6	rs62404288	72295061
3	rs4234633	52557038	1	rs782262	73009278	6	rs12183351	72295183
3	rs13303	52558008	1	rs12142193	73025079	6	rs78814809	72295211
3	rs13621	52558133	1	rs4130885	73575075	6	rs73750712	72296032
3	rs142803792	52558768	1	rs6424546	73776673	6	rs76018244	72297198
3	rs34005367	52558904	1	rs17568889	73847888	6	rs72925179	72298195
3	rs11709284	52559705	1	rs12120482	73849893	6	rs12193019	72300534
3	rs4475032	52560021	1	rs6424689	80556027	6	rs9341328	72301665
3	rs187437419	52560073	1	rs11162919	80556815	6	rs11756168	72303001
3	rs192930569	52560074	1	rs4483362	80624295	6	rs9351832	72303339
3	rs11711421	52561779	1	rs4335329	80633312	6	rs1486605	72303559
3	rs35920544	52563199	1	rs6424696	80641631	6	rs12193188	72303915
3	rs35371456	52563462	1	rs12568437	80681855	6	rs9293845	72304241
3	rs4687624	52563572	1	rs1687845	80682411	6	rs9342854	72304672
3	rs4687625	52563718	1	rs1416467	80758728	6	rs1486606	72305075
3	rs6778735	52565100	1	rs951258	80836881	6	rs1486607	72305129
3	rs13066644	52565247	1	rs1781762	80838458	6	rs7775155	72305735
3	rs13066389	52565317	1	rs2997195	80838880	6	rs9342855	72306160
3	rs7614424	52566354	1	rs184812	80970978	6	rs1156409	72306468
3	rs7636227	52566682	1	rs356287	80977963	6	rs9360468	72306718
3	rs12489732	52566820	1	rs7520257	90600817	6	rs72938847	72307811
3	rs7614981	52566914	1	rs4269759	90601373	6	rs67041987	72308014
3	rs12489828	52567014	1	rs6428576	90607718	6	rs139792318	72308871
3	rs28661185	52567188	1	rs7526266	90625368	6	rs56680683	72309435
3	rs4687626	52569098	1	rs17463487	90625731	6	rs9358965	72312071
3	rs60611300	52569949	1	rs10922788	90640333	6	rs9360472	72313523
3	rs76685449	52570330	1	rs6428581	90656996	6	rs9442685	72313929
3	rs7638808	52572056	1	rs4415546	90658051	6	rs9341329	72314189
3	rs6445528	52572447	1	rs12410130	90686440	6	rs6908161	72315834
3	rs150774614	52572844	1	rs6428583	90693880	6	rs12663567	72316008
3	rs60823713	52573101	1	rs12117300	90740316	6	rs4707928	72316230
3	rs74620519	52573350	1	rs12743329	90757360	6	rs9342857	72316549
3	rs1133415	52575831	1	rs12736146	90762510	6	rs9351834	72317048
3	rs7643568	52578474	1	rs12747733	90766521	6	rs3920742	72317266
3	rs6802671	52579288	1	rs12407088	90776860	6	rs75614833	72318428
3	rs3199918	52581435	1	rs12754978	90814006	6	rs9293846	72319044
3	rs2878628	52584715	1	rs12734840	90944431	6	rs12211666	72319079
3	rs2251219	52584787	1	rs17131081	91009841	6	rs75791845	72319269
3	rs1961958	52585990	1	rs3886600	197504855	6	rs16881625	72319359
3	rs60682984	52586125	1	rs115834485	197505460	6	rs982254	72319782

3	rs78235335	52586331	1	rs4026513	197505890	6	rs2881952	72320145
3	rs2336142	52588655	1	rs10494757	197507411	6	rs6453557	72320187
3	rs80332599	52590568	1	rs4915550	197508265	6	rs34273564	72321017
3	rs6778844	52596398	1	rs12094881	197508544	6	rs720742	72321902
3	rs33964154	52596914	1	rs4915551	197508901	6	rs720741	72321961
3	rs2289249	52597664	1	rs17621768	197509624	6	rs9360475	72323643
3	rs13060048	52598608	1	rs12118913	197512404	6	rs982564	72323850
3	rs13063160	52602274	1	rs4915552	197512778	6	rs982565	72323926
3	rs6786043	52604861	1	rs10754224	197512789	6	rs16881643	72324280
3	rs10510759	52607288	1	rs116135944	197512851	6	rs1486613	72326511
3	rs79658570	52608529	1	rs79558293	197512914	6	rs9342860	72327032
3	rs74489188	52608672	1	rs4303099	197515715	6	rs9351837	72327545
3	rs77716837	52609520	1	rs10922249	197515981	6	rs10942956	72327755
3	rs2028220	52610513	1	rs12118513	197517086	6	rs12197344	72327809
3	rs34735556	52611554	1	rs10801614	197520273	6	rs9293847	72327933
3	rs11718509	52614670	1	rs6677361	197521877	6	rs6906594	72328062
3	rs11720243	52618018	1	rs6688993	197522671	6	rs13195852	72328482
3	rs75551918	52618197	1	rs12028758	197522958	6	rs6453558	72329609
3	rs7614498	52618941	1	rs16841715	197527261	6	rs829273	72329791
3	rs12629699	52619836	1	rs10922253	197527615	6	rs1889276	72330088
3	rs185116496	52621825	1	rs10494758	197528149	6	rs1889277	72330196
3	rs3852066	52621839	1	rs6682689	197528329	6	rs12212596	72330417
3	rs2590838	52622086	1	rs6691173	197528488	6	rs12212719	72330453
3	rs144941471	52622568	1	rs12563879	197528805	6	rs829276	72331044
3	rs35225119	52623465	1	rs2111931	197529218	6	rs17550741	72331861
3	rs13065744	52623772	1	rs12731480	197529264	6	rs272240	72333143
3	rs77711437	52624509	1	rs12125739	197529577	6	rs12374601	72333794
3	rs2336146	52626646	1	rs34261593	197530702	6	rs272234	72335658
3	rs2577831	52628056	1	rs74461411	197531697	6	rs10485019	72336534
3	rs2118540	52629386	1	rs6689216	197536225	6	rs10942957	72337017
3	rs2164884	52629633	1	rs4026508	197536424	6	rs118081514	72337310
3	rs12107484	52632093	1	rs10922255	197537029	6	rs9442688	72339566
3	rs62253703	52632357	1	rs10922256	197545307	6	rs10455241	72341858
3	rs114597651	52634050	1	rs10922257	197545720	6	rs166621	72342533
3	rs2276824	52637486	1	rs1998711	197546093	6	rs9446428	72343558
3	rs34341044	52637631	1	rs2016722	197546459	6	rs1575208	72346472
3	rs3774366	52641255	1	rs6428410	197546891	6	rs59328205	72347297
3	rs78410573	52641568	1	rs10737692	197548933	6	rs272206	72348344
3	rs71299610	52641703	1	rs4915554	197549551	6	rs1321831	72349268
3	rs3774365	52642487	1	rs10922259	197549777	6	rs13210716	72349628
3	rs12487591	52642936	1	rs193149367	197551514	6	rs16881676	72349689
3	rs3755806	52643685	1	rs2884629	197552034	6	rs16881677	72350814
3	rs6803492	52649090	1	rs113633618	197552813	6	rs9351839	72351169
3	rs13083798	52649748	1	rs2193733	197554134	6	rs717519	72351393
3	rs10510760	52650348	1	rs78636848	197554302	6	rs35525158	72352061
3	rs77810318	52650919	1	rs72742683	197554943	6	rs6912835	72352079
3	rs62253734	52653645	1	rs10922260	197555404	6	rs112249916	72355142
3	rs60256157	52656491	1	rs16841765	197555761	6	rs272212	72355435

3	rs75246353	52656781	1	rs11583319	197555860	6	rs80112601	72356119
3	rs13064064	52657002	1	rs10922261	197557272	6	rs9442689	72356347
3	rs61538294	52659594	1	rs2193734	197557552	6	rs272211	72356738
3	rs1561337	52659963	1	rs75007359	197559290	6	rs9446432	72356760
3	rs79457385	52660127	1	rs6702421	197559324	6	rs12194008	72359600
3	rs184514749	52661018	1	rs10922263	197559836	6	rs72940808	72361341
3	rs7622694	52663882	1	rs78259147	197560252	6	rs13190763	72362565
3	rs78506463	52664168	1	rs17622452	197560485	6	rs9351841	72363324
3	rs78842975	52667856	1	rs2111934	197560962	6	rs117855045	72363381
3	rs2083180	52668119	1	rs8179369	197561407	6	rs7743293	72363620
3	rs17052357	52668638	1	rs35939830	197561510	6	rs80149900	72364273
3	rs13068293	52672167	1	rs12141028	197562511	6	rs272213	72365557
3	rs11130312	52675055	1	rs6667685	197563333	6	rs16881689	72366341
3	rs11130313	52676190	1	rs4274099	197563701	6	rs9342864	72366851
3	rs13099479	52677478	1	rs12747786	197565832	6	rs9293848	72367279
3	rs13085895	52678270	1	rs10922264	197566414	6	rs76029558	72369567
3	rs2028216	52680823	1	rs16841794	197568270	6	rs9442690	72369851
3	rs2289250	52682946	1	rs6428411	197569657	6	rs117502260	72371452
3	rs33967311	52683864	1	rs6428412	197570018	6	rs2200526	72372492
3	rs934839	52683898	1	rs114138649	197570517	6	rs2200527	72372493
3	rs80214269	52688746	1	rs12144001	197570844	6	rs189590254	72373070
3	rs12486847	52691112	1	rs7544969	197571246	6	rs11755205	72373830
3	rs2590846	52692359	1	rs1833464	197571577	6	rs272226	72375097
3	rs56665533	52692725	1	rs7531581	197571885	6	rs1547046	72375154
3	rs78059582	52693216	1	rs12142712	197572672	6	rs2200528	72375836
3	rs6804145	52694198	1	rs115942017	197573021	6	rs9341336	72376785
3	rs11715796	52695650	1	rs17564924	197574134	6	rs9350422	72376933
3	rs7651709	52695861	1	rs972405	197574174	6	rs75519891	72377018
3	rs72950432	52696352	1	rs116759938	197574231	6	rs12173946	72377779
3	rs11130314	52697082	1	rs116567755	197577447	6	rs9351843	72378029
3	rs79993196	52697566	1	rs7548454	197578795	6	rs272229	72378469
3	rs71299613	52698805	1	rs79627522	197578878	6	rs74787814	72378588
3	rs1992193	52699089	1	rs112883537	197579127	6	rs10942960	72379124
3	rs2577842	52700984	1	rs1362939	197580084	6	rs9442691	72379528
3	rs73078833	52703691	1	rs12116508	197580452	6	rs77081319	72381439
3	rs12496476	52708737	1	rs10922266	197582447	6	rs73525252	72381632
3	rs73078835	52709711	1	rs4915557	197582601	6	rs2840983	72382345
3	rs114180403	52709810	1	rs11799915	197582780	6	rs74541915	72382501
3	rs180905584	52709850	1	rs4915558	197582809	6	rs10223675	72382998
3	rs62255368	52710172	1	rs1969	197583281	6	rs10455242	72383139
3	rs72950437	52714400	1	rs3814321	197583843	6	rs78139980	72384426
3	rs78892252	52715311	1	rs12744898	197584553	6	rs117534741	72384541
3	rs3733039	52719088	1	rs34780947	197585077	6	rs6918162	72384595
3	rs1108842	52720080	1	rs2111930	197586232	6	rs4706488	72385311
3	rs11177	52721305	1	rs12022672	197588006	6	rs9442693	72386001
3	rs35911561	52722562	1	rs76017973	197589075	6	rs272244	72386156
3	rs1315803266	52723309	1	rs4282851	197590190	6	rs2054810	72386408
3	rs6762813	52726695	1	rs10801620	197591406	6	rs79775242	72386598

3	rs2289247	52727257	1	rs114114064	197591828	6	rs62407661	72386761
3	rs4532127	52727736	1	rs12133885	197592568	6	rs10455243	72389035
3	rs6976	52728804	1	rs61829341	197599552	6	rs9442694	72391018
3	rs56156188	52730699	1	rs12127378	197601092	6	rs16881710	72391525
3	rs139683420	52730946	1	rs12041661	197601668	6	rs175360	72392227
3	rs2164885	52731269	1	rs17565558	197604346	6	rs1552418	72392503
3	rs11130317	52731483	1	rs10442656	197606708	6	rs74956132	72392699
3	rs13081155	52732429	1	rs114282645	197607459	6	rs79385039	72392941
3	rs3075809	52733082	1	rs6682968	197608612	6	rs9442695	72395398
3	rs6765687	52733106	1	rs16841842	197611682	6	rs1321834	72397798
3	rs8906	52739520	1	rs11331300	197612110	6	rs1358853	72398900
3	rs6617	52740182	1	rs10922269	197612247	6	rs6453563	72400667
3	rs79633493	52741032	1	rs2477070	197612759	6	rs9342867	72401653
3	rs3755798	52741160	1	rs1747826	197612994	6	rs2754043	72401810
3	rs34017441	52742413	1	rs6688289	197615058	6	rs78227980	72402864
3	rs115738369	52743289	1	rs1775448	197615130	6	rs2754044	72403927
3	rs34182518	52743854	1	rs114848122	197615193	6	rs6908543	72404002
3	rs13079063	52744460	1	rs1747827	197615391	6	rs11754467	72404171
3	rs11235	52745087	1	rs34496840	197615669	6	rs6909301	72404212
3	rs145014080	52747486	1	rs10494761	197617436	6	rs55730421	72404585
3	rs2878726	52748271	1	rs115195008	197618445	6	rs2840980	72404963
3	rs11716747	52748857	1	rs2488390	197619068	6	rs78464929	72405000
3	rs35526119	52749334	1	rs16841851	197621237	6	rs1321835	72405583
3	rs60770874	52750199	1	rs2477073	197622528	6	rs35506119	72406224
3	rs116493126	52750325	1	rs12746503	197625313	6	rs4707930	72406319
3	rs7624716	52751534	1	rs2253257	197626708	6	rs57810898	72408659
3	rs115745575	52753308	1	rs2477074	197626823	6	rs4415130	72408820
3	rs56196917	52756045	1	rs1775453	197629342	6	rs10942965	72409662
3	rs72960240	52759133	1	rs2488389	197631141	6	rs66666960	72412463
3	rs13063138	52759254	1	rs79599941	197631657	6	rs2754047	72413070
3	rs13087538	52763453	1	rs1775470	197632910	6	rs79864653	72413517
3	rs6769720	52764350	1	rs1775451	197635605	6	rs12526311	72413544
3	rs10780035	52764624	1	rs12117045	197638716	6	rs2754049	72414808
3	rs73078847	52764787	1	rs2488409	197644581	6	rs2145546	72415931
3	rs2268027	52766606	1	rs9633354	197644773	6	rs9360484	72415949
3	rs767418	52767427	1	rs36077246	197645403	6	rs2754050	72416458
3	rs1231194	52769047	1	rs2224873	197645449	6	rs12528636	72418076
3	rs11130323	52770277	1	rs12751508	197646544	6	rs73527251	72418122
3	rs56276589	52772198	1	rs10801621	197649941	6	rs2754051	72418574
3	rs34184137	52774017	1	rs2488410	197650505	6	rs71558905	72419055
3	rs75321479	52778210	1	rs1775450	197652602	6	rs9446449	72420588
3	rs2268026	52778347	1	rs1747811	197653036	6	rs927276	72421550
3	rs60058353	52778409	1	rs78823478	197653854	6	rs75887902	72422228
3	rs2072390	52780509	1	rs2488411	197658799	6	rs149667650	72422422
3	rs6762788	52792311	1	rs6691216	197659263	6	rs911364	72422458
3	rs1029871	52797634	1	rs1891497	197659555	6	rs1204325	72422694
3	rs72960281	52802871	1	rs2477076	197660143	6	rs1204326	72422775
3	rs13071584	52804487	1	rs78290672	197661907	6	rs911363	72423098

3	rs998909	52805093	1	rs151183648	197662801	6	rs12055765	72423935
3	rs11717836	52807736	1	rs115075685	197663354	6	rs66707419	72425163
3	rs72960285	52807816	1	rs2358774	197664395	6	rs1204328	72427371
3	rs1014969	52808341	1	rs114979698	197665307	6	rs1547683	72428824
3	rs3755799	52809193	1	rs12131588	197666111	6	rs1547684	72428869
3	rs2019065	52809525	1	rs2477077	197671115	6	rs80232328	72430245
3	rs62255400	52812231	1	rs16841894	197671853	6	rs829463	72430502
3	rs75097760	52814643	1	rs114780135	197674188	6	rs9358971	72430922
3	rs36012032	52814709	1	rs12354257	197674344	6	rs829464	72430997
3	rs2710323	52815905	1	rs80088735	197675371	6	rs829465	72431453
3	rs187624109	52816588	1	rs1573099	197683590	6	rs75470097	72431884
3	rs2710322	52817593	1	rs61829344	197683734	6	rs829466	72431919
3	rs3774354	52817675	1	rs1573098	197683807	6	rs60502272	72435788
3	rs3774355	52817778	1	rs1621898	197684499	6	rs9442699	72436075
3	rs11715487	52818301	1	rs1775457	197685151	6	rs116852172	72436273
3	rs2239551	52818579	1	rs28709435	197685827	6	rs9293851	72437006
3	rs2268023	52819327	1	rs115549264	197687255	6	rs829470	72437128
3	rs2535630	52819883	1	rs2488386	197688956	6	rs829472	72437718
3	rs678	52820981	1	rs12092948	197689967	6	rs62407672	72439008
3	rs1042779	52821011	1	rs115193323	197692691	6	rs11965010	72440089
3	rs2286798	52821177	1	rs2147300	197693127	6	rs9342868	72440800
3	rs111477914	52821666	1	rs12031377	197693266	6	rs829473	72440951
3	rs68094128	52821993	1	rs1573100	197693782	6	rs1328704	72453473
3	rs2239550	52822509	1	rs2488387	197694037	6	rs6915754	72456632
3	rs2300149	52822921	1	rs115893483	197695451	6	rs10942983	72548959
3	rs2239549	52823126	1	rs116252612	197697093	6	rs7771873	72562041
3	rs4687550	52823430	1	rs10754228	197697626	6	rs1147521	72586024
3	rs4687551	52823448	1	rs1747815	197698103	6	rs2040055	72603664
3	rs2270197	52824095	1	rs1747814	197699699	6	rs1111356	72647508
3	rs1076425	52825462	1	rs2488397	197701279	6	rs9358986	72649870
3	rs1075653	52825528	1	rs2488398	197701376	6	rs519470	72727772
3	rs9324	52825585	1	rs6428416	197701669	6	rs557365	72729528
3	rs7549	52825912	1	rs16841904	197701992	6	rs17757107	98472940
3	rs2071506	52826276	1	rs2488400	197702225	6	rs4839936	98489654
3	rs746694	52826620	1	rs2488401	197702401	6	rs13194250	98494218
3	rs2071507	52826707	1	rs16841912	197702476	6	rs901630	98539519
3	rs2071508	52826846	1	rs17641481	197703895	6	rs11964122	98546394
3	rs4687654	52827566	1	rs17641524	197704717	6	rs9375195	98562720
3	rs2239699	52827915	1	rs1747818	197708342	6	rs4144176	98699052
3	rs9881468	52828628	1	rs74541273	197708877	6	rs6932108	98713992
3	rs56009954	52829607	1	rs76312929	197709961	6	rs12205572	166779464
3	rs2240921	52830764	1	rs12066841	197710246	6	rs11557064	166780349
3	rs2240920	52831009	1	rs1747817	197711492	6	rs1546854	166796433
3	rs62255413	52831386	1	rs79279254	197717327	6	rs9348131	166850798
3	rs2240919	52831701	1	rs115710466	197719607	6	rs2281053	166860996
3	rs2535629	52833219	1	rs2488393	197722240	6	rs6932660	166862431
3	rs3617	52833805	1	rs75581554	197724011	6	rs9355581	166865273
3	rs9848554	52836337	1	rs10922273	197725373	6	rs4710051	166865675

3	rs17331151	52844534	1	rs1775454	197727298	6	rs12208871	166873823
3	rs4687657	52852538	1	rs114356669	197727327	6	rs3817792	166876628
3	rs2239547	52855229	1	rs77590973	197727599	6	rs6905447	166878799
3	rs2276816	52860816	1	rs1998598	197727642	6	rs9348138	166882847
3	rs2276817	52860936	1	rs61829349	197729197	6	rs9459678	166883981
3	rs4687554	52864135	1	rs114849732	197730112	6	rs6926393	166884211
3	rs13083728	52868445	1	rs17641842	197730559	6	rs743977	166936612
3	rs1573815	52870132	1	rs17567921	197730729	6	rs11755127	166952981
3	rs1139106	52871101	1	rs1775456	197733055	6	rs3799619	166976374
3	rs3733047	52871929	1	rs1342696	197733453	6	rs4710072	166998662
3	rs6445539	52874296	1	rs1539413	197733942	6	rs12528284	167013993
3	rs11720228	52877270	1	rs115602286	197734118	6	rs3799570	167030620
3	rs13080929	52885112	1	rs7516481	197734899	6	rs4145432	167043542
3	rs13059603	63827381	1	rs2488394	197735497	6	rs9356500	167056898
3	rs17069612	63977676	1	rs6661330	197735597	6	rs12213805	167107649
3	rs9848926	135816302	1	rs1924517	197738325	6	rs7770200	167151463
3	rs10512987	135823077	1	rs1924518	197738327	6	rs9295368	167166933
3	rs10935177	135851077	1	rs2488395	197738780	6	rs661325	167181032
3	rs1393786	135854035	1	rs1775444	197740690	6	rs2178806	167218762
3	rs17198295	135856318	1	rs4915561	197740938	6	rs6902718	167232907
3	rs6786582	135898984	1	rs2488396	197742171	7	rs2189634	11626988
3	rs645040	135926622	1	rs6685897	197742948	7	rs2526105	11657211
3	rs687339	135932359	1	rs78424313	197743274	7	rs10499416	11735354
3	rs9844666	135974216	1	rs1539414	197743506	7	rs2883585	11740020
3	rs1153877	136004925	1	rs12135210	197745243	7	rs9785045	11742920
3	rs1279840	136006576	1	rs1775447	197746486	7	rs12671899	11779082
3	rs9848883	136008432	1	rs12081207	197746565	7	rs10499405	11791774
3	rs556788	136027549	1	rs1747812	197746925	7	rs1526518	11809623
3	rs9853387	136038988	1	rs1775446	197746995	7	rs4721012	11825210
3	rs667920	136069472	1	rs6675292	197747067	7	rs6947930	11855636
3	rs9860801	136088038	1	rs1342694	197749868	7	rs2355102	11857720
3	rs9880211	136107549	1	rs1775469	197753074	7	rs1961842	11932891
3	rs6768743	136205007	1	rs1747825	197753136	7	rs17165338	11938060
3	rs9858105	136219264	1	rs1775468	197754064	7	rs4275121	11955266
3	rs9880099	136255776	1	rs1775467	197754947	7	rs6963121	11959035
3	rs7621025	136272246	1	rs1775466	197754967	7	rs10224784	11964376
3	rs12635723	136300732	1	rs1747823	197755646	7	rs10254316	11964570
3	rs12630999	136300927	1	rs76250973	197756099	7	rs10228842	11965352
3	rs13071220	136314119	1	rs1747822	197756223	7	rs17577569	12007154
3	rs9820513	136346292	1	rs1747821	197756433	7	rs6959385	12031826
3	rs4038586	136363291	1	rs1747820	197756464	7	rs2192735	12033933
3	rs6789152	136401351	1	rs2497863	197756577	7	rs10280298	12049985
3	rs13072691	136425600	1	rs1627999	197757072	7	rs11982169	12051898
3	rs13075615	136505832	1	rs111899896	197757144	7	rs2966619	12052059
3	rs6439664	136537074	1	rs1622740	197757736	7	rs17165507	12053458
3	rs1052620	136574521	1	rs3900909	197757846	7	rs11977758	12065305
3	rs13086336	180620256	1	rs1747828	197758154	7	rs2908738	12065405
3	rs1805579	180630565	1	rs1775462	197758223	7	rs17581371	12090560

3	rs1805584	180632030	1	rs1775461	197758388	7	rs17581713	12105982
3	rs1805564	180633485	1	rs1775460	197758439	7	rs4721023	12112471
3	rs1805567	180639252	1	rs1775459	197758569	7	rs10257135	104893565
3	rs1805571	180646631	1	rs1775458	197758574	7	rs2079341	104904235
3	rs1805572	180647263	1	rs2649557	197758895	7	rs12155351	104943302
3	rs1805589	180647410	1	rs2488402	197759335	7	rs4730087	105090115
3	rs1805604	180673936	1	rs77849192	197766026	7	rs4493843	105094283
3	rs2601	180700150	1	rs1578719	197766564	7	rs13437626	105096661
3	rs9810292	180714307	1	rs116788502	197766632	7	rs61741425	105148593
3	rs10470517	180726056	1	rs71633808	197767035	7	rs10271614	105208492
3	rs10049181	180736253	1	rs12749423	197767682	7	rs17280048	105277366
3	rs12635178	180797921	1	rs34451672	197767842	7	rs17345800	105277793
3	rs13072358	180873415	1	rs55873039	197767978	7	rs17280230	105288867
3	rs1878874	181035370	1	rs12564235	197768095	7	rs562221	105291444
3	rs6782970	181101544	1	rs12733235	197768282	7	rs10487888	140499107
3	rs4854914	181122223	1	rs1499593	197768507	7	rs9640168	140532269
3	rs4855026	181126962	1	rs12740849	197769821	7	rs9648716	140612163
3	rs1871355	181146051	1	rs6658157	197775931	7	rs11762469	140614212
3	rs9839993	181153038	1	rs4915564	197777147	7	rs4726135	140668619
3	rs1351235	181166485	1	rs12125742	197780744	7	rs38723	140695812
3	rs4855028	181168159	1	rs112562413	197780795	7	rs11764061	140754785
3	rs7373337	181183727	1	rs12123169	197780966	7	rs722219	140788213
3	rs13061117	181186466	1	rs12134279	197781198	7	rs11762357	140801429
3	rs7611361	181195182	1	rs116332466	197782332	7	rs1860752	140804805
3	rs1479176	181201428	1	rs4915566	197782736	7	rs2018320	140810892
4	rs364553	103149704	1	rs7538522	197782984	7	rs10246772	140914741
4	rs62322251	103149809	1	rs7526770	197783075	10	rs10761461	61883364
4	rs55980430	103151238	1	rs4915567	197784249	10	rs12761450	61885892
4	rs17032185	103151453	1	rs2358488	197784342	10	rs12355908	61937004
4	rs463661	103151556	1	rs12121863	197785420	10	rs16914591	61946060
4	rs460610	103154130	1	rs6428417	197787719	10	rs4245586	61950427
4	rs406749	103154313	1	rs12125942	197788562	10	rs74156431	61952950
4	rs456734	103154686	1	rs10922294	197790943	10	rs74156432	61953034
4	rs35166705	103156923	1	rs16841984	197793482	10	rs73263129	61954035
4	rs55866413	103156971	1	rs6704186	197793687	10	rs4310561	61954476
4	rs113361187	103160829	1	rs75622950	197793998	10	rs10994248	61954581
4	rs10433893	103160885	1	rs6667560	197794699	10	rs10509127	61954702
4	rs60245306	103163926	1	rs4915569	197795018	10	rs4948256	61956912
4	rs458779	103163959	1	rs35162839	197806129	10	rs6479698	61956999
4	rs459331	103167316	1	rs1010128	197806474	10	rs10821699	61957168
4	rs56385550	103167394	1	rs17642632	197808778	10	rs3897459	61957641
4	rs49178	103167613	1	rs10737695	197810094	10	rs78173462	61958545
4	rs457490	103168050	1	rs12118454	197810649	10	rs12266845	61958620
4	rs76407418	103168210	1	rs904576	197810847	10	rs16914616	61958983
4	rs181765414	103168356	1	rs6678151	197811282	10	rs10821700	61959061
4	rs11736058	103168673	1	rs10922298	197811566	10	rs16914619	61960135
4	rs28718345	103169827	1	rs10922299	197812165	10	rs77392577	61960201
4	rs657521	103170824	1	rs12120352	197812259	10	rs7081073	61960705

4	rs75671166	103172184	1	rs6692930	197812669	10	rs10821701	61961162
4	rs465144	103172201	1	rs2221134	197812732	10	rs10994250	61961795
4	rs62327889	103172826	1	rs12408939	197812823	10	rs78025156	61962177
4	rs460026	103173058	1	rs2201956	197812879	10	rs7078609	61963063
4	rs463373	103173173	1	rs12026183	197813032	10	rs2393623	61963427
4	rs444943	103173564	1	rs12141007	197813558	10	rs6479700	61963759
4	rs13126885	103173749	1	rs12740041	197814607	10	rs10821702	61964409
4	rs72924805	103173900	1	rs34478776	197814623	10	rs7923097	61964546
4	rs190178	103174015	1	rs10922300	197814685	10	rs10994253	61964752
4	rs151370	103174491	1	rs10801625	197814759	10	rs7895510	61964948
4	rs17032286	103174653	1	rs12403425	197814823	10	rs10740014	61965009
4	rs192068	103174819	1	rs10922301	197815016	10	rs10733760	61965078
4	rs149268	103175292	1	rs10922302	197815091	10	rs10732408	61965188
4	rs62327912	103176039	1	rs6428419	197815963	10	rs12358993	61965215
4	rs17823175	103176308	1	rs10922303	197816696	10	rs12411380	61965316
4	rs58379650	103176740	1	rs114156678	197816763	10	rs2297979	61965625
4	rs2298752	103177071	1	rs12043482	197817740	10	rs17805636	61966368
4	rs17032321	103177480	1	rs1004479	197817880	10	rs4948257	61966441
4	rs115980427	103179168	1	rs1553172	197818233	10	rs117118083	61966630
4	rs4476601	103179173	1	rs10922304	197820945	10	rs4948398	61966755
4	rs170871	103180149	1	rs1039288	197821607	10	rs10761465	61969227
4	rs189215	103180875	1	rs10922305	197821769	10	rs10740015	61969445
4	rs17032357	103182616	1	rs1039287	197821871	10	rs1954134	61969598
4	rs4698843	103182636	1	rs12145078	197822977	10	rs10761466	61969768
4	rs17032332	103182661	1	rs12145083	197822994	10	rs75489412	61970546
4	rs151390	103183510	1	rs6688420	197823052	10	rs7074185	61971486
4	rs9705	103183531	1	rs10801626	197823154	10	rs75918166	61971911
4	rs151392	103183935	1	rs10922306	197823360	10	rs7903287	61972114
4	rs151393	103184089	1	rs4915250	197826253	10	rs75831230	61972855
4	rs56072709	103186222	1	rs6704237	197826258	10	rs11819367	61973497
4	rs151395	103186708	1	rs4915251	197826672	10	rs10821703	61973814
4	rs151396	103186782	1	rs4915572	197826746	10	rs1417381	61981219
4	rs62327916	103187146	1	rs115545114	197827855	10	rs117337450	61981287
4	rs56215225	103187430	1	rs116582102	197828609	10	rs10994257	61982048
4	rs151399	103187562	1	rs10922307	197829619	10	rs74922600	61982657
4	rs151400	103187612	1	rs10922308	197829620	10	rs4133130	61984097
4	rs116767419	103188478	1	rs10922309	197829803	10	rs10994259	61984604
4	rs13107325	103188709	1	rs10922311	197829948	10	rs16914633	61985276
4	rs17823966	103189036	1	rs16842055	197830009	10	rs3927694	61985628
4	rs13114343	103189416	1	rs2047540	197830485	10	rs6479705	61988375
4	rs10489124	103189437	1	rs2047541	197830838	10	rs10821704	61988660
4	rs80176743	103189686	1	rs12062450	197832662	10	rs10740017	61989105
4	rs17225702	103189902	1	rs72744964	197832749	10	rs10761469	61989593
4	rs151401	103190128	1	rs10922312	197834773	10	rs10994265	61991066
4	rs17032400	103190229	1	rs10922313	197836347	10	rs10821705	61991969
4	rs151402	103190486	1	rs10922314	197837100	10	rs7903787	61993554
4	rs4699006	103190828	1	rs72744965	197837567	10	rs7908312	61997885
4	rs113926309	103191137	1	rs1499601	197838452	10	rs10821707	61997956

4	rs151403	103191447	1	rs116785330	197839402	10	rs1340654	61998060
4	rs111057652	103192243	1	rs1499602	197843204	10	rs4369348	61999321
4	rs10049581	103192893	1	rs75850339	197844253	10	rs34117040	61999333
4	rs11723499	170378624	1	rs10801629	197844367	10	rs76109618	62001552
4	rs13120831	170562598	1	rs74137403	197846588	10	rs140226558	62001875
4	rs17627781	170564316	1	rs76411361	197855964	10	rs7908011	62003389
5	rs10462087	45295280	1	rs10801632	197856163	10	rs12253367	62003462
5	rs34635	60513501	1	rs115713347	197856593	10	rs7901677	62005201
5	rs159362	60522452	1	rs7541116	197856736	10	rs78272901	62006214
5	rs6870703	60571540	1	rs74137408	197857845	10	rs16914644	62006261
5	rs13154581	60594520	1	rs10922323	197862321	10	rs7086638	62007342
5	rs7717082	60769411	1	rs10754235	197862598	10	rs7073041	62008145
5	rs13186194	60795485	1	rs61829103	197862946	10	rs6479706	62008708
5	rs6878927	60796291	1	rs9662705	197863414	10	rs10994278	62009219
5	rs11747755	60833841	1	rs12071207	197865112	10	rs10994279	62009405
5	rs771439	88590947	1	rs12086740	197865879	10	rs72820470	62009435
5	rs16867545	88623737	1	rs16842085	197866243	10	rs16914651	62009518
5	rs379717	88640130	1	rs12138550	197866357	10	rs76667912	62009709
5	rs17507679	88644971	1	rs10922324	197867405	10	rs35162716	62010413
5	rs7736534	88726337	1	rs10922326	197867662	10	rs12767186	62010523
5	rs9293514	88736275	1	rs2062479	197868281	10	rs35098882	62010755
5	rs16867571	88743219	1	rs10922327	197870378	10	rs72820475	62011936
5	rs6892330	88746405	1	rs111778590	197870705	10	rs117653366	62012619
5	rs4618461	88774328	1	rs4565750	197871493	10	rs17806245	62013067
5	rs10514308	88780868	1	rs4539165	197872019	10	rs16914658	62013157
5	rs12652212	88808594	1	rs72744974	197874385	10	rs72820477	62013222
5	rs1015976	109036234	1	rs2270763	197874949	10	rs10821708	62013424
5	rs1370959	109039180	1	rs188118779	197876393	10	rs35749260	62013597
5	rs2301010	109132461	1	rs4915574	197876757	10	rs10994284	62014015
5	rs11242417	137599334	1	rs4147201	197877103	10	rs12413212	62014315
5	rs7726580	137608666	1	rs78582470	197877332	10	rs12413208	62014417
5	rs7716275	137631073	1	rs10754236	197877488	10	rs16914660	62014629
5	rs10072318	137646221	1	rs116514830	197877817	10	rs10994285	62015007
5	rs7715815	137660982	1	rs12087257	197878701	10	rs10761470	62015637
5	rs3756765	137673004	1	rs78202414	197879787	10	rs2018783	62015782
5	rs3734168	137685690	1	rs12567149	197880072	10	rs12358938	62015926
5	rs757647	137707315	1	rs11588265	197880773	10	rs74344109	62016156
5	rs4835784	137730794	1	rs114974650	197881257	10	rs7090893	62016923
5	rs11956240	137840293	1	rs41269901	197882114	10	rs16914663	62017320
5	rs3849046	137851192	1	rs1109965	197882690	10	rs6479707	62018535
5	rs256002	137895218	1	rs111694034	197883809	10	rs7093272	62019175
5	rs7443140	137924729	1	rs79476113	197885014	10	rs12413099	62019322
5	rs7732337	137931814	1	rs74922488	197885301	10	rs16914668	62019749
5	rs10900855	137937537	1	rs4147202	197885543	10	rs1026533	62020287
5	rs11745933	137940165	1	rs41299601	197887584	10	rs16914670	62020728
5	rs11746692	137946355	1	rs10494762	197887838	10	rs72820489	62021146
5	rs753280	140024042	1	rs112701297	197887940	10	rs16914671	62021593
5	rs778591	140025484	1	rs17582664	197887985	10	rs41283532	62021781

5	rs2286394	140048544	1	rs116019611	197888308	10	rs72820490	62022121
5	rs11954514	140056775	1	rs76202560	197888309	10	rs16914674	62022298
5	rs702396	140068108	1	rs80079233	197888698	10	rs16914677	62023034
5	rs801167	140081423	1	rs12142428	197890160	10	rs79524330	62024566
5	rs2878996	140120733	1	rs12122995	197890422	10	rs190109826	62024899
5	rs2563269	140136468	1	rs13376365	197890802	10	rs72820491	62026557
5	rs3733708	140181734	1	rs12029707	197892252	10	rs17232638	62026812
5	rs3822346	140187322	1	rs72744986	197892334	10	rs10761471	62026984
5	rs7702336	152137936	1	rs34100116	197892551	10	rs16914683	62027535
5	rs11952081	152184299	1	rs78107421	197892713	10	rs16914687	62027606
5	rs6866034	152198322	1	rs113221369	197892886	10	rs35305500	62027683
5	rs12659802	152274478	1	rs6699938	197893152	10	rs12412493	62027914
5	rs11745863	152276092	1	rs11577674	197894455	10	rs35175163	62028179
5	rs4314462	152310798	1	rs16829946	197895717	10	rs1459731	62028797
5	rs17489682	152312444	1	rs12046958	197896728	10	rs16914693	62029008
5	rs17492381	152449415	1	rs4915134	197897189	10	rs71495632	62029439
5	rs12658628	152485398	1	rs12750987	197897263	10	rs71495633	62029934
5	rs17578068	152489022	1	rs12568860	197897332	10	rs72820495	62030396
5	rs17501123	152518117	1	rs10801633	197897496	10	rs7894698	62030725
5	rs13176510	152548490	1	rs34095703	197897646	10	rs79920459	62031981
5	rs12152961	152566888	1	rs116396834	197897715	10	rs117232551	62032613
5	rs2615160	152589262	1	rs146091153	197898072	10	rs116945347	62033377
5	rs35058195	152660612	1	rs10801634	197900589	10	rs1380453	62034355
5	rs12522290	152797656	1	rs80337194	197901727	10	rs72820501	62034492
5	rs17629195	153676502	1	rs77192411	197902045	10	rs1380452	62034497
5	rs6890923	153688205	1	rs6667492	197902814	10	rs72820502	62034855
6	rs13210258	28308671	1	rs1499598	197903352	10	rs72822204	62035182
6	rs9468344	28309569	1	rs74927474	197904639	10	rs10740018	62035369
6	rs6912584	28309590	1	rs7511852	197905132	10	rs7901951	62035501
6	rs12180820	28316478	1	rs2062482	197906223	10	rs7917111	62035619
6	rs1474589	28321544	1	rs10754237	197906399	10	rs17807007	62036121
6	rs213237	28323938	1	rs6660393	197906821	10	rs7098008	62036600
6	rs6921919	28325201	1	rs12065018	197907201	10	rs7068507	62037175
6	rs6922111	28325308	1	rs6428425	197907335	10	rs7068443	62037334
6	rs213230	28330264	1	rs34780702	197907554	10	rs12783491	62037607
6	rs213228	28331252	1	rs76727189	197909395	10	rs17807133	62038941
6	rs12661831	28337769	1	rs16842147	197910120	10	rs72822210	62039015
6	rs7773051	28340316	1	rs16842152	197910338	10	rs7910984	62039577
6	rs7774981	28346910	1	rs114920504	197910455	10	rs1380451	62039654
6	rs1052215	28348158	1	rs16842154	197910646	10	rs7911285	62039787
6	rs2531825	28349264	1	rs4915135	197910815	10	rs7900123	62040133
6	rs13213152	28349698	1	rs4915136	197910860	10	rs6479709	62040457
6	rs3734563	28349725	1	rs17644335	197912090	10	rs6479710	62040531
6	rs3799499	28354250	1	rs10922332	197912200	10	rs17233373	62040699
6	rs7764722	28354533	1	rs6671069	197913101	10	rs922118	62041129
6	rs9461458	28355875	1	rs75638927	197913574	10	rs922117	62041296
6	rs1361385	28358320	1	rs78941389	197913768	10	rs11592290	62041348
6	rs2232434	28358893	1	rs111868108	197914177	10	rs922116	62041469

6	rs2041230	28365515	1	rs4915255	197914298	10	rs10821709	62041591
6	rs2232423	28366151	1	rs73083840	197915230	10	rs10821710	62041683
6	rs2232422	28366262	1	rs12727777	197915297	10	rs11815139	62041733
6	rs4357130	28367683	1	rs78222204	197915363	10	rs12413770	62041860
6	rs2232419	28367768	1	rs17644490	197916347	10	rs12780624	62042160
6	rs9468370	28368940	1	rs75850678	197916391	10	rs12781705	62042207
6	rs13196606	28370078	1	rs61829143	197916580	10	rs10994292	62042321
6	rs6907950	28370246	1	rs115726423	197916864	10	rs12782314	62042464
6	rs6908137	28370393	1	rs17644551	197918402	10	rs12781443	62042627
6	rs4254981	28371402	1	rs10922333	197918487	10	rs12782806	62042711
6	rs1124131	28380248	1	rs10754238	197919593	10	rs12769536	62044194
6	rs6899389	28381140	1	rs1909507	197919693	10	rs12770090	62044341
6	rs1558205	28382262	1	rs116245320	197920994	10	rs7076645	62044411
6	rs6922169	28382932	1	rs115328872	197921246	10	rs72822218	62044526
6	rs10484540	28387443	1	rs2047539	197922395	10	rs68143724	62044939
6	rs16894091	28390137	1	rs10922335	197924313	10	rs71495634	62045287
6	rs16894095	28390230	1	rs17583875	197924770	10	rs1856828	62045474
6	rs1015690	28390526	1	rs10922337	197925320	10	rs10994293	62045698
6	rs3800328	28390843	1	rs35355724	197925416	10	rs35044728	62045776
6	rs2859365	28391465	1	rs35202895	197925500	10	rs75728162	62045878
6	rs1339898	28395506	1	rs10922338	197926219	10	rs10821711	62045932
6	rs7740351	28399412	1	rs116259466	197926427	10	rs75817524	62046071
6	rs16894106	28400339	1	rs4319370	197927281	10	rs56228657	62046696
6	rs7766356	28400538	1	rs2358777	197927351	10	rs12765897	62046981
6	rs11752919	28403603	1	rs77638754	197927517	10	rs898330	62047141
6	rs719370	28404353	1	rs2088651	197927580	10	rs898329	62047282
6	rs6917130	28406821	1	rs73067246	197927922	10	rs2893831	62047414
6	rs916403	28408258	1	rs61262489	197928019	10	rs898328	62047594
6	rs2531803	28411220	1	rs78639059	197928107	10	rs71495635	62048169
6	rs13190937	28411244	1	rs76367837	197928113	10	rs7095717	62048219
6	rs2531804	28411303	1	rs79951598	197928250	10	rs10821712	62048404
6	rs6902687	28413491	1	rs871874	197928523	10	rs10509128	62051027
6	rs13215804	28415572	1	rs2173568	197928569	10	rs74395695	62051471
6	rs6939966	28415885	1	rs871873	197928693	10	rs34874762	62052396
6	rs11755387	28416034	1	rs549890	197929497	10	rs79191744	62053005
6	rs6903535	28417222	1	rs80241185	197931243	10	rs34397303	62053692
6	rs2531809	28421368	1	rs10922339	197932391	10	rs34246330	62054315
6	rs2859369	28421567	1	rs72745000	197932974	10	rs1890951	62054706
6	rs1041926	28426296	1	rs72745001	197933628	10	rs7917654	62054746
6	rs2859374	28428145	1	rs12131587	197935734	10	rs10821713	62055781
6	rs9468379	28431184	1	rs11583952	197935915	10	rs7074145	62057445
6	rs2531815	28436060	1	rs413713	197937674	10	rs17233871	62058055
6	rs12663002	28441634	1	rs1233789	197938330	10	rs117318553	62058259
6	rs16894128	28444165	1	rs545711	197939323	10	rs1542462	62058396
6	rs1029328	28447915	1	rs2497866	197942485	10	rs11814450	62059268
6	rs1015811	28448086	2	rs820950	57738723	10	rs10994296	62060128
6	rs7770236	28450437	2	rs17809900	57781156	10	rs10761473	62060382
6	rs2859355	28461221	2	rs10204095	57799040	10	rs79186648	62061798

6	rs2859356	28465355	2	rs1527620	57803775	10	rs35412732	62061910
6	rs12661782	28466442	2	rs12621120	57806497	10	rs35628300	62062235
6	rs2071966	28473325	2	rs17049010	57849676	10	rs143228825	62062601
6	rs722788	28483150	2	rs2612313	57872702	10	rs10740022	62062686
6	rs406113	28483482	2	rs10199564	57897886	10	rs12770309	62063636
6	rs11757000	28484869	2	rs10496076	57942987	10	rs34517433	62063862
6	rs2078035	28488034	2	rs17228035	57943078	10	rs79665096	62064522
6	rs11757235	28489279	2	rs1460255	57978858	10	rs12217983	62064858
6	rs445870	28494327	2	rs11682175	57987593	10	rs10821714	62065131
6	rs2064424	28495962	2	rs1106090	58068741	10	rs118182049	62065307
6	rs380879	28500449	2	rs10167352	58099420	10	rs10443926	62065993
6	rs448450	28501006	2	rs118197020	58130326	10	rs77696714	62066817
6	rs451774	28502550	2	rs2717001	58137618	10	rs997238	62067448
6	rs13215054	28502794	2	rs2717002	58142647	10	rs10761476	62068818
6	rs392230	28504639	2	rs2678880	58158283	10	rs1551685	62069346
6	rs2394102	28512017	2	rs2717023	58161355	10	rs9645495	62070650
6	rs393414	28521316	2	rs2717024	58161397	10	rs9299440	62070695
6	rs6456825	28522695	2	rs2678889	58164223	10	rs10821716	62070753
6	rs414745	28526655	2	rs2717031	58166468	10	rs9645496	62070777
6	rs12110753	28529994	2	rs2717040	58169494	10	rs10509129	62071041
6	rs4711174	28532960	2	rs1568253	58170004	10	rs10821717	62072030
6	rs418092	28533946	2	rs1518395	58208074	10	rs117579984	62072102
6	rs450630	28542424	2	rs6732310	58210239	10	rs71495636	62072491
6	rs370520	28542520	2	rs2312147	58222928	10	rs10761477	62072704
6	rs17336532	28543264	2	rs11692441	156950640	10	rs10761480	62072740
6	rs16901848	28546952	2	rs2001859	156973115	10	rs1471246	62074139
6	rs7738979	28557312	2	rs1519805	156981694	10	rs1471247	62074280
6	rs6901724	28565827	2	rs2049582	157030835	10	rs7087252	62074676
6	rs418914	28568528	2	rs2037341	157032115	10	rs78254522	62074720
6	rs442439	28573006	2	rs16840135	157126321	10	rs187202361	62075176
6	rs911178	28574415	2	rs707124	157133013	10	rs10821718	62075547
6	rs9501180	28579471	2	rs298247	157233542	10	rs7079856	62075859
6	rs16894171	28602272	2	rs298259	157324825	10	rs34098396	62075983
6	rs7743296	28614610	2	rs297581	157352418	10	rs74807157	62076002
6	rs9295775	28627469	3	rs3852012	44045981	10	rs10761481	62076836
6	rs13194504	28630691	3	rs2372580	44049945	10	rs1340652	62078203
6	rs7767008	28630793	3	rs11718455	44056898	10	rs1340653	62078300
6	rs4713175	28635592	3	rs17076342	44079629	10	rs7090125	62078350
6	rs16894191	28641689	3	rs9871447	44079944	10	rs61847721	62079905
6	rs3864294	28641957	3	rs9311350	44132988	10	rs7899737	62080698
6	rs9393921	28645170	3	rs6800118	44141157	10	rs10821723	62082737
6	rs16894197	28648271	3	rs6796191	44167389	10	rs1350632	62082738
6	rs9885928	28650353	3	rs7626631	44197258	10	rs11593403	62083210
6	rs1319075	28662521	3	rs1488637	44332176	10	rs34314912	62083421
6	rs7382112	28665109	3	rs9819635	44425991	10	rs1459730	62084405
6	rs12111360	28667448	3	rs9855152	44458730	10	rs10761482	62085337
6	rs6939576	28669315	3	rs9857883	157887272	10	rs78776747	62086339
6	rs6908726	28671343	3	rs2693540	158001555	10	rs75448159	62086401

6	rs1107160	28671422	3	rs1210359	158007807	10	rs10740023	62088266
6	rs1107159	28671723	3	rs827168	158018849	10	rs149355724	62091722
6	rs9368570	28676193	3	rs12107103	158126282	10	rs12217173	62091912
6	rs1539584	28679945	3	rs4680433	158136848	10	rs7911934	62092090
6	rs9366725	28685981	3	rs1714509	158252958	10	rs12219340	62092193
6	rs6909960	28688921	3	rs7613824	158286152	10	rs4612751	62092917
6	rs9468413	28689672	3	rs6804230	158314188	10	rs117907293	62093601
6	rs1954407	28695149	3	rs1845138	158343907	10	rs7093702	62093709
6	rs9393929	28696063	4	rs7658646	41820275	10	rs991405	62094202
6	rs2893973	28697086	4	rs6840961	41827875	10	rs2199209	62094738
6	rs7755641	28699369	4	rs6447115	41841212	10	rs2199210	62094752
6	rs6456834	28700352	4	rs6447117	41846084	10	rs2219533	62094914
6	rs7766599	28703432	4	rs7655001	41882915	10	rs2219534	62094925
6	rs880638	28706912	4	rs17445354	41964337	10	rs972404	62095851
6	rs13211284	73157730	4	rs6447132	42134066	10	rs78038591	62096784
6	rs2781571	73170429	4	rs7667105	42147852	10	rs1380459	62097331
6	rs11756746	84286477	4	rs10034294	42187640	10	rs10994306	62097576
6	rs9362001	84322084	4	rs6852533	42195808	10	rs990685	62098149
7	rs7788921	1915282	4	rs6857570	42208939	10	rs10761483	62098398
7	rs10275045	1920826	4	rs9999533	42211367	10	rs10761484	62098458
7	rs10278591	1921362	4	rs7697417	42215425	10	rs56156031	62098699
7	rs10267593	1937261	4	rs7696007	42287348	10	rs56303906	62098775
7	rs6952727	1947958	5	rs17419291	87780432	10	rs10994308	62098952
7	rs12537914	1948359	5	rs16903182	87786580	10	rs10821727	62099113
7	rs4721184	1950784	5	rs16903184	87787332	10	rs10821729	62099173
7	rs4332037	1950809	5	rs16903232	87831734	10	rs10821730	62099271
7	rs11772205	1951236	5	rs992623	87840688	10	rs10821731	62099554
7	rs2056480	1954301	5	rs10078467	87877474	10	rs10821732	62099814
7	rs4721190	1954732	5	rs1501672	87963761	10	rs1903203	62100164
7	rs10268609	1962163	5	rs661311	88034867	10	rs117577974	62100612
7	rs12699477	1968953	5	rs3729703	88047617	10	rs10740024	62100840
7	rs2280550	1976556	5	rs622803	88069593	10	rs74775577	62101179
7	rs10237340	1987092	5	rs674747	88079010	10	rs79644767	62101236
7	rs1113737	1993078	5	rs244755	88095785	10	rs4442496	62101371
7	rs11773627	2002733	5	rs304151	88125853	10	rs4600155	62101381
7	rs12666575	2004421	5	rs11951031	88138731	10	rs4245587	62101589
7	rs3996329	2018871	5	rs7721449	103692385	10	rs4245588	62101746
7	rs4721295	2036669	5	rs1583953	103750804	10	rs1473404	62102316
7	rs1107592	2041432	5	rs1442112	103788551	10	rs2169081	62102389
7	rs7785626	2103005	5	rs2028527	103789492	10	rs10761485	62102660
7	rs6461115	2103668	5	rs6881764	103810310	10	rs2061487	62102842
7	rs3800879	2120378	5	rs4703034	103812072	10	rs2061488	62103166
7	rs1558462	2121196	5	rs10077445	103820340	10	rs2061489	62103196
7	rs10224497	2149967	5	rs10064760	103900475	10	rs4456215	62103249
7	rs10239050	2158390	5	rs768905	103909664	10	rs10821733	62103405
7	rs3800913	2163237	5	rs1363103	103917837	10	rs10821734	62103412
7	rs3800917	2167939	5	rs326236	103969568	10	rs2061490	62103662
7	rs3778991	2172455	5	rs33819	103980038	10	rs2061491	62103675

7	rs3778994	2175855	5	rs40465	103981726	10	rs2061492	62104016
7	rs4721441	2184060	5	rs6596581	103983318	10	rs10761486	62104303
7	rs3779003	2184902	5	rs325523	104045386	10	rs10761487	62104309
7	rs3800924	2188249	5	rs1370192	104069562	10	rs5009084	62104413
7	rs2529038	24620682	5	rs60271	104078233	10	rs4520549	62104516
7	rs2711091	24622086	5	rs17431165	104131657	10	rs4406773	62104574
7	rs2711130	24646774	5	rs7701167	104152321	10	rs4290174	62104583
7	rs6943306	24701995	5	rs12188289	104154736	10	rs4620673	62104704
7	rs1053962	24703298	5	rs10079325	104157752	10	rs4291629	62104758
7	rs2721777	24735746	5	rs6881692	124002868	10	rs4582919	62104776
7	rs2074142	24742585	5	rs12656846	124027136	10	rs4615975	62104917
7	rs10269819	24761117	5	rs12513661	124038251	10	rs4488150	62104918
7	rs2721794	24766507	5	rs11739274	124097421	10	rs10821735	62104980
7	rs2521760	24767483	5	rs4435859	124127720	10	rs55749838	62105048
7	rs886234	24769231	5	rs6882715	124129139	10	rs10821736	62105053
7	rs2237318	24769278	5	rs17152077	124157377	10	rs10994309	62105197
7	rs2237322	24770740	5	rs13166453	124186673	10	rs2061484	62105498
7	rs2521766	24772686	5	rs7716491	124237873	10	rs114427798	62105627
7	rs2237326	24797764	5	rs17254960	124284253	10	rs2061486	62105916
7	rs1468185	24818656	5	rs7707774	124288960	10	rs10761488	62105992
7	rs2237565	86455536	5	rs4240384	124291402	10	rs10994310	62106169
7	rs9655780	104667334	5	rs6875187	124302354	10	rs7096250	62106319
7	rs10268866	104676522	5	rs6864049	124330522	10	rs7096426	62106545
7	rs74959149	104752798	5	rs9327345	124339414	10	rs7097138	62106755
7	rs1142	104756326	5	rs12519279	124352162	10	rs113566024	62106814
7	rs1144	104756355	5	rs7711673	124389912	10	rs78930856	62106879
7	rs10257135	104893565	5	rs11741674	124443316	10	rs7084499	62106882
7	rs2079341	104904235	5	rs17515224	124458899	10	rs7084803	62107101
7	rs12155351	104943302	5	rs1550362	124480197	10	rs7100985	62107166
7	rs7783665	110039196	5	rs1550363	124480529	10	rs7085259	62107418
7	rs4730430	110047964	5	rs2963114	164314792	10	rs4948402	62107452
7	rs37713	110876320	5	rs2962865	164321412	10	rs4948403	62107662
7	rs2109298	110899634	5	rs17070336	164333983	10	rs1380458	62107690
7	rs11973097	110925758	5	rs4438895	164458965	10	rs10821737	62107802
7	rs37642	110977344	5	rs12233994	164522107	10	rs117704797	62108032
7	rs17443228	110986897	5	rs4535473	164561899	10	rs3919830	62108205
7	rs37667	111022481	5	rs13178865	164637211	10	rs10994311	62108380
7	rs4730488	111166165	5	rs1347153	164675241	10	rs4948405	62108419
7	rs320714	137058206	5	rs10076744	164682302	10	rs10761489	62108604
7	rs2278101	137080712	5	rs1432992	164749340	10	rs10761490	62108666
8	rs10503253	4180844	5	rs7356565	164755174	10	rs10761491	62108718
8	rs1504753	4181761	5	rs10045813	164762427	10	rs10761492	62108901
8	rs10503255	4185116	5	rs6555745	166851301	10	rs10761489	62108992
8	rs10866968	4188511	5	rs6887290	166866356	10	rs77914050	62109199
8	rs1875897	4188891	5	rs1030668	166876427	10	rs10733763	62109280
8	rs10104209	4189745	5	rs1864975	166887628	10	rs10821738	62109449
8	rs10091134	4190222	5	rs1025482	166893257	10	rs10821739	62109643
8	rs10105113	4190489	5	rs6886928	166984125	10	rs80244005	62109808

8	rs10107472	4190874	5	rs2243779	167084904	10	rs7089563	62109898
8	rs10108725	4191202	5	rs4432913	167098323	10	rs7073512	62109926
8	rs1316801	27429228	5	rs10072896	167169068	10	rs4948406	62110146
8	rs10101705	60746801	5	rs13436693	167170953	10	rs4948407	62110199
8	rs6471835	60746997	5	rs1862875	167182055	10	rs4948408	62110213
8	rs17322350	60747478	5	rs10516038	167189612	10	rs78075604	62110349
8	rs16925436	60839349	5	rs1364370	167196549	10	rs719095	62110949
8	rs17343814	60855552	5	rs918795	167219451	10	rs1417380	62111286
8	rs7843474	60870371	6	rs7767714	30488033	10	rs117614337	62112052
8	rs12677791	89342944	6	rs7768140	30488203	10	rs111318357	62112452
8	rs80039465	89343849	6	rs2534826	30495417	10	rs10740026	62112842
8	rs10504845	89345187	6	rs2534825	30495743	10	rs12412727	62113656
8	rs4395935	89348420	6	rs3094024	30495860	10	rs10821740	62113686
8	rs35175011	89352411	6	rs2534824	30496160	10	rs10761493	62113852
8	rs73275292	89365808	6	rs2534823	30496816	10	rs79161527	62115295
8	rs60298754	89373041	6	rs2516649	30496882	10	rs61846510	62115417
8	rs17730333	89376304	6	rs2524226	30497490	10	rs10821741	62115503
8	rs73277117	89381207	6	rs2534816	30498864	10	rs4948409	62115820
8	rs117758893	89382014	6	rs2534815	30499127	10	rs4948411	62115986
8	rs1504801	89383727	6	rs61123281	30499599	10	rs4948410	62116047
8	rs76034445	89388604	6	rs1362119	30499732	10	rs4948411	62116075
8	rs73277199	89395808	6	rs111960990	30501110	10	rs117787386	62116838
8	rs1012116	89397267	6	rs115484417	30501334	10	rs61846511	62117557
8	rs4369037	89397431	6	rs78000963	30502802	10	rs78880049	62117588
8	rs80287386	89416077	6	rs2516644	30503945	10	rs2393625	62119206
8	rs188295362	89416196	6	rs62407243	30505000	10	rs116946203	62120764
8	rs79676922	89438949	6	rs2524224	30505948	10	rs61846514	62121036
8	rs117646114	89475140	6	rs1468079	30506383	10	rs10509130	62121112
8	rs13280546	89477936	6	rs3130117	30508956	10	rs7083624	62121239
8	rs821109	89499611	6	rs2524222	30511170	10	rs7083556	62121387
8	rs58139040	89506295	6	rs1058318	30512163	10	rs7087958	62121891
8	rs716881	89516441	6	rs12192673	30512505	10	rs78309756	62122460
8	rs7826357	89542166	6	rs17195411	30512603	10	rs10994312	62122704
8	rs6990990	89548722	6	rs3815101	30514810	10	rs10994313	62122781
8	rs16884251	89555353	6	rs3130247	30515043	10	rs117443225	62122837
8	rs16884273	89558684	6	rs2844715	30516353	10	rs12411733	62123046
8	rs35385383	89566464	6	rs2516640	30516458	10	rs7916917	62123334
8	rs1352318	89566903	6	rs2844714	30518619	10	rs61846516	62123438
8	rs7819913	89568393	6	rs2844713	30519258	10	rs7917112	62123665
8	rs1580508	89570460	6	rs115134085	30519619	10	rs7906868	62124745
8	rs7814164	89573045	6	rs2074505	30521137	10	rs7894561	62125620
8	rs13263450	89574375	6	rs2516647	30526845	10	rs77560928	62125814
8	rs10504857	89582625	6	rs114055010	30527296	10	rs71495639	62126881
8	rs7838490	89585048	6	rs3888778	30529622	10	rs79359809	62127811
8	rs4484741	89605536	6	rs3888777	30529816	10	rs79663415	62127823
8	rs9969514	89605999	6	rs17189092	30529873	10	rs10821744	62127894
8	rs4269585	89608690	6	rs2074504	30530245	10	rs1981251	62127927
8	rs4534168	89609651	6	rs2074503	30530496	10	rs74563482	62128085

8	rs13272330	89615855	6	rs114423170	30531413	10	rs76446094	62128096
8	rs6990941	89644431	6	rs9262113	30532278	10	rs75362238	62128472
8	rs12334864	89646080	6	rs3130240	30536071	10	rs34939353	62128506
8	rs11995369	89649177	6	rs3130041	30536133	10	rs77938911	62128671
8	rs13257525	89677161	6	rs3130242	30537339	10	rs78743852	62128831
8	rs16886095	89713000	6	rs3132613	30537606	10	rs79131601	62129592
8	rs7833665	89721771	6	rs3130243	30537853	10	rs10994319	62130265
8	rs7829812	89722274	6	rs28360036	30538821	10	rs3851251	62130429
8	rs13262894	111532085	6	rs4148247	30539205	10	rs1824405	62130662
8	rs13274273	111572122	6	rs3132612	30539438	10	rs7072841	62131838
8	rs10098073	143309504	6	rs2269710	30539952	10	rs77917688	62131991
9	rs1319017	84736303	6	rs2269709	30540890	10	rs80215809	62132010
9	rs4442221	84750462	6	rs1264451	30541237	10	rs1459728	62132129
9	rs4877686	84765612	6	rs6927603	30541696	10	rs1380455	62132820
10	rs1570939	18685891	6	rs3132611	30541852	10	rs74707031	62132830
10	rs7909027	18695892	6	Chr6:30652032	30544053	10	rs1380454	62133051
10	rs11593522	18705120	6	rs3132610	30544401	10	rs78050293	62133183
10	rs11014171	18711195	6	rs732229	30544941	10	rs1010556	62133503
10	rs4747345	18712352	6	rs3132609	30544982	10	rs1010555	62133881
10	rs10741058	18716145	6	rs3130043	30545022	10	rs76976576	62133978
10	rs4548524	18716867	6	rs113473909	30545879	10	rs78638255	62134176
10	rs4145903	18718052	6	rs1264440	30551286	10	rs117134507	62135194
10	rs10828705	18735355	6	rs1264439	30552502	10	rs10994321	62135206
10	rs12243859	18740632	6	rs3130244	30552937	10	rs75396339	62135257
10	rs12219393	18755609	6	rs17195453	30553869	10	rs10821745	62136206
10	rs10828749	18756881	6	rs1264437	30553871	10	rs10994322	62136279
10	rs1409204	18763822	6	rs192021424	30554183	10	rs75342328	62136934
10	rs4919685	104587362	6	rs116294079	30560620	10	rs4147263	62137665
10	rs72841265	104587617	6	rs13195066	30560796	10	rs1459727	62138491
10	rs10883783	104591152	6	rs7749109	30561724	10	rs10821746	62140840
10	rs284849	104591182	6	rs1264432	30562021	10	rs72806126	62142611
10	rs17115100	104591393	6	rs7749978	30562041	10	rs7895653	62143348
10	rs45609333	104591639	6	rs6917434	30562647	10	rs72806129	62143522
10	rs284848	104592125	6	rs1264431	30562671	10	rs7896287	62143794
10	rs4919686	104592249	6	rs17189120	30563362	10	rs72806131	62144801
10	rs3740397	104592675	6	rs112559044	30563495	10	rs79475892	62145056
10	rs1004467	104594507	6	rs9262119	30563550	10	rs72806132	62145736
10	rs743575	104594906	6	rs6934466	30563608	10	rs10994324	62145937
10	rs4919687	104595248	6	rs3130245	30564343	10	rs117881056	62145992
10	rs3781287	104595420	6	rs1264429	30565101	10	rs117269095	62146003
10	rs3781286	104595719	6	rs7742754	30566154	10	rs72806133	62146403
10	rs3824755	104595849	6	rs6457246	30566254	10	rs4948412	62146576
10	rs45463800	104596356	6	rs3216457	30567843	10	rs4948413	62146640
10	rs10786712	104596396	6	rs7759666	30569489	10	rs117011548	62148121
10	rs6163	104596924	6	rs17189134	30570480	10	rs16914968	62149543
10	rs6162	104596981	6	rs17195474	30571197	10	rs77622274	62149864
10	rs743572	104597152	6	rs1264423	30571471	10	rs1551684	62150203
10	rs2486758	104597480	6	rs7742291	30573139	10	rs1551683	62150359

10	rs117856379	104599959	6	rs7742650	30573334	10	rs3808942	62150848
10	rs79932911	104601618	6	rs9262122	30573842	10	rs72806134	62150926
10	rs58109969	104602607	6	rs6457248	30573866	10	rs72806135	62150936
10	rs11191416	104604916	6	rs12174151	30574194	10	rs3808943	62151015
10	rs79424856	104605329	6	rs1264420	30575603	10	Chr10:61821276	62151270
10	rs10509762	104606472	6	rs1264419	30576781	10	rs7082025	62152022
10	rs72841269	104609654	6	rs60337024	30576801	10	rs113238321	62152415
10	rs7096475	104609676	6	rs6901251	30577593	10	rs10821747	62152721
10	rs4290163	104610926	6	rs2239514	30577792	10	rs76972414	62152944
10	rs17523050	104613945	6	rs2239515	30577966	10	rs77787726	62153164
10	rs3824754	104614350	6	rs2239516	30578048	10	rs77173454	62153253
10	rs4919690	104616500	6	rs2267637	30578335	10	rs4948416	62154169
10	rs4409766	104616663	6	rs2252745	30579315	10	rs72806140	62154684
10	rs76429823	104617647	6	rs7767002	30579417	10	rs7903441	62155308
10	rs7096249	104618524	6	rs6457249	30579556	10	rs12416380	62156154
10	rs7096169	104618695	6	rs17195516	30580085	10	rs7907761	62156169
10	rs10786715	104620735	6	rs2267639	30580637	10	rs117177424	62157655
10	rs41287472	104621829	6	rs2267640	30580893	10	rs79278901	62158107
10	rs9527	104623578	6	rs55777621	30584133	10	rs10994326	62158385
10	rs4917985	104624072	6	rs16867845	30585333	10	rs72806146	62159351
10	rs4919691	104624475	6	rs2302347	30587613	10	rs4948417	62161618
10	rs17115171	104624672	6	rs17189190	30589414	10	rs10994327	62162053
10	rs10786716	104625178	6	rs61109058	30590377	10	rs72806151	62162074
10	rs11191424	104625886	6	rs35554791	30591129	10	rs7087726	62162582
10	rs11191425	104625970	6	rs2270172	30592414	10	rs10994330	62163439
10	rs7098825	104628234	6	rs6917935	30593075	10	rs1599166	62163729
10	rs17115179	104628624	6	rs34315095	30593485	10	rs113984400	62164153
10	rs17882328	104628674	6	rs111850078	30594102	10	rs77782467	62164346
10	rs7085104	104628873	6	rs2517565	30594394	10	rs72806156	62164577
10	rs12416687	104629011	6	rs3814959	30594827	10	rs7909961	62165578
10	rs4917986	104630181	6	rs6904236	30596135	10	rs72806158	62165906
10	rs17878846	104630412	6	rs4713340	30599702	10	rs72806159	62166449
10	rs7920697	104633337	6	rs13201129	30601067	10	rs12776510	62167928
10	rs10509760	104634107	6	rs12665339	30601232	10	rs72806162	62168629
10	rs3740394	104634474	6	rs3094104	30609629	10	rs7099869	62170256
10	rs12764049	104634956	6	rs3132608	30609661	10	rs7100501	62170830
10	rs12765002	104635348	6	rs3132595	30610042	10	rs10994331	62171445
10	rs111450127	104635759	6	rs9262132	30611350	10	rs10994332	62171600
10	rs77335224	104636276	6	rs1140809	30611676	10	rs10761495	62171796
10	rs3740393	104636655	6	rs2269708	30611968	10	rs10821753	62171983
10	rs3740392	104636855	6	rs9262135	30618906	10	rs79277950	62172183
10	rs11191434	104637508	6	rs17281677	30622459	10	rs16914993	62172434
10	rs11191438	104637864	6	rs3094100	30625155	10	rs10994333	62173051
10	rs10786719	104637992	6	rs1076829	30627216	10	rs12416179	62173207
10	rs3740391	104638421	6	rs9262138	30627867	10	rs77543219	62174183
10	rs3740390	104638480	6	rs3130000	30628082	10	rs10994334	62174663
10	rs11191439	104638723	6	rs1109146	30629648	10	rs12764333	62175060
10	rs12774047	104639738	6	rs4713346	30631063	10	rs10994335	62175630

10	rs17115203	104639969	6	rs3094097	30633875	10	rs12256333	62175681
10	rs149053625	104640757	6	rs2285321	30637998	10	rs10821757	62176620
10	rs10883790	104640955	6	rs3094094	30639412	10	rs10821758	62176632
10	rs12249194	104643698	6	rs6937357	30640324	10	rs10821759	62176933
10	rs72841273	104647200	6	rs9468805	30643709	10	rs10761496	62177690
10	rs77505796	104647774	6	rs9262141	30644137	10	rs10761497	62177839
10	rs12768205	104647849	6	rs6457254	30649134	10	rs10821760	62178257
10	rs7085854	104650251	6	rs9262142	30650026	10	rs10994336	62179812
10	rs76255497	104651955	6	rs2394392	30650318	10	rs79576783	62180122
10	rs75691516	104652045	6	rs3129996	30651587	10	rs7092883	62180931
10	rs11191447	104652323	6	rs9262143	30652781	10	rs10994338	62181128
10	rs12763665	104653717	6	rs2269706	30652872	10	rs1934759	62181923
10	rs78561456	104653872	6	rs2269705	30656847	10	rs10994339	62182048
10	rs11191450	104654211	6	rs2269704	30656953	10	rs10821761	62182269
10	rs10883796	104655315	6	rs1075496	30658239	10	rs10821762	62182509
10	rs74233296	104655350	6	rs2269703	30658385	10	rs76861408	62183100
10	rs80327774	104656671	6	rs3130663	30666594	10	rs10761498	62183385
10	rs113282265	104657248	6	rs9468811	30666669	10	rs950826	62183837
10	rs11191453	104659852	6	rs2894043	30668488	10	rs12263856	62184500
10	rs11191454	104660004	6	rs9405048	30670292	10	rs12257109	62184600
10	rs10786722	104660068	6	rs3132589	30672433	10	rs7074460	62184843
10	rs10748835	104660256	6	rs10947087	30672463	10	rs4948418	62185494
10	rs41310292	104660942	6	rs2269702	30675135	10	rs10821763	62186118
10	rs17884001	104661245	6	rs6929626	30679333	10	rs10994342	62187607
10	rs1046778	104661484	6	rs3094093	30679628	10	rs3999537	62188935
10	rs11191457	104661708	6	rs2075015	30680608	10	rs3999538	62189165
10	rs7897654	104662458	6	rs9262152	30680916	10	rs77845949	62189495
10	rs10883799	104663310	6	rs6924270	30681980	10	rs10994344	62189852
10	rs56358609	104664289	6	rs3132587	30685066	10	rs4347329	62190508
10	rs12253284	104665145	6	rs4713354	30685420	10	rs117467096	62191981
10	rs12221193	104665267	6	rs3132585	30687614	10	rs4072837	62192060
10	rs12261040	104665397	6	rs3132584	30688427	10	rs4072838	62192151
10	rs77180047	104666757	6	rs25527	30690938	10	rs7896140	62192297
10	rs4532960	104667406	6	rs8233	30692965	10	rs4072839	62192301
10	rs10786724	104668942	6	rs9500864	30693232	10	rs55683151	62192515
10	rs72841283	104669442	6	rs3095330	30693633	10	rs3935092	62192672
10	rs10883800	104671212	6	rs3095329	30693816	10	rs12146283	62192746
10	rs12251035	104671459	6	rs3094127	30697447	10	rs75263098	62193882
10	rs75899148	104671966	6	rs9500866	30697523	10	rs61847579	62194149
10	rs7073295	104672233	6	rs9501028	30697561	10	rs117015738	62196867
10	rs12780220	104674104	6	rs9501029	30697740	10	rs12770716	62196872
10	rs6584532	104674516	6	rs1064627	30698541	10	rs57679969	62197886
10	rs192145925	104676954	6	rs3909184	30699384	10	rs10994349	62199279
10	rs117107046	104676968	6	rs9262155	30700497	10	rs78554060	62199681
10	rs12221064	104677126	6	rs9968884	30700695	10	rs10994350	62199825
10	rs75650764	104677629	6	rs2535320	30704986	10	rs79738223	62200746
10	rs74376228	104677662	6	rs3130660	30706361	10	rs4948420	62200882
10	rs77760630	104677690	6	rs1059612	30708955	10	rs10821766	62201684

10	rs10883801	104677887	6	rs3094125	30709357	10	rs77157280	62202718
10	rs2297786	104679978	6	rs10947088	30709830	10	rs76929953	62205261
10	rs2297787	104680137	6	rs10947089	30710135	10	rs61847646	62207387
10	rs10883802	104680362	6	rs17719	30711031	10	rs7922622	62207523
10	rs17115213	104681143	6	rs8512	30711357	10	rs7070102	62207943
10	rs10509758	104681710	6	rs2301277	30712327	10	rs117635305	62207984
10	rs11191464	104682148	6	rs3130662	30713808	10	rs10159663	62208417
10	rs10786725	104682357	6	rs2535319	30714479	10	rs4511241	62209255
10	rs78821730	104684544	6	rs4713358	30716117	10	rs6479713	62209555
10	rs111426607	104685108	6	rs12210092	30716719	10	rs75224225	62209646
10	rs12411886	104685299	6	rs9262164	30717389	10	rs10733764	62210345
10	rs17725614	104685493	6	rs2535324	30718035	10	rs1954117	62210588
10	rs34572965	104685537	6	rs2535323	30718180	10	rs7089604	62210846
10	rs140683362	104686884	6	rs12202428	30719909	10	rs3999542	62211158
10	rs72841290	104689236	6	rs28780111	30720311	10	rs7917190	62211528
10	rs10509759	104689665	6	rs3129973	30721143	10	rs61847647	62211542
10	rs12416441	104689990	6	rs3095328	30723781	10	rs10821767	62212557
10	rs7093461	104693479	6	rs2394401	30724430	10	rs7921710	62212664
10	rs10748836	104693917	6	rs3095326	30725841	10	rs117134049	62212796
10	rs17787717	104695699	6	rs3095341	30726748	10	rs7069341	62213391
10	rs3897401	104696379	6	rs3095340	30726939	10	rs7087691	62213791
10	rs5011520	104697516	6	rs3869075	30727282	10	rs56333342	62213967
10	rs181339164	104697553	6	rs3131036	30728290	10	rs4948421	62213993
10	rs12774555	104697707	6	rs3094122	30728360	10	rs4948260	62214490
10	rs12414777	104697781	6	rs11966040	30729487	10	rs10994357	62215071
10	rs10786727	104698523	6	rs6926530	30730267	10	rs77512729	62216092
10	rs17787824	104698571	6	rs3094121	30730960	10	rs76590127	62216935
10	rs4919694	104698978	6	rs9262176	30731330	10	rs79911589	62217219
10	rs77059182	104701251	6	rs13214831	30731505	10	rs75273753	62217235
10	rs72841298	104701872	6	rs3094120	30731556	10	rs10047260	62217805
10	rs77273695	104704530	6	rs3129975	30732054	10	rs10821768	62218305
10	rs111973848	104704736	6	rs13204228	30734255	10	rs10740028	62219990
10	rs61870830	104704762	6	rs12210947	30735105	10	rs1934750	62220244
10	rs3897402	104705412	6	rs11963870	30735229	10	rs10994359	62222107
10	rs10786728	104705706	6	rs12526186	30736151	10	rs12761294	62222236
10	rs10883804	104706520	6	rs3094117	30737486	10	rs10761503	62222414
10	rs11191472	104707016	6	rs9262182	30738042	10	rs75803729	62222908
10	rs78602818	104708030	6	rs3094116	30738408	10	rs4426089	62223112
10	rs10883805	104708251	6	rs3095336	30738446	10	rs79082892	62223225
10	rs12783467	104708312	6	rs6911628	30739846	10	rs4948422	62223362
10	rs11191473	104710710	6	rs6912077	30739904	10	rs117099644	62223852
10	rs72847279	104717669	6	rs3130666	30740160	10	rs12243786	62223944
10	rs12413409	104719096	6	rs16897888	30740515	10	rs7096617	62224554
10	rs76892505	104719290	6	rs4248148	30742134	10	rs117388564	62224744
10	rs10883808	104721126	6	rs17481190	30743014	10	rs114593644	62225456
10	rs78260931	104721962	6	rs3130667	30743241	10	rs4948261	62225539
10	rs7089061	104723015	6	rs11757629	30744529	10	rs10994362	62225792
10	rs7073179	104723091	6	rs3130673	30746519	10	rs1890927	62226261

10	rs11191479	104723620	6	rs12526481	30746633	10	rs10761504	62226848
10	rs75014273	104724573	6	rs9468829	30749233	10	rs1572843	62226980
10	rs12778673	104725170	6	rs9468830	30749712	10	rs1572842	62227395
10	rs72847281	104725471	6	rs7752959	30750682	10	rs72807911	62228288
10	rs10883810	104727224	6	rs9295912	30752184	10	rs10994364	62228327
10	rs7899622	104727333	6	rs4713360	30753146	10	rs10994365	62228499
10	rs2065977	104729415	6	rs13212094	30753455	10	rs10994366	62229789
10	rs11191485	104729816	6	rs12527415	30754540	10	rs143476356	62230224
10	rs74749600	104732996	6	rs13213720	30755320	10	rs4466779	62230864
10	rs1538204	104733893	6	rs28780116	30755668	10	rs10994367	62231295
10	rs11191487	104734623	6	rs16897899	30755744	10	rs77897278	62233139
10	rs12248123	104735366	6	rs28780119	30755863	10	rs117219308	62233407
10	rs7096452	104736563	6	rs16897900	30755893	10	rs79716236	62233491
10	rs7080462	104736699	6	rs13201769	30756066	10	rs1938534	62234012
10	rs7096269	104736721	6	rs13201901	30756140	10	rs185126186	62234070
10	rs72847291	104736855	6	rs4713365	30756183	10	rs1938533	62234109
10	rs12268849	104737682	6	rs4713366	30756361	10	rs17810349	62235173
10	rs10883815	104739179	6	rs6920124	30756593	10	rs7089985	62235234
10	rs11191489	104740568	6	rs6900029	30756596	10	rs7090243	62235443
10	rs12260436	104741114	6	rs6920649	30756883	10	rs10733765	62237399
10	rs55833108	104741583	6	rs10947095	30757575	10	rs10994369	62237531
10	rs11191490	104745174	6	rs3131043	30758466	10	rs10821769	62237835
10	rs3902934	104746649	6	rs17189441	30758555	10	rs17236977	62237923
10	rs11191491	104747373	6	rs3129981	30758857	10	rs12356018	62238682
10	rs11191492	104747534	6	rs3131050	30760025	10	rs10740029	62238902
10	rs75219158	104747594	6	rs3131051	30760181	10	rs116934723	62239498
10	rs1890184	104748459	6	rs7740525	30760698	10	rs7067886	62239503
10	rs1890185	104748718	6	rs3094112	30761734	10	rs7912663	62240587
10	rs75810873	104751570	6	rs3129985	30762542	10	rs11816019	62240823
10	rs10786731	104751781	6	rs3131060	30763291	10	rs12356634	62241548
10	rs10509757	104752960	6	rs3129986	30763562	10	rs12771347	62241727
10	rs12569364	104753507	6	rs6930444	30763632	10	rs12770054	62241757
10	rs11191494	104753675	6	rs3131061	30763656	10	rs10761505	62242145
10	rs61870841	104754813	6	rs3131063	30763756	10	rs72807927	62243039
10	rs10883817	104755431	6	rs3131064	30763893	10	rs7910492	62243104
10	rs7894588	104756030	6	rs3130641	30764081	10	rs10994371	62243199
10	rs7894959	104756182	6	rs12660860	30764241	10	rs10761507	62243232
10	rs7898770	104756636	6	rs12660883	30764420	10	rs10994372	62243551
10	rs12764154	104757709	6	rs28670020	30764838	10	rs17816232	62243554
10	rs35791366	104757753	6	rs1264377	30764907	10	rs17237427	62243649
10	rs7908450	104759191	6	rs1264376	30765579	10	rs74946632	62243711
10	rs10883818	104759586	6	rs10947096	30765895	10	rs12764668	62243866
10	rs78358813	104760980	6	rs12190167	30766194	10	rs12764533	62243916
10	rs12779991	104761930	6	rs3129987	30766204	10	rs12764726	62243997
10	rs10786732	104762535	6	rs12197154	30766244	10	rs7898622	62244379
10	rs10883819	104763308	6	rs6905957	30766740	10	rs78929840	62244738
10	rs34060850	104764065	6	rs4639381	30766765	10	rs10994375	62245124
10	rs77426338	104764148	6	rs4587208	30767013	10	rs12356720	62245219

10	rs11191499	104764271	6	rs4248149	30767627	10	rs118052890	62245593
10	rs10883820	104764661	6	rs2394403	30767869	10	rs11596715	62245834
10	rs67908413	104764989	6	rs4573120	30767947	10	rs11597195	62245963
10	rs11191502	104765494	6	rs4711232	30768412	10	rs10994376	62245981
10	rs12258551	104767892	6	rs4711233	30768426	10	rs111754174	62246400
10	rs11191506	104768634	6	rs4711235	30768636	10	rs144479748	62246568
10	rs7100592	104769098	6	rs9366759	30768955	10	rs10761508	62246634
10	rs11191511	104769709	6	rs1264373	30769273	10	rs78559636	62246834
10	rs6584536	104770702	6	rs3129988	30769478	10	rs10761509	62247057
10	rs111326718	104771211	6	rs1264372	30769726	10	rs7911959	62247330
10	rs7071373	104771922	6	rs13198118	30770732	10	rs1938530	62247877
10	rs11191512	104772699	6	rs4713372	30770778	10	rs56164449	62247878
10	rs11191513	104772984	6	rs9380192	30772344	10	rs1938529	62247952
10	rs11191514	104773364	6	rs3094123	30772378	10	rs11812654	62247963
10	rs12245343	104774050	6	rs4711240	30773239	10	rs1938528	62248141
10	rs7914558	104775908	6	rs4713376	30773314	10	rs117193770	62248749
10	rs77787671	104776205	6	rs13191728	30773349	10	rs11813629	62249495
10	rs10509763	104776391	6	rs3130783	30774357	10	rs10994378	62249501
10	rs10509764	104776475	6	rs9378109	30774474	10	rs76446519	62249556
10	rs11191515	104776527	6	rs1264362	30776590	10	rs11813688	62249777
10	rs6584537	104778267	6	rs12200562	30776621	10	rs73257065	62250087
10	rs58289149	104780552	6	rs1264361	30777498	10	rs117512390	62250178
10	rs78193706	104780564	6	rs9380197	30778203	10	rs73257066	62250285
10	rs72850111	104780879	6	rs9391696	30778786	10	rs73257067	62250485
10	rs72850112	104781301	6	rs4327730	30780936	10	rs117884097	62250522
10	rs72850116	104783829	6	rs886425	30781301	10	rs73257068	62250796
10	rs4917991	104783833	6	rs4286818	30781923	10	rs10821775	62250855
10	rs1538205	104784794	6	rs886424	30782002	10	rs77430401	62250910
10	rs79227132	104785737	6	rs2894046	30782105	10	rs76762467	62250984
10	rs12246739	104787019	6	rs886423	30782205	10	rs73257069	62251035
10	rs7087459	104790999	6	rs2394412	30782235	10	rs75827784	62251144
10	rs111990095	104791595	6	rs2394413	30782303	10	rs12256942	62251288
10	rs12779535	104791760	6	rs2394414	30782323	10	rs10994383	62251553
10	rs72850120	104791837	6	rs9295924	30782361	10	rs73257077	62252109
10	rs11191523	104792400	6	rs2894047	30782407	10	rs72406472	62252495
10	rs117521106	104792461	6	rs4713380	30785273	10	rs11813678	62252913
10	rs7088200	104793235	6	rs9380198	30785886	10	rs17816562	62252985
10	rs11191525	104793435	6	rs9348843	30786676	10	rs67801419	62253125
10	rs75970938	104793648	6	rs4713382	30787175	10	rs10994384	62253302
10	rs72850123	104793992	6	rs4713383	30787241	10	rs12246785	62253572
10	rs10786733	104794947	6	rs4713385	30787593	10	rs12246745	62253685
10	rs11191527	104795134	6	rs1264353	30787762	10	rs12763531	62254088
10	rs7920868	104795409	6	rs3094111	30788191	10	rs7897838	62254337
10	rs4633383	104795723	6	rs16897919	30788588	10	rs7897863	62254477
10	rs78893207	104796138	6	rs1264352	30789647	10	rs77724535	62254580
10	rs12415388	104796345	6	rs12195508	30789665	10	rs7898141	62254680
10	rs10883822	104796652	6	rs9262216	30789694	10	rs11816666	62254898
10	rs10218853	104796787	6	rs4713389	30790604	10	rs56122661	62255223

10	rs76099321	104797283	6	rs6901761	30790798	10	rs7077572	62255455
10	rs7073323	104797423	6	rs12203797	30790807	10	rs58354369	62255751
10	rs58317752	104799267	6	rs4713390	30791971	10	rs1938536	62256285
10	rs11191530	104800044	6	rs4713391	30792235	10	rs2211656	62256593
10	rs56376544	104800099	6	rs12192704	30792270	10	rs1934760	62256745
10	rs58700372	104800205	6	rs12206022	30792364	10	rs7915051	62257915
10	rs7092097	104801087	6	rs12192828	30792492	10	rs6479714	62258616
10	rs12255761	104801866	6	rs9394020	30792698	10	rs2893837	62259143
10	rs7089680	104802071	6	rs7751869	30793314	10	rs2393638	62259224
10	rs12257935	104803062	6	rs12212837	30793667	10	rs10821776	62260101
10	rs116954400	104806770	6	rs915664	30794617	10	rs10994386	62260544
10	rs12266291	104806884	6	rs3888583	30795008	10	rs75377991	62261535
10	rs12241091	104808997	6	rs7772131	30795171	10	rs143747491	62261782
10	rs12219027	104811203	6	rs3130784	30796106	10	rs55672059	62262242
10	rs4917994	104811699	6	rs9380199	30796271	10	rs17816795	62263522
10	rs118031532	104812799	6	rs1264350	30796545	10	rs10509134	62266146
10	rs72850132	104812861	6	rs12198723	30796734	10	rs10821778	62266408
10	rs10883824	104812897	6	rs3130785	30796738	10	rs10430492	62267108
10	rs2275271	104814162	6	rs12198842	30796932	10	rs80143165	62268958
10	rs7916476	104815268	6	rs9368644	30797083	10	rs117641222	62269394
10	rs12769080	104815345	6	rs9262218	30797215	10	rs2153525	62269574
10	rs11191535	104815876	6	rs7749924	30797991	10	rs17816915	62269683
10	rs41287478	104816469	6	rs3094110	30799043	10	rs10761510	62270037
10	rs7908280	104816947	6	rs3130647	30799072	10	rs2211220	62271491
10	rs79205575	104817383	6	rs3130648	30800326	10	rs1954656	62272646
10	rs11191537	104817709	6	rs1264344	30800577	10	rs10994393	62273627
10	rs185913447	104818716	6	rs1264341	30802465	10	rs79195719	62274173
10	rs12415468	104821191	6	rs3095352	30805921	10	rs10821779	62274525
10	rs17727044	104821256	6	rs11757306	30807058	10	rs9633553	62274737
10	rs117460856	104821768	6	rs3130786	30807948	10	rs10994394	62275329
10	rs12780116	104821946	6	rs2517582	30808762	10	rs10821781	62275515
10	rs77827514	104822009	6	rs3130787	30809864	10	rs10821782	62275564
10	rs80200167	104822262	6	rs2517580	30810056	10	rs10994395	62275861
10	rs1572578	104822431	6	rs2535336	30810654	10	rs10761514	62276317
10	rs78214351	104822688	6	rs2535335	30812497	10	rs12413264	62276548
10	rs11191540	104822809	6	rs2535332	30813249	10	rs12411842	62276713
10	rs72843968	104823674	6	rs2517576	30815035	10	rs10994396	62276720
10	rs7092029	104823909	6	rs2844664	30815945	10	rs10821785	62277121
10	rs184284297	104824154	6	rs2844663	30815951	10	rs10821786	62277344
10	rs11191542	104824324	6	rs2535331	30816270	10	rs10821787	62277622
10	rs943039	104825132	6	rs3132578	30816865	10	rs7098650	62277679
10	rs17115414	104825152	6	rs2394450	30817501	10	rs10821788	62277739
10	rs12358887	104825578	6	rs3095350	30817866	10	rs1417330	62278391
10	rs3781285	104825665	6	rs2844662	30817879	10	rs2153522	62278812
10	rs74468868	104825846	6	rs2250264	30821187	10	rs12146207	62278842
10	rs943038	104826261	6	rs3095345	30822413	10	rs10994397	62279124
10	rs117908909	104826820	6	rs3130653	30822771	10	rs1340586	62279594
10	rs1926034	104829102	6	rs3130654	30823075	10	rs2393640	62279776

10	rs1926032	104829469	6	rs3916232	30823366	10	rs2393641	62279827
10	rs11191545	104829793	6	rs9295928	30823630	10	rs2105463	62280080
10	rs7910900	104829898	6	rs3130655	30823710	10	rs12269538	62280236
10	rs12570528	104830210	6	rs2844660	30823760	10	rs4948423	62280457
10	rs10883826	104830819	6	rs2844659	30824532	10	rs10994398	62281234
10	rs72843974	104832857	6	rs3095344	30824649	10	rs883591	62281392
10	rs3781283	104833048	6	rs7772269	30825220	10	rs6479715	62281464
10	rs12770390	104833074	6	rs9468841	30825287	10	rs79190732	62282199
10	rs12257941	104833161	6	rs2844657	30829522	10	rs2393642	62282490
10	rs118003084	104833686	6	rs7761138	30830292	10	rs2393643	62282567
10	rs72843977	104834237	6	rs3130791	30831843	10	rs12412135	62282834
10	rs17115419	104834876	6	rs2535326	30832063	10	rs10821789	62285338
10	rs943037	104835919	6	rs3130794	30832810	10	rs7906431	62286187
10	rs943036	104836047	6	rs3132576	30833116	10	rs7906549	62286276
10	rs17727391	104836101	6	rs9262263	30833957	10	rs77097330	62286613
10	rs2296569	104836511	6	rs3095354	30836111	10	rs7906760	62286653
10	rs35647154	104836853	6	rs10947109	30836225	10	rs1938542	62286773
10	rs2296568	104836940	6	rs3130657	30837646	10	rs77967847	62287200
10	rs1046411	104837816	6	rs2535340	30838497	10	rs12766956	62288351
10	rs943035	104839152	6	rs2844654	30838688	10	rs10821790	62288767
10	rs72843983	104839230	6	rs2535339	30839430	10	rs1934754	62289270
10	rs7901197	104840433	6	rs3130795	30839629	10	rs1934755	62289275
10	rs10883829	104840635	6	rs3132573	30840688	10	rs10761515	62289800
10	rs12219901	104840967	6	rs3130796	30840950	10	rs1890926	62290105
10	rs10458729	104841479	6	rs7746003	30842894	10	rs72820991	62290805
10	rs763914	104841587	6	rs1264334	30844260	10	rs76204536	62291050
10	rs726010	104841977	6	rs1264333	30844314	10	rs10994406	62291122
10	rs78414630	104842172	6	rs1264332	30845563	10	rs10761516	62291629
10	rs11191547	104843148	6	rs1264331	30846830	10	rs10740030	62291812
10	rs12785223	104844011	6	rs7756521	30848253	10	rs1938541	62292032
10	rs117639302	104844149	6	rs9295930	30849822	10	rs112922975	62292553
10	rs76325870	104844314	6	rs9501032	30850191	10	rs1892547	62292958
10	rs7092200	104844872	6	rs1264327	30850582	10	rs1892546	62294016
10	rs58279999	104844876	6	rs1264326	30851909	10	rs17817819	62294097
10	rs7074395	104844928	6	rs9468842	30852747	10	rs1574466	62294392
10	rs17727686	104845226	6	rs1264324	30855211	10	rs1938540	62294814
10	rs4917995	104845443	6	rs4618569	30855251	10	rs117350820	62295086
10	rs11191548	104846178	6	rs1264323	30855907	10	rs10821791	62296190
10	rs184517520	104846224	6	rs2229933	30857072	10	rs75779710	62297222
10	rs72843990	104846312	6	rs6457281	30857795	10	rs16915102	62298123
10	rs11191549	104846797	6	rs1264322	30857894	10	rs1938539	62298142
10	rs74233806	104846891	6	rs7743661	30858254	10	rs1938538	62298233
10	rs8139	104848123	6	rs1049622	30858857	10	rs7080540	62298586
10	rs10430665	104848430	6	rs3132572	30861729	10	rs10821792	62298616
10	rs12573199	104848844	6	rs1264318	30863530	10	rs7917458	62299408
10	rs12573200	104848855	6	rs886422	30864279	10	rs1938526	62300383
10	rs10786736	104849116	6	rs1049623	30864829	10	rs11813412	62300751
10	rs12573221	104849144	6	rs2239517	30865115	10	rs11817221	62300818

10	rs3740387	104849468	6	rs2267641	30865204	10	rs1938525	62300929
10	rs3736922	104850632	6	rs2239518	30865725	10	rs7074073	62302218
10	rs11191551	104850835	6	rs1049628	30867106	10	rs78058783	62302479
10	rs10883830	104851285	6	rs8408	30867666	10	rs6479716	62302558
10	rs17094683	104851301	6	rs9468843	30867958	10	rs10994408	62302809
10	rs11191553	104851396	6	rs2894055	30868628	10	rs10761517	62302991
10	rs12217501	104851889	6	rs9468845	30869593	10	rs79306853	62303175
10	rs12220743	104851912	6	rs3869086	30870168	10	rs1938524	62303573
10	rs56036640	104852002	6	rs9468846	30870763	10	rs10821793	62303835
10	rs111842178	104852121	6	rs2894054	30872274	10	rs17239789	62303898
10	rs12355120	104852166	6	rs1264312	30872982	10	rs80214098	62304474
10	rs3781282	104852419	6	rs3909130	30874165	10	rs10994411	62304808
10	rs3781281	104852648	6	rs3130780	30874308	10	rs2186251	62304870
10	rs138626837	104852659	6	rs2284175	30875145	10	rs7072878	62305640
10	rs72843997	104853290	6	rs2284176	30875622	10	rs61855348	62307478
10	rs2274340	104853577	6	rs1264309	30875899	10	rs76847929	62307850
10	rs72843998	104855261	6	rs2074510	30876034	10	rs10761518	62307921
10	rs11191554	104855278	6	rs2074508	30876438	10	rs10821795	62308519
10	rs1926030	104855656	6	rs1362123	30876996	10	rs4540900	62308734
10	rs1926029	104855670	6	rs916920	30877202	10	rs79994045	62311027
10	rs12412038	104856162	6	rs3218814	30878579	10	rs2893839	62311190
10	rs184992072	104856766	6	rs2074512	30878919	10	rs2393644	62311274
10	rs187747996	104857206	6	rs886420	30879636	10	rs117897830	62311739
10	rs10786737	104857218	6	rs1264308	30879987	10	rs72822817	62311744
10	rs10883831	104857234	6	rs3218822	30880336	10	rs1892545	62312449
10	rs11191555	104857523	6	rs1264307	30880757	10	rs1938523	62312500
10	rs11191556	104858144	6	rs1264305	30882277	10	rs72822818	62312932
10	rs2274339	104860053	6	rs1264304	30882415	10	rs10761519	62313113
10	rs41287482	104860933	6	rs1264303	30882513	10	rs7101298	62313176
10	rs72844002	104861736	6	rs1264302	30882634	10	rs7085315	62313241
10	rs11191557	104864614	6	rs6926224	30882689	10	rs79479106	62313349
10	rs11191558	104864678	6	rs1264301	30882781	10	rs10740031	62313808
10	rs2281877	104866062	6	rs6926723	30882803	10	rs2893840	62314015
10	rs80122405	104866815	6	rs1264300	30882856	10	rs75700446	62314538
10	rs7896519	104866863	6	rs1264299	30883920	10	rs76349630	62315425
10	rs7896547	104866958	6	rs1264298	30884436	10	rs1938546	62315986
10	rs11191560	104869038	6	rs1264297	30884806	10	rs2154392	62317486
10	rs10786738	104869451	6	rs9262289	30885686	10	rs7088259	62318852
10	rs12413046	104871204	6	rs9262290	30885821	10	rs7070867	62319277
10	rs10883832	104871279	6	rs9262293	30886350	10	rs6479717	62319288
10	rs2066323	104871361	6	rs7756286	30887311	10	rs2393646	62319434
10	rs7899084	104872547	6	rs7738138	30887344	10	rs2068043	62320330
10	rs9633712	104873761	6	rs2285319	30887972	10	rs117685611	62320623
10	rs7908960	104875304	6	rs2249464	30888161	10	rs1975443	62321342
10	rs1935323	104877035	6	rs7766094	30888617	10	rs10994415	62322034
10	rs183804001	104877408	6	rs2517467	30889260	10	rs10740032	62322385
10	rs1926037	104878010	6	rs2074511	30889389	10	rs958852	62322618
10	rs11191564	104878543	6	rs2517466	30890055	10	rs723011	62322849

10	rs6584539	104879402	6	rs885905	30890431	10	rs1442552	62323229
10	rs10748837	104880236	6	rs2074506	30890483	10	rs7914139	62323419
10	rs75221325	104880773	6	rs753725	30890871	10	rs1822261	62323611
10	rs10883834	104881402	6	rs2252760	30892377	10	rs1442551	62324491
10	rs79331374	104882913	6	rs2532941	30892521	10	rs10509133	62324528
10	rs1060240	104883337	6	rs2252856	30893251	10	rs10509132	62325665
10	rs7067663	104883650	6	rs1043483	30893728	10	rs1938537	62326370
10	rs10883835	104884208	6	rs2532938	30893831	10	rs2154393	62326687
10	rs1541213	104885330	6	rs4678	30893941	10	rs74318560	62330507
10	rs11191568	104886374	6	rs2532936	30894408	10	rs17818833	62330759
10	rs12779263	104886533	6	rs2532935	30894573	10	rs7895202	62331684
10	rs10883836	104891198	6	rs2532934	30894759	10	rs6479718	62331983
10	rs34104646	104896078	6	rs4711247	30895680	10	rs7903279	62333324
10	rs72844301	104896100	6	rs3873332	30895990	10	rs1442547	62333559
10	rs72846103	104896751	6	rs3873334	30896147	10	rs74659950	62333617
10	rs11191573	104896816	6	rs2517462	30896190	10	rs7924098	62334197
10	rs746293	104897254	6	rs2517459	30897022	10	rs1442549	62334300
10	rs732998	104897901	6	rs2532929	30897774	10	rs1442550	62334468
10	rs11598702	104897985	6	rs2532927	30898434	10	rs77736959	62335061
10	rs11191575	104898337	6	rs2532925	30899163	10	rs7081442	62336956
10	rs116976910	104898422	6	rs3095153	30899195	10	rs7081445	62336970
10	rs12262258	104900272	6	rs4713402	30899233	10	rs75849617	62337474
10	rs78436955	104900841	6	rs2253588	30899377	10	rs78424567	62337884
10	rs79993475	104901031	6	rs3131787	30899524	10	rs7906410	62339285
10	rs12220375	104901491	6	rs2286656	30899571	10	rs7906839	62339656
10	rs11191577	104902165	6	rs2286655	30899746	10	rs2393671	62340712
10	rs7912517	104905529	6	rs2253705	30900094	10	rs2393672	62340856
10	rs11191580	104906211	6	rs3095151	30900150	10	rs4948262	62342037
10	rs12243903	104906992	6	rs12525616	30900435	10	rs1938527	62342519
10	rs2066322	104909339	6	rs2844651	30900664	10	rs9731224	62343148
10	rs1163249	104909890	6	rs7764239	30900919	10	rs10821797	62344699
10	rs12775302	104911356	6	rs10947113	30902009	10	rs7072678	62344769
10	rs11191582	104913653	6	rs2844650	30902533	10	rs12414575	62344939
10	rs7067970	104913870	6	rs3131784	30903948	10	rs16915156	62345073
10	rs74233809	104913940	6	rs12697941	30904714	10	rs10994420	62345803
10	rs2148198	104915310	6	rs3132571	30905313	10	rs4357640	62346041
10	rs76558380	104915471	6	rs6933400	30907176	10	rs6479719	62346137
10	rs3977751	104920232	6	rs6933909	30907266	10	rs7922717	62346398
10	rs10883839	104920341	6	rs3131921	30907335	10	rs16915157	62346638
10	rs35745742	104922870	6	rs4711248	30907999	10	rs7911164	62347839
10	rs10509765	104924820	6	rs4711249	30908266	10	rs61853474	62348709
10	rs12773916	104924838	6	rs13206144	30912382	10	rs10761523	62349259
10	rs4917996	104925829	6	rs9501035	30912414	10	rs7081184	62349324
10	rs10509766	104926334	6	rs2844702	30912481	10	rs117908493	62349404
10	rs75839032	104928164	6	rs2844701	30912691	10	rs10761524	62349506
10	rs35195396	104928770	6	rs3130781	30914552	10	rs2393673	62350366
10	rs12416331	104928914	6	rs2517451	30914751	10	rs78885952	62350378
10	rs182441718	104934398	6	rs3130782	30914843	10	rs75989863	62350465

10	rs10883841	104934709	6	rs13200483	30916259	10	rs2393675	62350748
10	rs12261294	104934952	6	rs3094086	30919391	10	rs10740035	62352606
10	rs10786740	104935593	6	rs2517449	30919701	10	rs10761526	62354419
10	rs10786741	104939182	6	rs11970154	30919878	10	rs1009410	62354797
10	rs12240508	104939232	6	rs3132580	30920124	10	rs7477771	62355728
10	rs77602755	104939538	6	rs2240804	30920890	10	rs1348281	62356233
10	rs56168342	104940867	6	rs2240803	30920957	10	rs1009677	62357645
10	rs79237883	104940946	6	rs12190030	30921364	10	rs10509131	62358361
10	rs11191595	104943048	6	rs3757340	30921882	10	rs10994423	62359130
10	rs3758543	104944744	6	rs2532921	30922570	10	rs16915196	62359859
10	rs10786744	104945028	6	rs12212418	30924024	10	rs75525457	62359891
10	rs7077097	104945463	6	rs11753326	30925985	10	rs56085753	62360027
10	rs7077291	104945751	6	rs11752177	30927867	10	rs10994424	62360081
10	rs77420391	104945823	6	rs4711250	30930270	10	rs55849269	62360085
10	rs10883843	104947493	6	rs6928738	30931059	10	rs1442544	62360930
10	rs7920251	104950197	6	rs3131934	30931844	10	rs10761527	62361113
10	rs79780963	104952499	6	rs3131783	30932068	10	rs10994425	62361400
10	rs115952365	104952587	6	rs2844697	30932309	10	rs75645667	62361525
10	rs10748839	104953547	6	rs3095150	30932532	10	rs10994426	62362164
10	rs79179549	104953734	6	rs2530715	30933517	10	rs10159808	62362190
10	rs117569499	104953892	6	rs3095089	30933794	10	rs10160182	62362451
10	rs11191602	104954219	6	rs2254847	30933848	10	rs10159993	62362546
10	rs145267701	104954778	6	rs3131933	30933864	10	rs12782869	62362772
10	rs7095304	104954795	6	rs2532919	30933975	10	rs9415604	62362777
10	rs11191606	104956035	6	rs3095085	30937377	10	rs1372710	62362958
10	rs7100369	104956218	6	rs9368649	30938883	10	rs117339800	62364018
10	rs10786745	104956827	6	rs3131932	30940328	10	rs118126632	62364597
10	rs12571345	104957152	6	rs2530710	30940387	10	rs7098097	62364991
10	rs10883846	104958244	6	rs2255625	30940705	10	rs4584525	62365060
10	rs10883847	104958284	6	rs3132579	30940989	10	rs10740036	62365974
10	rs12774577	104959487	6	rs2255719	30941285	10	rs7894641	62366430
10	rs75075426	104960856	6	rs2530708	30941691	10	rs80284429	62366621
10	rs11191608	104961119	6	rs2517439	30942929	10	rs10821800	62367570
10	rs12259163	104961477	6	rs3131931	30945265	10	rs10761528	62367660
10	rs4917999	104963051	6	rs2844682	30946148	10	rs1006956	62367976
10	rs12256506	104963618	6	rs2844680	30946496	10	rs1006955	62368339
10	rs11191609	104963721	6	rs9394023	30947042	10	rs61853514	62369984
10	rs11818627	104964536	6	rs2844679	30947365	10	rs17819756	62370465
10	rs73353848	104964620	6	rs2517431	30947378	10	rs6479720	62370693
10	rs72846141	104976963	6	rs2517425	30949670	10	rs10994429	62371079
10	rs77664249	104978553	6	rs2517424	30949996	10	rs2165691	62371649
10	rs113987398	104979168	6	rs2517421	30951209	10	rs10821801	62371775
10	rs73353870	104979954	6	rs9295938	30953105	10	rs10994430	62371953
10	rs183592323	104980983	6	rs2530699	30953240	10	rs6479721	62373122
10	rs74780752	104989168	6	rs9295939	30953968	10	rs1442543	62373462
10	rs55939028	104990305	6	rs2844677	30955359	10	rs4143096	62374033
10	rs7922982	104990933	6	rs1634731	30955681	10	rs1837947	62375033
10	rs79850157	104993304	6	rs2517416	30955929	10	rs16915231	62375046

10	rs4917384	104995788	6	rs2517415	30956779	10	rs10884907	111530413
10	rs12761878	104997012	6	rs886403	30957618	10	rs540258	111591977
10	rs6584545	104999266	6	rs3873342	30957766	10	rs10884914	111594630
10	rs7913461	105001325	6	rs2249168	30958254	10	rs11194888	111597714
10	rs4918001	105013147	6	rs2530695	30958494	10	rs11599348	111611304
10	rs7093667	105080575	6	rs2517411	30960267	10	rs7918778	111611835
10	rs12770682	105116465	6	rs2844673	30961926	10	rs12767800	111663558
10	rs11191672	105138961	6	rs2517409	30964393	10	rs10509907	111781715
11	rs10834330	24398195	6	rs2252926	30966304	10	rs1349348	111794554
11	rs2291487	46456344	6	rs2517408	30966453	10	rs2501578	111838873
11	rs12574250	46491159	6	rs2429295	30969498	10	rs61745504	111987960
11	rs7106740	46511497	6	rs2508016	30969527	11	rs174868	61371283
11	rs17197116	46520302	6	rs12196237	30969781	11	rs903778	61435742
11	rs7949282	46522306	6	rs2253417	30970459	11	rs3931642	61462424
11	rs10160701	46551918	6	rs6927057	30970879	11	rs12274157	61477647
11	rs11038913	46559730	6	rs11964080	30970970	11	rs4963243	61494327
11	rs12290779	46594971	6	rs1634717	30972589	11	rs2240287	61505583
11	rs35619591	46690413	6	rs1634718	30972865	11	rs198443	61505696
11	rs8914	46699124	6	rs1634719	30973165	11	rs61896065	61506250
11	rs74486166	46700203	6	rs2523915	30973358	11	rs198442	61506468
11	rs72912170	46701108	6	rs1624944	30973535	11	rs736121	61506584
11	rs10838612	46701728	6	rs2523921	30975090	11	rs4963255	61507606
11	rs200777546	46702160	6	rs2523924	30975224	11	rs11828739	61508074
11	rs4319473	46702691	6	rs1632854	30975649	11	rs198441	61508379
11	rs12574259	46704439	6	rs2844666	30976781	11	rs198440	61508522
11	rs61884297	46705974	6	rs1634721	30977680	11	rs198439	61509589
11	rs72912177	46718663	6	rs2523926	30977815	11	rs3741252	61511498
11	rs28372918	46722018	6	rs2530716	30978196	11	rs61896068	61513400
11	rs7932354	46722221	6	rs13191258	30978717	11	rs139937518	61513675
11	rs57739486	46723516	6	rs12203410	30978746	11	rs198424	61513725
11	rs10769205	46723603	6	rs1634723	30979156	11	rs2852786	61514085
11	rs12361673	46723937	6	rs9366764	30979793	11	rs198423	61514670
11	rs145722187	46724722	6	rs3869095	30980015	11	rs117494202	61514757
11	rs58922799	46725196	6	rs12528087	30980603	11	rs4963390	61514821
11	rs11038973	46728952	6	rs12528082	30980747	11	rs198422	61515767
11	rs11038977	46734384	6	rs9262464	30980848	11	rs147252389	61515870
11	rs61884305	46735956	6	rs9380205	30981361	11	rs3825036	61516476
11	rs77920005	46737057	6	rs7755364	30981715	11	rs3741255	61516910
11	rs2070850	46741495	6	rs9380206	30981755	11	rs1615167	61518986
11	rs3136433	46741908	6	rs1634725	30982008	11	rs198419	61519542
11	rs3136435	46742426	6	rs7755802	30982209	11	rs77053629	61519815
11	rs3136436	46742489	6	rs12211072	30982948	11	rs569258	61520668
11	rs3136439	46743135	6	rs9391701	30983263	11	rs198465	61521318
11	rs3136520	46743232	6	rs1619376	30983326	11	rs520987	61521446
11	rs3136441	46743247	6	rs3871466	30983683	11	rs198464	61521621
11	rs3136447	46744368	6	rs7776233	30984128	11	rs10709102	61523301
11	rs3136448	46744369	6	rs3869096	30984404	11	rs2238003	61523735
11	rs3136449	46744470	6	rs9262492	30986015	11	rs198462	61524119

11	rs2070851	46744528	6	rs2894176	30986038	11	rs198461	61524366
11	rs2070852	46744925	6	rs3906268	30986079	11	rs2238001	61524507
11	rs5896	46745003	6	rs3873345	30986264	11	rs198459	61525020
11	rs3136456	46745861	6	rs3906267	30986396	11	rs198476	61525730
11	rs3136457	46746258	6	rs9262495	30986527	11	rs198475	61526071
11	rs3136460	46747065	6	rs4585609	30986560	11	rs198473	61526556
11	rs1799867	46747979	6	rs12215548	30986644	11	rs198472	61527266
11	rs5898	46749648	6	rs9262498	30986835	11	rs695186	61527927
11	rs3136472	46750064	6	rs9295942	30987010	11	rs198471	61528101
11	rs3136524	46750525	6	rs9262499	30987098	11	rs198470	61528306
11	rs3136474	46750819	6	rs12208652	30987170	11	rs400075	61528814
11	rs5900	46751059	6	rs4713411	30987176	11	rs12224732	61529830
11	rs1783828	57411521	6	rs4713412	30987211	11	rs2071212	61530837
11	rs10896633	57424040	6	rs9402255	99332848	11	rs10792318	61531443
11	rs11229087	57425699	6	rs10484608	99335648	11	rs79519287	61531810
11	rs1798173	57432057	6	rs34316889	99374760	11	rs55903902	61531991
11	rs503037	57436530	6	rs7771685	99395018	11	rs4963418	61533797
11	rs1647405	57477889	6	rs910423	99476741	11	rs113004932	61533908
11	rs11607056	57496820	6	rs12201236	99478174	11	rs2071213	61534010
11	rs2847308	57498232	6	rs17059074	99484649	11	rs547589	61534872
11	rs9420	57510294	6	rs761636	99535539	11	rs650436	61536430
11	rs7126553	57556673	6	rs12190827	99550419	11	rs579383	61536583
11	rs11570190	57560452	6	rs6569850	99551277	11	rs2269928	61537529
11	rs665058	57579166	6	rs6905786	99555756	11	rs174527	61538401
11	rs708228	57585662	6	rs9375932	99566736	11	rs149803	61539020
11	rs7936998	57639017	6	rs2388839	99574363	11	rs628993	61539691
11	rs7117205	57641883	6	rs4454147	99575874	11	rs174528	61543499
11	rs10890995	109327622	6	rs728758	99584658	11	rs174529	61543961
11	rs10890996	109328421	6	rs1003243	99594876	11	rs174530	61546592
11	rs1992276	109358598	6	rs1496967	99597105	11	rs7943728	61547068
11	rs7129464	109530475	6	rs12110525	99629252	11	rs72920160	61547951
11	rs1799978	113346351	6	rs6910466	99643679	11	rs61747222	61548796
11	rs4938021	113364803	6	rs7769752	99662681	11	rs174532	61548874
11	rs10891567	113398627	6	rs9375997	99673152	11	rs174535	61551356
11	rs2514227	113404170	6	rs11154755	99673981	11	rs174537	61552680
11	rs11214654	113410917	6	rs7757056	99680609	11	rs17762402	61553201
11	rs12222458	113417603	6	rs9376014	99690090	11	rs61896141	61556039
11	rs10736469	113418222	6	rs1874538	99694494	11	rs12786694	61556074
11	rs7927176	123395864	6	rs9389197	99736465	11	rs117110139	61557422
11	rs2075713	124617939	6	rs9402636	99746934	11	rs102275	61557803
11	rs12278050	133809032	6	rs2076517	99748757	11	rs740006	61557868
11	rs10444323	133811160	6	rs9402648	99750162	11	rs174538	61560081
11	rs4937860	133814713	6	rs9399120	99750197	11	rs412334	61560261
11	rs493888	133832473	6	rs9402650	99751460	11	rs695867	61561288
11	rs329658	133849281	6	rs9373111	99751681	11	rs193250731	61567670
12	rs1016388	2321868	6	rs9399123	99753078	11	rs174546	61569830
12	rs12424245	2322513	6	rs9389252	99773908	11	rs174547	61570783
12	rs200292772	2323144	6	rs221525	99774241	11	rs174548	61571348

12	rs139758774	2323145	6	rs9399134	99774687	11	rs174549	61571382
12	rs2283290	2323621	6	rs9376088	99778293	11	rs174550	61571478
12	rs11614275	2323846	6	rs9376089	99778405	11	rs174553	61575158
12	rs7957545	2324042	7	rs6959385	12031826	11	rs174555	61579760
12	rs2238055	2324407	7	rs2192735	12033933	11	rs174556	61580635
12	rs11062158	2325978	7	rs10280298	12049985	11	rs174558	61581450
12	rs2238056	2327944	7	rs11982169	12051898	11	rs174560	61581764
12	rs72653432	2329572	7	rs2966619	12052059	11	rs762359509	61582527
12	rs11062161	2329970	7	rs17165507	12053458	11	rs174561	61582708
12	rs10774034	2330458	7	rs11977758	12065305	11	rs116980792	61585140
12	rs12228890	2330997	7	rs2908738	12065405	11	rs174562	61585144
12	rs3922316	2331144	7	rs17581371	12090560	11	rs174566	61592362
12	rs2190771	2331192	7	rs17581713	12105982	11	rs174568	61593816
12	rs10848642	2331572	7	rs4721023	12112471	11	rs174569	61594164
12	rs11062162	2332104	7	rs2192744	12123472	11	rs191508698	61594921
12	rs2239028	2332210	7	rs12699309	12137596	11	rs968567	61595564
12	rs76799633	2332371	7	rs1003433	12164100	11	rs99780	61596633
12	rs4765904	2332393	7	rs1003434	12164223	11	rs174570	61597212
12	rs1108221	2332795	7	rs12536822	12196391	11	rs1535	61597972
12	rs1108222	2332856	7	rs6952272	12200060	11	rs117553420	61599329
12	rs1108074	2333484	7	rs1369491	12212027	11	rs61897793	61599347
12	rs1108073	2333638	7	rs6460933	12391454	11	rs11230803	61599991
12	rs1304749689	2333671	7	rs10267441	12394202	11	rs174573	61600327
12	rs77893928	2333743	7	rs17165936	12414725	11	rs174574	61600342
12	rs55802595	2333877	7	rs1100136	12491829	11	rs2524296	61601378
12	rs75729273	2334076	7	rs12667501	12498511	11	rs2845574	61601872
12	rs11062166	2334260	7	rs17166041	12506732	11	rs2845573	61601908
12	rs7312354	2334444	7	rs10259444	108863910	11	rs174575	61602003
12	rs7296039	2334933	7	rs10230075	108864051	11	rs72920193	61602572
12	rs7977619	2335293	7	rs6954533	108872792	11	rs2727271	61603358
12	rs2239030	2335941	7	rs848896	108960029	11	rs174576	61603510
12	rs2239031	2336169	7	rs1100302	108980725	11	rs117220229	61604324
12	rs76314765	2336323	7	rs848866	108987486	11	rs2524299	61604782
12	rs2239032	2337138	7	rs848855	108991939	11	rs174577	61604814
12	rs11613885	2337370	7	rs1406304	109055726	11	rs2072113	61604967
12	rs2283291	2337460	7	rs10256936	109085010	11	rs2072114	61605215
12	rs2239033	2337746	7	rs13222164	109098364	11	rs174578	61605499
12	rs3794297	2338396	7	rs7778502	109101305	11	rs75992720	61605578
12	rs68089646	2338756	7	rs10499972	109109099	11	rs174579	61605613
12	rs6489353	2338858	7	rs1721902	109209066	11	rs174580	61606642
12	rs3829331	2339250	7	rs1626408	109237961	11	rs174581	61606683
12	rs3794296	2339253	7	rs2063937	109266627	11	rs174582	61607168
12	rs117433140	2340001	7	rs17156981	109269769	11	rs80349011	61607457
12	rs34382810	2340798	7	rs10246950	109279682	11	rs17156426	61609323
12	rs7965923	2341385	7	rs10275927	109350011	11	rs10897180	61609448
12	rs7303977	2342248	7	rs4730399	109352652	11	rs11230807	61609708
12	rs7135319	2342417	9	rs1378062	2751726	11	rs174583	61609750
12	rs7305301	2342636	9	rs10967953	2756638	11	rs75766519	61611033

12	rs6489354	2343186	9	rs7857841	2756756	11	rs117983270	61611710
12	rs76086674	2343344	9	rs1014240	2801007	11	rs11601892	61612560
12	rs11609729	2343551	9	rs7850317	2809548	11	rs174587	61612830
12	rs117924617	2344253	9	rs10757715	2827752	11	rs12284876	61613841
12	rs117388872	2344560	9	rs2270891	2828742	11	rs1790277	61613889
12	rs769087	2344644	9	rs2173904	2828765	11	rs17156442	61614023
12	rs2007044	2344960	9	rs12344362	2841896	11	rs7935946	61615542
12	rs1006737	2345295	9	rs10757741	2851381	11	rs2851682	61616012
12	rs11062169	2345390	9	rs4013197	2921825	11	rs78791707	61616069
12	rs77013632	2345479	9	rs10156534	2936864	11	rs75471190	61616322
12	rs11610437	2345625	9	rs3847230	2937654	11	rs174591	61617676
12	rs75269774	2345796	9	rs17657377	3012175	11	rs79303190	61618016
12	rs11614966	2346254	9	rs2876342	3079435	11	rs61897795	61618169
12	rs2159100	2346393	9	rs7869695	3175247	11	rs174592	61618608
12	rs12315711	2346830	9	rs13288589	3182885	11	rs174593	61618831
12	rs2239034	2347694	9	rs10970529	3189170	11	rs916924	61619181
12	rs11062170	2348844	9	rs12342853	3192845	11	rs916925	61619294
12	rs2239035	2349081	9	rs6476327	3215842	11	rs174597	61621040
12	rs2239036	2349141	9	rs17658489	3220602	11	rs174598	61621194
12	rs11612837	2349406	9	rs10813760	3223166	11	rs174599	61621556
12	rs4765905	2349584	9	rs4741148	11339109	11	rs73487492	61621611
12	rs7975467	2350263	9	rs10809441	11366549	11	rs174601	61623140
12	rs882195	2350401	9	rs16927615	11462576	11	rs2526678	61623793
12	rs882194	2350452	9	rs10119070	11573922	11	rs97384	61624181
12	rs882193	2350620	9	rs13296444	11610054	11	rs174602	61624414
12	rs80295761	2352835	9	rs2821184	11620981	11	rs498793	61624705
12	rs77175673	2353407	9	rs10960066	11649791	11	rs526126	61624885
12	rs78185961	2354263	9	rs13296683	11700487	11	rs174605	61626921
12	rs73033370	2354530	9	rs10960116	11708630	11	rs174608	61627484
12	rs80071235	2354582	9	rs10125636	119489471	11	rs174609	61627811
12	rs2370413	2354870	9	rs10120611	119523145	11	rs174610	61627833
12	rs7967379	2355460	9	rs10817934	119550051	11	rs174611	61627881
12	rs7297582	2355806	9	rs1155556	119572334	11	rs74771917	61627960
12	rs11608296	2356371	9	rs13285982	119579244	11	rs174615	61628964
12	rs79446827	2356554	9	rs12237386	119589080	11	rs174616	61629122
12	rs79976249	2356940	9	rs943309	119599526	11	rs174617	61629166
12	rs78661331	2358805	9	rs1326815	119618167	11	rs113003506	61629377
12	rs75892392	2359453	9	rs10513280	119685920	11	rs11230813	61629576
12	rs11615998	2360166	9	rs10817960	119688061	11	rs174619	61629666
12	rs758170	2361460	9	rs7021489	119719693	11	rs174620	61629747
12	rs11609090	2361915	9	rs1537528	119771893	11	rs174621	61630104
12	rs11611154	2362006	9	rs10817983	119808697	11	rs639394	61631331
12	rs7309075	2362116	9	rs10983517	119858116	11	rs518511	61631454
12	rs7138478	2362127	9	rs2050272	119877837	11	rs3168072	61631510
12	rs61907761	2362198	9	rs12003386	126448659	11	rs3741	61631614
12	rs7139112	2362597	9	rs12336857	126453684	11	rs12577276	61632310
12	rs116644100	2362890	9	rs7857196	126465958	11	rs482548	61633182
12	rs2239037	2363716	9	rs10986093	126494022	11	rs11539526	61633770

12	rs11612720	2363789	9	rs12339991	126511085	11	rs113268188	61634829
12	rs74347853	2364229	9	rs2479106	126525212	11	rs174623	61635294
12	rs180703721	2364443	9	rs1752156	126534512	11	rs7395424	61636017
12	rs76139517	2364448	9	rs2799466	126548090	11	rs11230815	61636126
12	rs79164695	2364685	9	rs1752166	126558843	11	rs11230816	61636253
12	rs191226186	2365290	9	rs2489159	126563910	11	rs11230817	61636255
12	rs79472832	2365480	9	rs12337273	126566945	11	rs174626	61637057
12	rs78308994	2365589	9	rs7863739	126601256	11	rs7394459	61637349
12	rs76836074	2365643	9	rs589766	126629491	11	rs174627	61637466
12	rs4765910	2365903	9	rs2041545	126650515	11	rs174628	61637587
12	rs115124024	2365908	9	rs10986126	126655730	11	rs7104849	61638044
12	rs116642590	2367096	9	rs677987	126656997	11	rs10897181	61638234
12	rs114364574	2367790	9	rs2479102	126683955	11	rs79479642	61638953
12	rs79091024	2368141	9	rs12335840	126713411	11	rs508768	61639335
12	rs2370414	2368396	9	rs7867942	126771321	11	rs7118175	61639358
12	rs10774035	2368674	9	rs80119968	126791907	11	rs422249	61639488
12	rs73033378	2368821	9	rs6478638	126809736	11	rs174448	61639573
12	rs117703678	2370776	9	rs7867375	126828640	11	rs7481842	61639705
12	rs2283292	2371652	9	rs4838102	126829238	11	rs7482316	61640198
12	rs80191236	2373810	9	rs2638381	126894348	11	rs174449	61640379
12	rs11615388	2374006	9	rs2767762	126897258	11	rs6591664	61640521
12	rs2239038	2374130	9	rs2638384	126899452	11	rs75306442	61641396
12	rs11608869	2374463	9	rs2807590	126899565	11	rs174450	61641542
12	rs74767351	2374646	9	rs10818924	126913862	11	rs7115739	61641717
12	rs79122070	2375199	9	rs2767770	126914307	11	rs7942717	61647288
12	rs11616036	2375612	9	rs11560587	126931710	11	rs174634	61647387
12	rs1006690	2375636	10	rs1855581	106371784	11	rs174635	61647427
12	rs2159101	2375687	10	rs10748866	106391762	11	rs116672159	61648113
12	rs7485359	2375793	10	rs998300	106398315	11	rs7930349	61649211
12	rs77205646	2376027	10	rs4918123	106405854	11	rs174454	61650747
12	rs116229134	2376168	10	rs6584627	106537058	11	rs116139751	61653164
12	rs78155648	2376292	10	rs4144552	106541709	11	rs1000778	61655305
12	rs80284141	2376294	10	rs2451464	106555151	11	rs174455	61656117
12	rs75548797	2376651	10	rs1565419	106571060	11	rs174456	61656182
12	rs74393821	2376815	10	rs1032317	106579347	11	rs174460	61657110
12	rs1476733	2376900	10	rs1873308	106580136	11	rs174461	61657401
12	rs79399264	2377952	10	rs1404790	106694770	11	rs68112215	61657593
12	rs116014402	2378234	10	rs11192286	106786090	11	rs174462	61657666
12	rs11614796	2379100	11	rs7942007	31835418	11	rs693672	61658629
12	rs11609165	2379874	11	rs6484540	31847791	11	rs6591668	61661113
12	rs11615807	2380232	11	rs624732	31869836	11	rs174468	61663691
12	rs80106079	2381056	11	rs1524143	31871538	11	rs17764935	61664757
12	rs79411638	2381569	11	rs7935771	31872707	11	rs13966	61664992
12	rs12298346	2381910	11	rs12363571	31883249	11	rs2235093	61665122
12	rs114723538	2382549	11	rs621611	31896170	11	rs174469	61667443
12	rs4488257	2382964	11	rs652722	31905534	11	rs112543847	61667879
12	rs2370415	2383050	11	rs683028	31922575	11	rs3815045	61669946
12	rs117565726	2383682	11	rs2014429	31941035	11	rs174471	61670037

12	rs2238057	2384005	11	rs10734409	32033826	11	rs117828446	61670646
12	rs76777168	2384042	11	rs4140451	32055285	11	rs12420820	61670745
12	rs74992059	2384150	11	rs224618	32064649	11	rs174472	61671956
12	rs74411248	2384223	11	rs224619	32065256	11	rs111902278	61672147
12	rs79426448	2384357	11	rs2063016	32069871	11	rs174473	61672235
12	rs77558515	2384432	11	rs224634	32080615	11	rs174474	61672265
12	rs7969407	2384768	11	rs11031563	32080766	11	rs174475	61672645
12	rs61907778	2384815	11	rs224630	32087108	11	rs174476	61674118
12	rs77759718	2384894	11	rs11031570	32092617	11	rs61898562	61676505
12	rs7979604	2385018	12	rs7963001	23770935	11	rs61898564	61676897
12	rs76173403	2385283	12	rs10771012	23788075	11	rs174478	61678576
12	rs76721755	2386415	12	rs11047025	23805223	11	rs174479	61678754
12	rs75705537	2386772	12	rs4963708	23821733	11	rs61898566	61679961
12	rs10774036	2386948	12	rs2900524	23842214	11	rs117324079	61680386
12	rs10744560	2387099	12	rs4293213	23923620	11	rs540613	61680870
12	rs79189017	2387245	12	rs7968921	23960177	11	rs2524287	61684550
12	rs118004017	2387701	12	rs2971157	23962011	11	rs77229376	61685791
12	rs75728817	2388507	12	rs9804988	23982559	11	rs78156005	61685950
12	rs114567313	2388653	12	rs10505911	24022160	11	rs2521561	61685989
12	rs7972103	2388720	12	rs12579703	24023486	11	rs2524288	61686336
12	rs34335441	2389220	12	rs4439603	24044286	11	rs2524289	61687033
12	rs77577013	2390132	12	rs7136898	24054572	11	rs76133863	61687130
12	rs78184324	2391325	12	rs4581551	24057198	11	rs11230827	61687454
12	rs77704787	2391412	12	rs7970953	24075508	11	rs187943834	61687926
12	rs74630743	2391544	12	rs7139333	24127584	11	rs73491252	61688705
12	rs79832477	2391562	12	rs16927028	24190428	11	rs78243280	61688922
12	rs115500284	2394025	13	rs1329297	44204553	11	rs116985542	61689275
12	rs76408048	2394434	13	rs9525814	44221209	11	rs190738753	61690079
12	rs78525694	2394494	13	rs9525816	44223898	11	rs2727258	61690183
12	rs78161460	2394704	13	rs17538959	44229911	11	rs12420625	61690405
12	rs12311439	2394781	13	rs9594987	44230994	11	rs77071864	61690420
12	rs78683530	2394935	13	rs9594989	44237392	11	rs741888	61690544
12	rs117311450	2395626	13	rs74436076	44238254	11	rs741887	61690707
12	rs11610448	2396153	13	rs9533575	44239055	11	rs7927548	61690901
12	Chr12:2267094	2396833	13	rs7332151	44239219	11	Chr11:61448595	61692019
12	rs1984637	2397108	13	rs883877	44239557	11	rs882169	61692130
12	rs115724978	2397793	13	rs9525821	44239706	11	rs17156580	61692341
12	rs116698755	2399295	13	rs57985080	44239951	11	rs11825591	61693684
12	rs117067220	2399399	13	rs12100382	44240417	11	rs75483554	61694349
12	rs11614494	2399937	13	rs78899204	44240501	11	rs2158026	61694394
12	rs75739143	2401270	13	rs74318190	44240949	11	rs2521564	61696334
12	rs79899792	2401377	13	rs79255569	44241335	11	rs2521565	61696675
12	rs11615645	2402092	13	rs75139428	44241376	11	rs140698203	61696999
12	rs114241316	2402144	13	rs1032520	44241773	11	rs4963275	61697658
12	rs76623174	2402241	13	rs9533576	44242514	11	rs6591673	61697904
12	rs1024582	2402246	13	rs1032521	44242578	11	rs78982937	61697972
12	rs116298939	2402318	13	rs77698779	44243492	11	rs7928792	61698488
12	rs56177308	2402990	13	rs7140060	44243894	11	rs2521567	61699055

12	rs7310491	2404022	13	rs1390369	44245475	11	rs2736604	61699827
12	rs76540614	2405146	13	rs1390370	44245602	11	rs75627029	61700019
12	rs11615601	2405598	13	rs12431105	44248368	11	rs7120114	61700819
12	rs76719316	2406103	13	rs73186153	44249408	11	rs186383379	61700884
12	rs74500489	2406703	13	rs7984720	44249607	11	rs2521568	61700933
12	rs113258895	2407139	13	rs73186155	44249693	11	rs79972157	61702467
12	rs4298967	2408194	13	rs7986170	44249823	11	rs2727273	61702603
12	rs73033382	2408635	13	rs7985113	44249964	11	rs12799580	61702911
12	rs16929233	2411198	13	rs7985732	44250126	11	rs183092825	61703445
12	rs302335	29905322	13	rs9533579	44250236	11	rs61898622	61703756
12	rs302337	29906149	13	rs111389094	44250359	11	rs2158027	61705207
12	rs436124	29916839	13	rs9533580	44250511	11	rs2736602	61705493
12	rs302321	29928388	13	rs9533581	44250640	11	rs2736600	61706011
12	rs302340	29938127	13	rs9316050	44250696	11	rs2524293	61710538
12	rs302342	29939628	13	rs9533582	44250866	11	rs74556176	61710547
12	rs4759273	57444567	13	rs9567245	44251688	11	rs2727269	61710822
12	rs6581128	57583672	13	rs456012	44251815	11	rs76368648	61710872
12	rs1800159	57593894	13	rs9316051	44251976	11	rs2521572	61711475
12	rs7978567	57595984	13	rs9525823	44252093	11	rs77022126	61712646
12	rs73338162	57624701	13	rs55885634	44252172	11	rs1534843	61713495
12	rs2229717	57627074	13	rs73186183	44252230	11	rs1534842	61713590
12	rs117244382	57627525	13	rs9595000	44252415	11	rs74754540	61716357
12	rs34095989	57627717	13	rs117294783	44252904	11	rs972355	61717337
12	rs116973701	57629608	13	rs9316052	44253109	11	rs972354	61717607
12	rs3923885	57634698	13	rs9525824	44253144	11	rs2736597	61718036
12	rs73338169	57635988	13	rs56323657	44253928	11	rs77651946	61718173
12	rs3204635	57637593	13	rs9533583	44254317	11	rs2736596	61719026
12	rs73120041	57638895	13	rs9533584	44254396	11	rs1800007	61719387
12	rs117927148	57640807	13	rs9533585	44254414	11	rs2736595	61720538
12	rs7976512	57643686	13	rs9533586	44254523	11	rs195166	61721584
12	rs10876968	57644767	13	rs9533587	44254672	11	rs2668899	61721639
12	rs11172134	57645789	13	rs299359	44255112	11	rs78054615	61721718
12	rs11172135	57647374	13	rs299356	44255727	11	rs2736594	61722212
12	rs78607331	57648644	13	rs78953565	44256025	11	rs183176	61722265
12	rs73120051	57648772	13	rs9525825	44256095	11	rs1109748	61722645
12	rs4075325	57649840	13	rs9533588	44256623	11	rs195165	61722838
12	rs74240697	57650897	13	rs7996264	44257080	11	rs195164	61723556
12	rs6581131	57652963	13	rs7995938	44257120	11	rs168990	61723859
12	rs7966846	57653582	13	rs114265560	44257437	11	rs195162	61724514
12	rs117621047	57655968	13	rs9533589	44257756	11	rs741886	61724787
12	rs73338185	57656666	13	rs299353	44257782	11	rs2955684	61725174
12	rs11172139	57656705	13	rs8002490	44258023	11	rs2668898	61725498
12	rs73120065	57657753	13	rs9533590	44258085	11	rs195160	61726097
12	rs77936166	57660377	13	rs299351	44258436	11	rs1735379	61726396
12	rs10783818	57662563	13	rs117629391	44258542	11	rs195158	61726871
12	rs73338190	57666612	13	rs188486	44258846	11	rs1801390	61727438
12	rs10783819	57667261	13	rs299350	44259026	11	rs195157	61727791
12	rs79245439	57671622	13	rs17652964	44259648	11	rs57815521	61728147

12	rs73338194	57672511	13	rs7491050	44259766	11	rs195156	61729063
12	rs111393283	57672530	13	rs299348	44260432	11	rs149698	61730036
12	rs7135142	57672913	13	rs299347	44260783	11	rs1800008	61730183
12	rs77784795	57674246	13	rs9595002	44261394	11	rs1800009	61730234
12	rs7397991	57675203	13	rs79007561	44261699	11	rs17185413	61730553
12	rs7302906	57676178	13	rs116933617	44262229	11	rs2668897	61731233
12	rs7133939	57676255	13	rs77550665	44262459	11	rs1801621	61731727
12	rs56383506	57676788	13	rs1994874	44262560	11	rs17156609	61731977
12	rs12227309	57679030	13	rs118131176	44262865	11	rs76059597	61733349
12	rs35007557	57679147	13	rs299344	44263589	11	rs2073588	61736411
12	rs56205943	57679414	13	rs117561237	44264431	11	rs3758977	61737244
12	rs11172142	57680101	13	rs117048727	44264686	11	rs12225574	61737569
12	rs7486863	57681122	13	rs9525826	44265506	11	rs12799017	61738545
12	rs10876969	57682179	13	rs117148223	44265526	11	rs57398279	61739142
12	rs61937595	57682956	13	rs1329292	44266920	11	rs3094402	61739586
12	rs1401994	103606574	13	rs79244847	44268185	11	rs195446	61742966
12	rs11612540	103607581	13	rs634666	44269970	11	rs17156616	61743278
12	rs998499	103611495	13	rs634666	44270227	11	rs17156618	61744672
12	rs4766428	110723245	13	rs117437516	44270610	11	rs17185574	61745694
12	rs3782289	123449432	13	rs17653052	44270635	11	rs10897193	61746269
12	rs11061056	123450867	13	rs9567247	44270727	11	rs76978219	61746274
12	rs116887147	123452527	13	rs395138	44270894	11	rs10792320	61746291
12	rs10734978	123453034	13	rs455004	44271040	11	rs10792321	61746789
12	rs11061080	123453732	13	rs406731	44272398	11	rs71490344	61747018
12	rs7296418	123457619	13	rs953102	44273385	11	rs12575518	61748066
12	rs883562	123460719	13	rs60885954	44273697	11	rs17156632	61748188
12	rs883563	123460823	13	rs11147924	44274277	11	rs10897194	61748802
12	rs884956	123460962	13	rs421787	44274394	11	rs10160397	61749766
12	rs117913606	123461117	13	rs9567250	44274520	11	rs11230849	61750823
12	rs36004829	123461284	13	rs393632	44274730	11	rs11230851	61751148
12	rs3759115	123464279	13	rs430838	44275321	11	rs4423188	61766418
12	rs55742290	123466111	13	rs7987487	44275444	11	rs4963452	61815803
12	rs67306085	123468733	13	rs11618336	44275628	11	rs10897213	61827748
12	rs1568918	123469141	13	rs9533592	44276433	11	rs668210	65783915
12	rs3741530	123469647	13	rs77504430	44277434	11	rs2270448	65835931
12	rs12828755	123470586	13	rs7995406	44277710	11	rs524281	65886662
12	rs12811109	123471094	13	rs8000932	44278906	11	rs801741	65917084
12	rs73230029	123472472	13	rs7985461	44279594	11	rs918299	65927607
12	rs1463877	123473254	13	rs73188061	44279675	11	rs17494956	65992439
12	rs7135296	123476586	13	rs78547599	44279815	11	rs559298	65998757
12	rs937564	123477311	13	rs7984973	44280066	11	rs479018	66060546
12	rs11061244	123478378	13	rs299328	44280605	11	rs560577	66064126
12	rs144623962	123480034	13	rs299329	44281252	11	rs479315	66064991
12	rs74954864	123480323	13	rs669486	44282106	11	rs905770	66156765
12	rs61955214	123482424	13	rs392930	44283226	11	rs7947391	66186882
12	rs190750361	123484647	13	rs117746682	44283733	11	rs569818	66570749
12	rs883263	123485188	13	rs1021549	44283881	11	rs2229745	66617790
12	rs11061275	123485193	13	rs408740	44283997	11	rs7122539	66662731

12	rs77674871	123488393	13	rs419789	44284550	11	rs11227628	66685356
12	rs112484459	123488399	13	rs7993757	44284688	11	rs10791896	66689240
12	rs940904	123491572	13	rs1831257	44293433	11	rs7110302	66690454
12	rs3897102	123492112	13	rs299331	44293845	11	rs7119682	66714782
12	rs12820906	123493123	13	rs12864863	44294500	11	rs11227668	66812338
12	rs117992630	123493772	13	rs299330	44294856	11	rs7112925	66826160
12	rs78351440	123495289	13	rs6561141	44295195	11	rs12281359	70417181
12	rs117242663	123495635	13	rs9533598	44296027	11	rs7106873	70425843
12	rs940906	123496927	13	rs9533599	44296248	11	rs4488220	70428477
12	rs7305511	123498253	13	rs76429286	44297268	11	rs11236898	70463510
12	rs12425850	123501972	13	rs10507520	44298794	11	rs11236899	70463655
12	rs12425009	123502044	13	rs7993358	44299027	11	rs561884	70502000
12	rs61955217	123504147	13	rs7994821	44299099	11	rs10501403	70552397
12	rs75338281	123507484	13	rs1493529	44299189	11	rs11237269	70560051
12	rs4759362	123508061	13	rs59987358	44299284	11	rs751690	70607985
12	rs77193970	123516317	13	rs923850	44299310	11	rs1207273	70716035
12	rs76573394	123516411	13	rs80001592	44299557	12	rs10848606	2140566
12	rs2844287	123517814	13	rs5019570	44299840	12	rs7971903	2145507
12	rs641760	123518866	13	rs17068821	44300156	12	rs7958309	2149038
12	rs74912794	123521867	13	rs9525828	44300256	12	rs9888368	2153616
12	rs520088	123522150	13	rs111648690	44301187	12	rs2108636	2159593
12	rs523682	123522523	13	rs6561143	44301443	12	rs740456	2168670
12	rs76678615	123523991	13	rs6561144	44301462	12	rs7972947	2170433
12	rs655293	123528405	13	rs77413508	44301704	12	rs2238015	2175413
12	rs11608811	123529056	13	rs17539229	44303296	12	rs740459	2250068
12	rs80326885	123532142	13	rs75988256	44303555	12	rs7304870	2273255
12	rs4148857	123539955	13	rs7316945	44304275	12	rs77635569	2273641
12	rs4148858	123540022	13	rs9525830	44304458	12	rs11062136	2273915
12	rs10773921	123541606	13	rs79657664	44304933	12	rs10848622	2274051
12	rs4148859	123542492	13	rs2029695	44305454	12	rs56197809	2274606
12	rs7957096	123544878	13	rs9595006	44305890	12	rs74062239	2274919
12	rs75200457	123545834	13	rs9533601	44305999	12	rs116863388	2275132
12	rs117379455	123545899	13	rs9567254	44306081	12	rs117224316	2275587
12	rs585522	123550813	13	rs4941470	44306586	12	rs2238043	2275663
12	rs484200	123550962	13	rs9567256	44307013	12	rs118017994	2276107
12	Chr12:122117299	123551346	13	rs17461155	44307042	12	rs76370001	2277259
12	rs28654629	123552741	13	rs12431115	44308676	12	rs74062240	2277318
12	rs77064381	123554082	13	rs17600057	44308790	12	rs2239014	2279117
12	rs35246351	123555116	13	rs117068145	44309386	12	rs11062137	2280672
12	rs117210587	123556647	13	rs17064994	44310404	12	rs10774029	2280807
12	rs7964876	123558705	13	rs9533602	44311636	12	rs11062138	2280942
12	rs1260294	123560289	13	rs9533603	44312190	12	rs11062139	2281369
12	rs596940	123560731	13	rs9525833	44312651	12	rs2370411	2281925
12	rs73230051	123561638	13	rs9525834	44312834	12	rs73050459	2282054
12	rs12810157	123565652	13	rs116894344	44313082	12	rs61909092	2282683
12	rs4148862	123568619	13	rs60989732	44314169	12	rs2239015	2283195
12	rs4148863	123569375	13	rs74920280	44314224	12	rs2239016	2283392
12	rs78299420	123571096	13	rs79758730	44315533	12	rs55884343	2283603

12	rs7967118	123571503	13	rs7139679	44317717	12	rs7298845	2284333
12	rs10848428	123572495	13	rs9525835	44318673	12	rs61909093	2284995
12	rs7312673	123572794	13	rs9525836	44318707	12	rs2238044	2285731
12	rs1727307	123575742	13	rs7986483	44319195	12	rs61909094	2285860
12	rs61955225	123575884	13	rs6561146	44319343	12	rs76116619	2285880
12	rs4148864	123580657	13	rs7327966	44319574	12	rs3794299	2285984
12	rs947800	123582855	13	rs17600078	44319625	12	rs7953823	2286022
12	rs117689661	123585705	13	rs7997792	44320785	12	rs74238853	2287097
12	rs1790094	123586688	13	rs766050	44321836	12	rs10848625	2288405
12	rs79769662	123586782	13	rs116895426	44322598	12	rs10848626	2288436
12	rs117065261	123587714	13	rs17461225	44323351	12	rs117361030	2288743
12	rs1617156	123590165	13	rs79469165	44324312	12	rs113019285	2288889
12	rs1790106	123591596	13	rs9595008	44324766	12	rs2159102	2288945
12	rs117860345	123591781	13	rs143124698	44325377	12	rs2239017	2289651
12	rs7132277	123593382	13	rs1411625	44325462	12	rs996950	2290338
12	rs7299943	123593485	13	rs9533606	44325785	12	rs10774030	2292690
12	rs73230058	123593899	13	rs7336604	44327719	12	rs7303140	2294006
12	rs75589918	123594402	13	rs4143229	44327799	12	rs758171	2294263
12	rs12423255	123595064	13	rs148856299	44328727	12	rs79255478	2294491
12	rs949143	123595163	13	rs11619756	44329004	12	rs79398153	2295156
12	rs4148865	123595749	13	rs9533608	44329066	12	rs116947827	2295261
12	rs1727326	123600086	13	rs9595009	44329883	12	rs11062145	2297353
12	rs11061615	123600864	13	rs1466705	44331185	12	rs10744559	2298298
12	rs1790121	123604492	13	rs115011334	44332730	12	rs2283287	2298724
12	rs1790122	123606739	13	rs9562510	44333748	12	rs2283288	2299048
12	rs116918050	123610753	13	rs73189971	44333851	12	rs2238045	2299823
12	rs76258382	123610989	13	rs1493522	44335216	12	rs2238047	2300008
12	rs56347904	123612452	13	rs113095637	44335483	12	rs111084453	2301189
12	rs79665790	123614832	13	rs117803643	44335959	12	rs78557449	2303269
12	rs76205535	123615869	13	rs117978017	44336272	12	rs2239018	2304357
12	rs1727294	123616514	13	rs9533609	44336779	12	rs2238049	2306128
12	rs200831962	123618362	13	rs78841243	44336797	12	rs2238050	2306707
12	rs11057165	123618368	13	rs7321106	44337569	12	rs2238051	2307175
12	rs1790116	123618544	13	rs11838826	44338776	12	rs2238052	2307296
12	rs1727316	123618603	13	rs17539382	44339125	12	rs79311680	2307869
12	rs6488865	123618894	13	rs77427342	44339251	12	rs73033311	2308797
12	rs1727317	123618942	13	rs9595012	44339427	12	rs10774032	2309393
12	rs78856743	123619648	13	rs17065044	44340042	12	rs10459125	2309864
12	rs1106240	123626982	13	rs9525838	44340106	12	rs2239020	2310452
12	rs1106241	123627016	13	rs9525839	44340683	12	rs2239021	2310529
12	rs11057167	123627491	13	rs9590786	44341387	12	rs2239022	2310551
12	rs77250160	123627905	13	rs9533610	44342409	12	rs2239023	2310616
12	rs77380554	123627972	13	rs9590787	44344300	12	rs2239024	2311313
12	rs1727302	123632930	13	rs1493523	44345614	12	rs1860056	2311836
12	rs74703625	123633057	13	rs9533611	44345667	12	rs2239025	2312282
12	rs1727305	123633382	13	rs73189985	44346523	12	rs2239027	2312301
12	rs1790090	123633424	13	rs75380973	44346586	12	rs10848627	2312408
12	rs949142	123634043	13	rs9533612	44346988	12	rs10848628	2312489

12	rs1790108	123636841	13	rs9525841	44347063	12	rs111349392	2312620
12	rs1716184	123637509	13	rs73189986	44347594	12	rs34640767	2312820
12	rs55863153	123637698	13	rs9533613	44347629	12	rs4765668	2312826
12	rs1790109	123637752	13	rs17065061	44348167	12	rs10848629	2312897
12	rs61187102	123639869	13	rs11840638	44348520	12	rs4765902	2312965
12	rs1727314	123640664	13	rs11619519	44349749	12	rs4765670	2313126
12	rs1727313	123640853	13	rs11147927	44351228	12	rs12423277	2314319
12	rs1051434	123641200	13	rs61960107	44351271	12	rs10774033	2314426
12	rs1716162	123642538	13	rs17539466	44352052	12	rs10848631	2314825
12	rs1047158	123644056	13	rs17600176	44352090	12	rs758172	2314997
12	rs2851439	123644420	13	rs77869381	44352172	12	rs76603827	2315540
12	rs10772993	123644714	13	rs9533614	44352837	12	rs758174	2315706
12	rs1260317	123645323	13	rs9595015	44354981	12	rs10848632	2315993
12	rs1051431	123645803	13	rs74878441	44356839	12	rs10848634	2316127
12	rs1260319	123645988	13	rs76348938	44359570	12	rs78907301	2316178
12	rs1727296	123647725	13	rs77354137	44361838	12	rs10848635	2316195
12	rs1727293	123648032	13	rs57130113	44362357	12	rs10848636	2316493
12	rs1716165	123648881	13	rs75651784	44363137	12	rs10848637	2316554
12	rs1615350	123650335	13	rs7993959	44364163	12	rs79475344	2317010
12	rs1716167	123651162	13	rs7993808	44364229	12	rs2238053	2317096
12	rs1716168	123651966	13	Chr13:43263124	44365124	12	rs11062156	2317523
12	rs34484751	123652527	13	rs9533615	44366602	12	rs77372211	2317604
12	rs117169430	123652848	13	rs117194765	44366879	12	rs2238054	2317644
12	rs1727291	123654498	13	rs9533616	44368071	12	rs11062157	2321820
12	rs1716176	123655467	13	rs9595018	44369477	12	rs1016388	2321868
12	rs1790098	123655481	13	rs7335504	44370271	12	rs12424245	2322513
12	rs1790099	123656533	13	rs7335893	44370429	12	rs200292772	2323144
12	rs4759367	123656544	13	rs7335939	44370498	12	rs139758774	2323145
12	rs1790100	123656725	13	rs73191903	44371107	12	rs2283290	2323621
12	rs2682427	123656809	13	rs9533617	44372671	12	rs11614275	2323846
12	rs2049114	123657338	13	rs58320322	44372887	12	rs7957545	2324042
12	rs12368208	123658014	13	rs9533618	44373113	12	rs2238055	2324407
12	rs1727309	123658258	13	rs17065076	44373509	12	rs11062158	2325978
12	rs2682428	123659380	13	rs76995318	44373851	12	rs2238056	2327944
12	rs1790123	123659542	13	rs9590791	44374650	12	rs72653432	2329572
12	rs1716173	123660721	13	rs1888303	44374981	12	rs11062161	2329970
12	rs10734898	123662408	13	rs1888302	44375330	12	rs10774034	2330458
12	rs1716164	123663660	13	rs17065082	44375529	12	rs12228890	2330997
12	rs3759113	123664438	13	rs11619557	44376241	12	rs3922316	2331144
12	rs1727301	123664514	13	rs11617352	44376328	12	rs2190771	2331192
12	rs2851447	123665113	13	rs17065084	44376934	12	rs10848642	2331572
12	rs1727306	123665695	13	rs1888301	44377001	12	rs11062162	2332104
12	rs11613128	123670792	13	rs61960111	44378186	12	rs2239028	2332210
12	rs184215819	123677291	13	rs1999823	44378738	12	rs76799633	2332371
12	rs141871291	123677810	13	rs73191914	44378806	12	rs4765904	2332393
12	rs79033960	123680935	13	rs73191915	44378824	12	rs1108221	2332795
12	rs1790095	123681222	13	rs73191917	44379088	12	rs1108222	2332856
12	rs1790126	123683563	13	rs9533622	44379822	12	rs1108074	2333484

12	rs74240768	123683976	13	rs9590793	44379896	12	rs1108073	2333638
12	rs61955270	123684003	13	rs9595019	44380677	12	rs1304749689	2333671
12	rs3759111	123687774	13	rs9533623	44380753	12	rs77893928	2333743
12	rs2695478	123689521	13	rs9525844	44381441	12	rs55802595	2333877
12	rs4759407	123689674	13	rs5803215	44382390	12	rs75729273	2334076
12	rs10846480	123691078	13	rs79375028	44383915	12	rs11062166	2334260
12	rs10846480	123691083	13	rs11618214	44384571	12	rs7312354	2334444
12	rs74240770	123691742	13	rs9525845	44385840	12	rs7296039	2334933
12	rs11057192	123693420	13	rs117493653	44386208	12	rs7977619	2335293
12	rs200046426	123693957	13	rs9567265	44386266	12	rs2239030	2335941
12	rs2682434	123694724	13	rs12583292	44386404	12	rs2239031	2336169
12	rs74240771	123696963	13	rs59825019	44386772	12	rs76314765	2336323
12	rs1727325	123698332	13	rs79807290	44386886	12	rs2239032	2337138
12	rs1790133	123698518	13	rs7330597	44387021	12	rs11613885	2337370
12	rs1790132	123698519	13	rs10454566	44387119	12	rs2283291	2337460
12	rs1727324	123698603	13	rs117137938	44387286	12	rs2239033	2337746
12	rs1727322	123699520	13	rs61960112	44387351	12	rs3794297	2338396
12	rs1716160	123700225	13	rs9595021	44387580	12	rs68089646	2338756
12	rs1727320	123700476	13	rs41385450	44387652	12	rs6489353	2338858
12	rs1716161	123700867	13	rs77982115	44388119	12	rs3829331	2339250
12	rs1790131	123701010	13	rs4942245	44389479	12	rs3794296	2339253
12	rs1630905	123705330	13	rs111406044	44390262	12	rs117433140	2340001
12	rs36121382	123705962	13	rs9525846	44390857	12	rs34382810	2340798
12	rs1626899	123708063	13	rs12866387	44391936	12	rs7965923	2341385
12	rs56104963	123709406	13	rs1445556	44393795	12	rs7303977	2342248
12	rs1790104	123709409	13	rs12583143	44394008	12	rs7135319	2342417
12	rs2851435	123712416	13	rs9533628	44394248	12	rs7305301	2342636
12	rs1716172	123712712	13	rs9533629	44394357	12	rs6489354	2343186
12	rs1727334	123714236	13	rs1813293	44394588	12	rs76086674	2343344
12	rs57676984	123714736	13	rs9567271	44394715	12	rs11609729	2343551
12	rs941306	123715113	13	rs981071	44394894	12	rs117924617	2344253
12	rs941305	123715266	13	rs17065087	44395100	12	rs117388872	2344560
12	rs1716171	123716376	13	rs117543541	44395232	12	rs769087	2344644
12	rs75037792	123716466	13	rs9595023	44395635	12	rs2007044	2344960
12	rs1716170	123716513	13	rs9533632	44396431	12	rs1006737	2345295
12	rs1716169	123716930	13	rs9533633	44396971	12	rs11062169	2345390
12	rs191114234	123717070	13	rs9533635	44397921	12	rs77013632	2345479
12	rs28362667	123717216	13	rs117831487	44398402	12	rs11610437	2345625
12	rs28362669	123717354	13	rs1353109	44398442	12	rs75269774	2345796
12	rs117107860	123717444	13	rs2172540	44399502	12	rs11614966	2346254
12	rs1727332	123718301	13	rs9533636	44400004	12	rs2159100	2346393
12	rs7304782	123719444	13	rs17600281	44400357	12	rs12315711	2346830
12	rs7139321	123719528	13	rs17065091	44400410	12	rs2239034	2347694
12	rs4759409	123719585	13	rs9533637	44400685	12	rs2239035	2349081
12	rs61953361	123719836	13	rs9533638	44401002	12	rs2239036	2349141
12	rs10772997	123719880	13	rs9533639	44401135	12	rs11612837	2349406
12	rs11057202	123720775	13	rs9525849	44401761	12	rs4765905	2349584
12	rs12825832	123720810	13	rs7988214	44401999	12	rs7975467	2350263

12	rs10772998	123721177	13	rs7988096	44402078	12	rs882195	2350401
12	rs73231928	123724325	13	rs9525850	44402100	12	rs882194	2350452
12	rs11057203	123725688	13	rs9525851	44402132	12	rs882193	2350620
12	rs1879379	123727443	13	rs7330760	44403088	12	rs80295761	2352835
12	rs12817892	123728702	13	rs7331149	44403269	12	rs77175673	2353407
12	rs10773000	123736084	13	rs9634665	44404368	12	rs78185961	2354263
12	rs2280424	123738494	13	Chr13:43302411	44404411	12	rs73033370	2354530
12	rs1568427	123738678	13	rs4942246	44404853	12	rs80071235	2354582
12	rs1568428	123738876	13	rs4942247	44405107	12	rs2370413	2354870
12	rs1879380	123739111	13	rs9525852	44405197	12	rs7967379	2355460
12	rs4759411	123740843	13	rs17065110	44405416	12	rs7297582	2355806
12	rs11554169	123741706	13	rs9567276	44405887	12	rs11608296	2356371
12	rs1969354	123741776	13	rs4942248	44406805	12	rs79446827	2356554
12	rs4759371	123742563	13	rs17065113	44407455	12	rs79976249	2356940
12	rs199777374	123742923	13	rs2325084	44407945	12	rs78661331	2358805
12	rs76327404	123743145	13	rs7982698	44409919	12	rs75892392	2359453
12	rs4759413	123743436	13	rs117266764	44409976	12	rs11615998	2360166
12	rs4759415	123743674	13	rs2325085	44410060	12	rs758170	2361460
12	rs10732573	123743883	13	rs2325086	44410088	12	rs11609090	2361915
12	rs10846489	123743927	13	rs12872943	44410553	12	rs11611154	2362006
12	rs2337934	123745359	13	rs9533646	44410669	12	rs7309075	2362116
12	rs6633	123745809	13	rs2084085	44411059	12	rs7138478	2362127
12	rs1801957	123745869	13	rs17065125	44411149	12	rs61907761	2362198
12	rs4759416	123746357	13	rs117740974	44411348	12	rs7139112	2362597
12	rs4759417	123746510	13	rs9567280	44411432	12	rs116644100	2362890
12	rs4759418	123746531	13	rs7332503	44411563	12	rs2239037	2363716
12	rs11057215	123746795	13	rs12429722	44412466	12	rs11612720	2363789
12	rs10773002	123746961	13	rs7320988	44413523	12	rs74347853	2364229
12	rs12828492	123747774	13	rs7328883	44415369	12	rs180703721	2364443
12	rs61231969	123748216	13	rs7330707	44415492	12	rs76139517	2364448
12	rs12146722	123749735	13	rs76331352	44415639	12	rs79164695	2364685
12	rs1402275	123750895	13	rs9567281	44415778	12	rs191226186	2365290
12	rs10846490	123750994	13	rs61209988	44416335	12	rs79472832	2365480
12	rs2030401	123751339	13	rs4941473	44416805	12	rs78308994	2365589
12	rs2271976	123751726	13	rs2325087	44417390	12	rs76836074	2365643
12	rs10083067	123752263	13	rs2875541	44417441	12	rs4765910	2365903
12	rs75529277	123752419	13	rs1822969	44418164	12	rs115124024	2365908
12	rs76514049	123752637	13	rs9533652	44419532	12	rs116642590	2367096
12	rs11057222	123752905	13	rs75849484	44420805	12	rs114364574	2367790
12	rs73231941	123753294	13	rs7339047	44421664	12	rs79091024	2368141
12	rs61953394	123753372	13	rs17653353	44421746	12	rs2370414	2368396
12	rs10846491	123753492	13	rs77216476	44422258	12	rs10774035	2368674
12	rs11057223	123755055	13	rs2065926	44422371	12	rs73033378	2368821
12	rs11057224	123755179	13	rs2065925	44422652	12	rs117703678	2370776
12	rs3759114	123757144	13	rs2065924	44422723	12	rs2283292	2371652
12	rs75759977	123757504	13	rs9525854	44422964	12	rs80191236	2373810
12	rs1109559	123757861	13	rs6561147	44423082	12	rs11615388	2374006
12	rs7953929	123758235	13	rs1337203	44423583	12	rs2239038	2374130

12	rs10846495	123760729	13	rs1337203	44423621	12	rs11608869	2374463
12	rs10444439	123766437	13	rs2325088	44424063	12	rs74767351	2374646
12	rs61953413	123766786	13	rs1466005	44424210	12	rs79122070	2375199
12	rs10846501	123768105	13	rs11620300	44424344	12	rs11616036	2375612
12	rs11057239	123768246	13	rs77189267	44424499	12	rs1006690	2375636
12	rs11057240	123768264	13	rs1808097	44424766	12	rs2159101	2375687
12	rs10846502	123768614	13	rs12875768	44428305	12	rs7485359	2375793
12	rs74354194	123770140	13	rs118196604	44431660	12	rs77205646	2376027
12	rs61953417	123771381	13	rs9562516	44431738	12	rs116229134	2376168
12	rs7974099	123771475	13	rs12874590	44433363	12	rs78155648	2376292
12	rs10773003	123775127	13	rs12874535	44433565	12	rs80284141	2376294
12	rs80200546	123775802	13	rs1562215	44433567	12	rs75548797	2376651
12	rs79015132	123776151	13	rs3088362	44433630	12	rs74393821	2376815
12	rs181914282	123776159	13	rs1562216	44433700	12	rs1476733	2376900
12	rs61953419	123778469	13	rs117389460	44434401	12	rs79399264	2377952
12	rs3088303	123779489	13	rs9533659	44434663	12	rs116014402	2378234
12	rs10846507	123786492	13	rs9533660	44434735	12	rs11614796	2379100
12	rs58537268	123787505	13	rs9533661	44434748	12	rs11609165	2379874
12	rs60755632	123787558	13	rs74845940	44435494	12	rs11615807	2380232
12	rs11057250	123787811	13	rs77727531	44435953	12	rs80106079	2381056
12	rs12317452	123788246	13	rs9533662	44435988	12	rs79411638	2381569
12	rs78862746	123789374	13	rs9562517	44436444	12	rs12298346	2381910
12	rs10773005	123790007	13	rs9525856	44437052	12	rs114723538	2382549
12	rs11057251	123791227	13	rs9567289	44437104	12	rs4488257	2382964
12	rs11057252	123791454	13	rs7989353	44438957	12	rs2370415	2383050
12	rs80151828	123791858	13	rs7989702	44439150	12	rs117565726	2383682
12	rs74240781	123792701	13	rs12865116	44440441	12	rs2238057	2384005
12	rs10846509	123792848	13	rs9533664	44440527	12	rs76777168	2384042
12	rs57356592	123793660	13	rs9525858	44441221	12	rs74992059	2384150
12	rs3825141	123794104	13	rs618179	44442033	12	rs74411248	2384223
12	rs2277343	123794450	13	rs1867629	44442037	12	rs79426448	2384357
12	rs10846510	123794557	13	rs7322781	44442238	12	rs77558515	2384432
12	rs11057254	123794581	13	rs117153101	44442365	12	rs7969407	2384768
12	rs76466196	123795580	13	rs17571505	44442374	12	rs61907778	2384815
12	rs80336138	123795759	13	rs4942252	44443039	12	rs77759718	2384894
12	rs4759375	123796238	13	rs17065161	44443723	12	rs7979604	2385018
12	rs4759377	123796849	13	rs11617551	44443977	12	rs76173403	2385283
12	rs10082867	123796997	13	rs2121030	44444574	12	rs76721755	2386415
12	rs10846513	123798074	13	rs17065164	44444706	12	rs75705537	2386772
12	rs74690356	123798352	13	rs116877091	44444862	12	rs10774036	2386948
12	rs77190493	123799636	13	rs17065166	44444990	12	rs10744560	2387099
12	rs61751327	123799938	13	rs7318147	44445153	12	rs79189017	2387245
12	rs6488868	123799974	13	rs7319725	44445421	12	rs118004017	2387701
12	rs10846514	123800376	13	rs7334644	44445509	12	rs75728817	2388507
12	rs10773007	123800752	13	rs7334812	44445538	12	rs114567313	2388653
12	rs10846515	123801672	13	rs12428350	44446161	12	rs7972103	2388720
12	rs61751328	123801856	13	rs9533665	44446473	12	rs34335441	2389220
12	rs57416942	123801940	13	rs12853286	44446817	12	rs77577013	2390132

12	rs10846516	123803469	13	rs118030678	44446957	12	rs78184324	2391325
12	rs59295450	123803880	13	rs9533666	44447183	12	rs77704787	2391412
12	rs12366872	123804721	13	rs9567293	44447243	12	rs74630743	2391544
12	rs61760909	123805261	13	rs9533667	44448132	12	rs79832477	2391562
12	rs1060105	123806219	13	rs9533668	44448522	12	rs115500284	2394025
12	rs74240784	123806666	13	rs2305100	44448934	12	rs76408048	2394434
12	rs77317455	123806965	13	rs12875052	44449368	12	rs78525694	2394494
12	rs77916750	123807018	13	rs118066719	44449900	12	rs78161460	2394704
12	rs11057266	123807545	13	rs9533670	44450122	12	rs12311439	2394781
12	rs10744152	123809798	13	rs2325089	44450222	12	rs78683530	2394935
12	rs117843331	123809845	13	rs2184882	44450346	12	rs117311450	2395626
12	rs7304190	123810375	13	rs9533671	44450594	12	rs11610448	2396153
12	rs12304248	123810425	13	rs77007175	44450794	12	Chr12:2267094	2396833
12	rs12312675	123810462	13	rs58267485	44451477	12	rs1984637	2397108
12	rs11057272	123812197	13	rs7327496	44451539	12	rs115724978	2397793
12	rs10846517	123812611	13	rs4942254	44451820	12	rs116698755	2399295
12	rs28419092	123812629	13	rs4942255	44451844	12	rs117067220	2399399
12	rs10846518	123812763	13	rs67973257	44452229	12	rs11614494	2399937
12	rs10846519	123812787	13	rs12868462	44453480	12	rs75739143	2401270
12	rs7297649	123813071	13	rs3816311	44453783	12	rs79899792	2401377
12	rs11057273	123814466	13	rs3816312	44453873	12	rs11615645	2402092
12	rs7486351	123815939	13	rs2275252	44454374	12	rs114241316	2402144
12	rs200166433	123816018	13	rs3818818	44454455	12	rs76623174	2402241
12	rs12316131	123817796	13	rs9525863	44454705	12	rs1024582	2402246
12	rs10773009	123818007	13	rs66688207	44454734	12	rs116298939	2402318
12	rs7484644	123818424	13	rs34414396	44455233	12	rs56177308	2402990
12	rs11057276	123820397	13	rs182325132	44455919	12	rs7310491	2404022
12	rs7972811	123820780	13	rs9567299	44456969	12	rs76540614	2405146
12	rs7486223	123821234	13	rs11616803	44457109	12	rs11615601	2405598
12	rs11057278	123821609	13	rs11619043	44457210	12	rs76719316	2406103
12	rs7977563	123822358	13	rs9525864	44457611	12	rs74500489	2406703
12	rs6488870	123822560	13	rs3764147	44457925	12	rs113258895	2407139
12	rs6488871	123822639	13	rs9533673	44457984	12	rs4298967	2408194
12	rs7980687	123822711	13	rs73174269	44458803	12	rs73033382	2408635
12	rs61953459	123823450	13	rs895266	44461179	12	rs16929233	2411198
12	rs11830103	123823546	13	rs9533675	44461230	12	rs1860002	2413803
12	rs10773010	123823685	13	rs9533676	44461618	12	rs7966042	2414353
12	rs112160305	123824516	13	rs61959998	44462501	12	rs117839053	2415584
12	rs67624109	123824635	13	rs9533678	44463409	12	rs117639423	2416680
12	rs10846521	123825157	13	rs9525867	44463557	12	rs7966811	2416808
12	rs7312145	123825339	13	rs9525868	44463697	12	rs75726475	2416997
12	rs7315453	123825389	13	rs12428432	44464043	12	rs118065562	2419083
12	rs76725288	123827106	13	rs7989857	44464090	12	rs11062183	2419245
12	rs11057282	123828556	13	rs1445557	44464827	12	rs2190732	2419382
12	rs7955367	123828792	13	rs9533681	44465033	12	rs10848645	2420244
12	rs7973878	123828826	13	rs9533682	44465405	12	rs4765914	2420377
12	rs12298826	123829028	13	rs7992315	44465879	12	rs10774037	2420526
12	rs34997336	123829116	13	rs9567302	44466356	12	rs2283293	2420607

12	rs11057284	123830692	13	rs117297262	44467157	12	rs2108570	2421588
12	rs7298909	123830939	13	rs9533684	44468177	12	rs2370419	2423857
12	rs10846523	123832746	13	rs9533685	44468331	12	rs2283301	2446806
12	rs11057287	123832854	13	rs1811464	44469290	12	rs994901	2533178
12	rs56116847	123835233	13	rs4101376	44469835	12	rs2239073	2538500
12	rs78344454	123835678	13	rs187836497	44471277	12	rs10491965	2551368
12	rs9300256	123837375	13	rs2121037	44471317	12	rs10774048	2562541
12	rs34896222	123838107	13	rs9562521	44471470	12	rs4765937	2570535
12	rs9795593	123839052	13	rs9567305	44471656	12	rs10848660	2579615
12	rs9739008	123840858	13	rs146164081	44471983	12	rs7306916	2601363
12	rs9739565	123840866	13	rs77296273	44472138	12	rs4765950	2611407
12	rs9739171	123840994	13	rs17462017	44472235	12	rs12231350	2635190
12	rs9669169	123841717	13	rs7321596	44472255	15	rs1359003	42656939
12	rs9669295	123842670	13	rs17571812	44472462	15	rs1359004	42657055
12	rs10773014	123842897	13	rs75573572	44473404	15	rs3850774	42658157
12	rs9668827	123843017	13	rs55658348	44473419	15	rs2407707	42659350
12	rs61955083	123843379	13	rs8002861	44474517	15	rs4924674	42668498
12	rs28683528	123845351	13	rs1373904	44475398	15	rs57134130	42669574
12	rs28553365	123847841	13	rs7318538	44475424	15	rs57347762	42671558
12	rs28660661	123848455	13	rs1373903	44475514	15	rs28364390	42677173
12	rs61955086	123848987	13	rs12428112	44475821	15	Chr15:40465251	42677959
12	rs28627651	123849051	13	rs12428314	44476011	15	rs7168709	42679306
12	rs117135286	123849212	13	rs2325070	44476744	15	rs28364405	42679414
12	rs60754073	123849272	13	rs12869355	44478193	15	rs751572	42679789
12	rs28646185	123849629	13	rs12869521	44478346	15	rs4924675	42681057
12	rs28759130	123849774	13	rs7994883	44478805	15	rs1801449	42681199
12	rs28376696	123849863	13	rs7995004	44478820	15	rs16973226	42681609
12	rs28594416	123849921	13	rs78954910	44478876	15	rs2412709	42681930
12	rs28418709	123850168	13	rs10507522	44479000	15	rs28364428	42682705
12	rs28517382	123850197	13	rs9567308	44479738	15	rs28364433	42683630
12	rs78092282	123850260	13	rs77013804	44479822	15	rs2412710	42683787
12	rs61955088	123850541	13	rs8001833	44480132	15	rs202060806	42684389
12	rs117718416	123852477	13	rs9533689	44480413	15	rs12439003	42684455
12	rs28414347	123860025	13	rs9533690	44480598	15	rs12324205	42684680
12	rs28786830	123860472	13	rs17600603	44481812	15	rs28364447	42685454
12	rs28458312	123863265	13	rs7992088	44482005	15	rs17764737	42685634
12	rs28690392	123863576	13	rs9525873	44482204	15	rs8024113	42686838
12	rs28592876	123866429	13	rs9533691	44482208	15	rs28364458	42687263
12	rs182270339	123867106	13	rs17600623	44482517	15	rs7168121	42687554
12	rs116904769	123867183	13	rs7999434	44482961	15	rs3115877	42688772
12	rs28613486	123867268	13	rs12867732	44483084	15	rs28364469	42689318
12	rs28636834	123871070	13	rs73467552	44483215	15	rs3743003	42691664
12	rs28475177	123872037	13	rs68089046	44483593	15	rs148044781	42694089
12	rs28533432	123873242	13	rs7983109	44484351	15	rs28364492	42694568
12	rs28660993	123875394	13	rs7983279	44484409	15	rs2241827	42694944
12	rs28681105	123875525	13	rs6561151	44484706	15	rs7163986	42696158
12	rs28501453	123877312	13	rs9316058	44485076	15	rs111814065	42696239
12	rs28820735	123878507	13	rs17462156	44485551	15	rs2412711	42697231

12	rs28655516	123879975	13	rs9567309	44485618	15	rs28364514	42698753
12	rs28410096	123880862	13	rs9533694	44486382	15	rs3115882	42701203
12	rs28372579	123881176	13	rs9316059	44486789	15	rs3115883	42701688
12	rs28546238	123882977	13	rs76959557	44486955	15	rs181742437	42703210
12	rs28532037	123883406	13	rs117662940	44487206	15	rs116852089	42703356
12	rs28587386	123883853	13	rs7995356	44487578	15	rs3115884	42703406
12	rs28377694	123884070	13	rs7997017	44487611	15	rs3098423	42704105
12	rs28472704	123887369	13	rs920982	44488692	15	rs28364548	42704914
12	rs144550004	123890062	13	rs74347409	44489181	15	rs28364550	42705007
12	rs73216931	123891209	13	rs7322107	44489602	15	rs28364552	42705191
12	rs28406193	123891865	13	rs11147932	44489674	15	rs28364553	42705342
12	rs73216932	123893322	13	rs7322436	44489710	15	rs28364554	42705371
12	rs28694725	123893645	13	rs61960013	44490181	15	rs12594633	42705464
12	rs147353607	123893663	13	rs80252743	44490288	15	rs180869563	42707912
12	rs74968466	123894014	13	rs74516505	44490562	15	rs62022300	42709998
12	rs28573238	123894098	13	rs4942259	44490577	15	rs17708630	42710177
12	rs56309603	123895053	13	rs9525874	44491229	15	rs61071861	42710219
12	rs28664930	123895197	13	rs9595036	44491355	15	rs62022303	42713462
12	rs28539053	123895263	13	rs117630415	44492074	15	rs117272458	42714028
12	rs28569885	123897177	13	rs583271	44493527	15	rs7169521	42715944
12	rs2137558	123899505	13	rs9533697	44493863	15	rs3742999	42716581
12	rs1660	123900107	13	rs9567314	44493872	15	rs17765354	42718917
12	rs1662	123900136	13	rs78218382	44494793	15	rs11544099	42720288
12	rs28713448	123901480	13	rs77332730	44494921	15	rs11639355	42723407
12	rs71456795	123902892	13	rs79124647	44495917	15	rs62022307	42726610
12	rs28706696	123908126	13	rs636086	44497715	15	rs28602748	42726879
12	rs71456796	123908570	13	rs9595037	44498085	15	rs28726153	42726896
14	rs1152794	99711953	13	rs7319445	44498237	15	rs35896246	42727621
14	rs2236281	104000518	13	rs2028812	44499013	15	rs3742997	42729286
14	rs7161305	104002234	13	rs823880	44500205	15	rs12442046	42730193
14	rs9324063	104006693	13	rs76308253	44501454	15	rs3742996	42740369
14	rs7140558	104018651	13	rs9533700	44501582	15	rs8026817	42741551
14	rs4906335	104021141	13	rs1596760	44503677	15	rs8027283	42741636
14	rs7148456	104028270	13	rs118089263	44504077	15	rs34792942	42742435
14	rs2296482	104029819	13	rs189369295	44504237	15	rs12101559	42742464
14	rs12889403	104034746	13	rs78982232	44504869	15	rs12440118	42744094
14	rs7140568	104041374	13	rs115387512	44506403	15	rs2899050	42745702
14	rs8017993	104047734	13	rs622920	44506671	15	rs17709180	42746391
14	rs2403197	104053764	13	rs9567319	44507089	15	rs76008547	42747985
14	rs8021368	104074810	13	rs590566	44507533	15	rs57734638	42749013
14	rs729438	104092789	13	rs78018040	44507812	15	rs16973261	42749023
14	rs709400	104149475	13	rs617412	44507962	15	rs17709287	42751917
14	rs12432907	104168378	13	rs629531	44508363	15	rs4924687	42860591
15	rs2305645	40595565	13	rs115086777	44509139	15	rs11632169	42903115
15	rs1869901	40595627	13	rs117705635	44509365	15	rs4447398	42904904
15	rs3784397	40596844	13	rs117104581	44509510	15	rs12594951	42934631
15	rs3784399	40598294	13	rs17600693	44509519	15	rs17774047	43013226
15	rs1123487	40598941	13	rs8000599	44509678	15	rs12594483	43021986

15	rs11071612	61834227	13	rs652145	44509833	15	rs11070378	43076324
15	rs7174831	61838454	13	rs2875537	44510432	15	rs7167802	43116906
15	rs4775413	61840103	13	rs61960016	44510451	15	rs3825877	85174631
15	rs4775419	61860911	13	rs61960018	44510940	15	rs510875	85186637
15	rs2414716	61861402	13	rs669352	44511008	15	rs17598561	85198606
15	rs10163060	61861540	13	rs641334	44511123	15	rs309430	85203070
15	rs2414717	61861985	13	rs17065212	44511435	15	rs10438428	85330745
15	rs8042151	61862802	13	rs111602786	44511626	15	rs1429444	85379278
15	rs8042070	61862996	13	rs117479357	44511902	15	rs182517	85389631
15	rs2414718	61863133	13	rs17572067	44512464	15	rs306207	85390346
15	rs17270962	61864076	13	rs7998410	44513260	15	rs16974567	85397374
15	rs2414719	61869851	13	rs12872902	44514022	15	rs9944229	85404969
15	rs12595508	61870977	13	rs17462313	44514422	15	rs12904777	85415745
15	rs7496758	61882877	13	rs17600726	44514431	15	rs8037320	85437286
15	rs17270983	61887768	13	rs9567321	44514471	15	rs4842999	85446353
15	rs16944389	61895514	13	rs823888	44514974	15	rs16974600	85451378
15	rs7182531	61900183	13	rs592603	44515235	15	rs17537076	85464498
15	rs7168305	61903224	13	rs895265	44515421	15	rs3825875	85467207
15	rs7175667	61904270	13	rs1337200	44515497	15	rs2242046	85478729
15	rs1971791	70576138	13	rs2121032	44515645	15	rs4247411	85492288
15	rs8034191	78806023	13	rs2325074	44515764	15	rs7170598	85533556
15	rs10519203	78814046	13	rs9533703	44516195	15	rs971804	85544381
15	rs11858230	78835552	13	rs79530839	44516226	15	rs12900736	85558140
15	rs2036527	78851615	13	rs1108276	44516427	15	rs17611075	85568939
15	rs17486278	78867482	13	rs656584	44516605	16	rs8061640	9702388
15	rs637137	78873976	13	rs9525876	44516825	16	rs13333400	9705361
15	rs481134	78877563	13	rs9533704	44516967	16	rs8051282	9709527
15	rs16969968	78882925	13	rs1415367	44517780	16	rs1990573	9713688
15	rs518425	78883813	13	rs1337202	44517895	16	rs977540	9724750
15	rs514743	78884227	13	rs79507410	44517957	16	rs11646985	9755469
15	rs12910984	78891627	13	rs2121031	44518147	16	rs17568440	9755481
15	rs1051730	78894339	13	rs2166299	44518334	16	rs11642164	9757306
15	rs12914385	78898723	13	rs7997200	44518399	16	rs11645816	9772106
15	rs8042374	78908032	13	rs9533705	44518595	16	rs3968868	9772529
15	rs3743074	78909480	13	rs9533706	44518821	16	rs4063490	9772573
15	rs6495309	78915245	13	rs9533707	44518967	16	rs10852232	9780993
15	rs950776	78926018	13	rs9533708	44519031	16	rs1476954	9793382
15	rs2292188	84700783	13	rs9533709	44519156	16	rs7203936	9798986
15	rs10520569	84729389	13	rs17572130	44519281	16	rs9939321	9807616
15	rs11638297	84782417	13	rs9562524	44519491	16	rs2215919	9811679
15	rs12911223	84831671	13	rs9533710	44519557	16	rs9925720	9842752
15	rs12591311	85087259	13	rs9525878	44519733	16	rs1014531	9855794
15	rs11638788	85091287	13	rs9525879	44519875	16	rs7190785	9873465
15	rs1981458	91416605	13	rs9567322	44519947	16	rs1544604	9887244
15	rs111239956	91417850	13	rs9525880	44520348	16	rs8050843	9930580
15	rs59596774	91418237	13	rs17065230	44520460	16	rs2215718	9938796
15	rs4526996	91418591	13	rs9533711	44520500	16	rs1548808	9966207
15	rs60994696	91418723	13	rs9525881	44520683	16	rs9931155	9984523

15	rs73489557	91419490	13	rs17572193	44520796	16	rs4782039	10006967
15	rs12917264	91420552	13	rs73175754	44520953	16	rs13331097	10052349
15	rs2071410	91420940	13	rs74844209	44520983	16	rs11642346	10058574
15	rs1573643	91420973	13	rs17600927	44521235	16	rs884918	10059826
15	rs1573644	91421283	13	rs76243355	44521306	16	rs3859123	10070895
15	rs6224	91423543	13	rs66992708	44521325	16	rs3848326	10087961
15	rs75493298	91423825	13	rs9525882	44521853	16	rs844395	10102906
15	rs6226	91424574	13	rs2065923	44522053	16	rs837694	10110040
15	rs6227	91425232	13	rs1596758	44522183	16	rs2315514	10110285
15	rs4702	91426560	13	rs9525883	44523309	16	rs3848333	10117555
15	rs79865852	91426816	13	rs4537909	44523508	16	rs1448258	10151357
15	rs76642265	91428188	13	rs9533713	44523654	16	rs11074568	10176042
15	rs2071382	91428197	13	rs113491984	44523719	17	rs231518	41961451
15	rs11539637	91428290	13	rs1822965	44523937	17	rs231492	41978756
15	rs7177338	91428636	13	rs9525884	44524160	17	rs231485	41982012
15	rs1894400	91428955	13	rs823886	44524613	17	rs1731888	41992617
16	rs1544604	9887244	13	rs9533714	44524884	17	rs1731896	42014796
16	rs8050843	9930580	13	rs9533716	44526008	17	rs231471	42019494
16	rs2215718	9938796	13	rs117828108	44527226	17	rs8079623	42027330
16	rs1548808	9966207	13	rs9567323	44528110	17	rs231461	42033043
16	rs8060141	13735912	13	rs7331414	44528130	17	rs2700830	42042350
16	rs12597362	13753349	13	rs7338524	44528593	17	rs1642599	42045138
16	rs11646118	29926471	13	rs7139721	44529173	17	rs9907468	42052089
16	rs4609871	29932064	13	rs7338872	44529281	17	rs918242	42054739
16	rs4283241	29988349	13	rs7140117	44529348	17	rs9895585	42064987
16	rs4787491	30015337	13	rs7338748	44529443	17	rs16940462	42108366
16	rs9783783	30078492	13	rs7320945	44529700	17	rs228762	42138392
16	rs6565174	30111904	13	rs7320768	44529726	17	rs850856	42157739
16	rs7698	30125800	13	rs9567324	44529987	17	rs228757	42164885
16	rs11865086	30130493	13	rs7322264	44530336	17	rs400460	42182770
16	rs11641947	58669908	13	rs7326990	44530686	17	rs228765	42186422
16	rs12446487	58671815	13	rs59479159	44530999	17	rs8065686	42193897
16	rs12447862	58672763	13	rs7334373	44532048	17	rs227579	42223134
16	rs67742851	58678335	13	rs7334745	44532154	17	rs2071167	42287519
16	rs12325245	58681393	13	rs7334991	44532291	17	rs45519733	42327850
16	rs154440	58682616	13	rs9533720	44532759	17	rs1476512	42330122
16	rs9922575	58682738	13	rs61469179	44532926	17	rs2074108	42336149
16	rs56071820	67712018	13	rs9525887	44533189	17	rs5035	42338998
16	rs7202185	67714560	13	rs7318548	44533368	17	rs4793092	42400480
16	rs191901013	67725421	13	rs9567328	44533647	17	rs3785817	42423665
16	rs12927959	67727069	13	rs9525889	44533844	17	rs5848	42430244
16	rs78563448	67731204	13	rs9533722	44534486	18	rs17069845	60011615
16	rs146965632	67737337	13	rs77156547	44534657	18	rs6567272	60027171
16	rs56047901	67742326	13	rs1373905	44534727	18	rs1805034	60027241
16	rs74900413	67743424	13	rs7331538	44535434	18	rs8092336	60036083
16	rs62059344	67747306	13	rs9533723	44535509	18	rs4426449	60042817
16	rs4474673	67758778	13	rs9533724	44535571	18	rs17069906	60048394
16	rs73591936	67761825	13	rs9533725	44535669	18	rs2957128	60060735

16	rs112526325	67761917	13	rs9567330	44536248	18	rs3017368	60067128
16	rs76851342	67762803	13	rs9525892	44536619	18	rs7227275	60067241
16	rs8051632	67767901	13	rs9525893	44536935	18	rs2957145	60076799
16	rs72790357	67769802	13	rs80266230	44537676	18	rs8094440	60092936
16	rs56845922	67770828	13	rs9525894	44537772	18	rs17070008	60115718
16	rs9925393	67780829	13	rs151319278	44537888	18	rs17070018	60121794
16	rs73591955	67782568	13	rs9525895	44538352	18	rs12954590	60155750
16	rs76118107	67786611	13	rs9567331	44538413	18	rs599323	60165701
16	rs10775302	67792439	13	rs9567332	44538710	18	rs2032396	60217134
16	rs187399063	67792741	13	rs61960023	44538830	18	rs1052315	60253761
16	rs117024179	67795150	13	rs9533726	44538922	18	rs8095794	60267933
16	rs6499141	67797134	13	rs9525896	44539105	18	rs188624	60322949
16	rs186039084	67797575	13	rs1445555	44539361	19	rs7257072	19267990
16	rs16957524	67798005	13	rs9533727	44539848	19	rs72997195	19301232
16	rs73591968	67803063	13	rs9562527	44540696	19	rs78454476	19301296
16	rs28580313	67806351	13	rs12431170	44542742	19	rs62135502	19303185
16	rs118016432	67806696	13	rs1530489	44543013	19	rs79003009	19303284
16	rs117829552	67814653	13	rs1530488	44543024	19	rs1050483	19303802
16	rs8058835	67814865	13	rs1530487	44543106	19	rs2283626	19304965
16	rs117588620	67815040	13	rs111584242	44543173	19	rs72997200	19307797
16	rs77789640	67824465	13	rs3794427	44544182	19	rs10424365	19310527
16	rs8057184	67827247	13	rs117487964	44544275	19	rs12610057	19315180
16	rs75276053	67827734	13	rs113081917	44544300	19	rs12982158	19316107
16	rs9928531	67833171	13	rs3794431	44544511	19	rs56007657	19316338
16	rs35652982	67834340	13	rs113399414	44544665	19	rs79573461	19316458
16	rs17619927	67834585	13	rs3794434	44544944	19	rs113571327	19316827
16	rs12448923	67839128	13	rs3794435	44544983	19	rs10412238	19318158
16	rs7199443	67841129	13	rs3794436	44545134	19	rs28618105	19318465
16	rs8051653	67848515	13	rs3794438	44545226	19	rs73537466	19318794
16	rs35316276	67850700	13	rs8000271	44550398	19	rs12461092	19319484
16	rs2008173	67856240	13	rs4942260	44550570	19	rs80290080	19324258
16	rs62620177	67860637	13	rs4942261	44551078	19	rs190534855	19324315
16	rs2292316	67861134	13	rs4942262	44551250	19	rs7250569	19324443
16	rs34693072	67861501	13	rs9562529	44551293	19	rs62135543	19324806
16	rs3743733	67863451	13	rs9562530	44551345	19	rs140514916	19325035
16	rs8052287	67865073	13	rs7325401	44551531	19	rs3761077	19325963
16	rs8052191	67865287	13	rs78367309	44552853	19	rs2228603	19329924
16	rs11558533	67865937	13	rs79587940	44552880	19	rs4808187	19330213
16	rs73593810	67866783	13	rs76481363	44553677	19	rs11667950	19330553
16	rs6499143	67874264	13	rs75874478	44554086	19	rs77720510	19330832
16	rs78033862	67874701	13	rs79249026	44554099	19	rs112254184	19331562
16	rs3809630	67879400	13	rs77316503	44555023	19	rs112014012	19331858
16	rs1113232	67879827	13	rs2121036	44555182	19	rs2238672	19335083
16	rs75957286	67881989	13	rs117584167	44556403	19	rs2238673	19335307
16	rs57721459	67884111	13	rs9567339	44556691	19	rs2238674	19335751
16	rs111402573	67884411	13	rs73177327	44557667	19	rs2238675	19336608
16	rs7198357	67884619	13	rs78536749	44557726	19	rs28409009	19340265
16	rs12930280	67891074	13	rs1822970	44559045	19	rs28446016	19340407

16	rs72790367	67891658	13	rs3764144	44559147	19	rs1859055	19341160
16	rs72790368	67892362	13	rs17601039	44559850	19	rs11668677	19341446
16	rs17687442	67894626	13	rs4942263	44560970	19	rs28510494	19341856
16	rs1124324	67897487	13	rs7996812	44561629	19	rs112823387	19342455
16	rs117878082	67898492	13	rs77984903	44561815	19	rs10403859	19342911
16	rs1123072	67898797	13	rs77287072	44561875	19	rs75072017	19344059
16	rs13334918	67901343	13	rs12867740	44562537	19	rs34673002	19344525
16	rs2271293	67902070	13	rs117397946	44563029	19	rs11672216	19344579
16	rs2271294	67902326	13	rs17572338	44563948	19	rs10424369	19345356
16	rs117600022	67903296	13	rs7326184	44564400	19	rs2228600	19345802
16	rs76649027	67905994	13	rs17653933	44565349	19	rs77927505	19347367
16	rs56374641	67908713	13	rs17462548	44568727	19	rs17215684	19349980
16	rs34132524	67909150	13	rs9533737	44568888	19	rs72999016	19350103
16	rs8060686	67911517	13	rs17462562	44570770	19	rs13345554	19351353
16	rs34607252	67917958	13	rs117735162	44571012	19	rs2229851	19351431
16	rs73594554	67920049	13	rs80207445	44571944	19	rs73922843	19351761
16	rs10468274	67922342	13	rs4942264	44574282	19	rs75235904	19353979
16	rs76521039	67922697	13	rs79429131	44574747	19	rs10402294	19354937
16	rs73594556	67925435	13	rs117839563	44575060	19	rs10408021	19355908
16	rs7196789	67927124	13	rs7490086	44577770	19	rs4808931	19358672
16	rs34298659	67927697	13	rs7334868	53395092	19	rs10423874	19359500
16	rs16942887	67928042	13	rs10162284	53397648	19	rs1064395	19361735
16	rs6499145	67929601	13	rs649523	53440459	19	rs10401714	19363630
16	rs78572164	67931112	13	rs4886021	53466127	19	rs4808933	19363812
16	rs118106500	67931383	13	rs17052423	53478139	19	rs72999030	19364240
16	rs72790373	67934780	13	rs7333164	53483062	19	rs72999033	19366632
16	rs117312671	67936863	13	rs7987314	53485153	19	rs17216041	19366643
16	rs12599580	67950613	13	rs732949	53517884	19	rs56044734	19366927
16	rs76944074	67951827	13	rs7337967	53519045	19	rs56278919	19367190
16	rs73594581	67955554	13	rs9536326	53550095	19	rs55796368	19367313
16	rs79327462	67958058	13	rs1555559	53566722	19	rs55762233	19367319
16	rs117767794	67963655	13	rs2298229	53602970	19	rs55765017	19368264
16	rs1134760	67964203	13	rs34067666	53617309	19	rs56144632	19368310
16	rs7187289	67967878	13	rs2806975	53662082	19	rs75590738	19368482
16	rs14178	67969531	13	rs12867491	53696774	19	rs118043674	19370021
16	rs20549	67969930	14	rs2021937	42113905	19	rs4808934	19370917
16	rs11574514	67971380	14	rs4304945	42220920	19	rs56135441	19371370
16	rs5923	67973953	14	rs862541	42281445	19	rs7257875	19372150
16	rs4986970	67976320	14	rs4900098	42309260	19	rs10422893	19372185
16	rs1109166	67977382	14	rs1951874	42317467	19	rs141026619	19372723
16	rs17851876	67979423	14	rs12882576	42319515	19	rs74695215	19373944
16	rs76074266	67979541	14	rs1957861	42326572	19	rs8105094	19374061
16	rs11860125	67980969	14	rs17092293	42337898	19	rs8105984	19374068
16	rs117213038	67981697	14	rs17093516	64480358	19	rs35854118	19375372
16	rs11542821	67984589	14	rs4566057	64506541	19	rs78538967	19375822
16	rs116856853	67984804	14	rs11158526	64557407	19	rs2074298	19377716
16	rs2292318	67985706	14	rs10137972	64557734	19	rs117877390	19378416
16	rs118000276	67988447	14	rs2781377	64560092	19	rs998732	19378671

16	rs9928014	67991427	14	rs8007972	64564527	19	rs58542926	19379549
16	rs78818722	67991972	14	rs36021513	64588825	19	rs72999063	19380596
16	rs3785098	67993643	14	rs34944385	64588837	19	rs2074299	19380646
16	rs3785100	67997920	14	rs1268656	64647646	19	rs72999068	19380685
16	rs78335263	67998187	14	rs12434245	64691853	19	rs12974359	19381439
16	rs28655136	68001447	14	rs17751556	64884106	19	rs2074301	19381715
16	rs147329698	68001555	14	rs3818239	64914537	19	rs2074303	19381755
16	rs73596542	68004558	14	rs8017803	75108422	19	rs116904583	19382215
16	rs28668419	68010195	14	rs10047892	75111346	19	rs55822665	19383298
16	rs28490940	68010262	14	rs12323392	75115673	19	rs10419245	19383755
16	rs72790380	68012694	14	rs17101733	75123026	19	rs78640921	19384914
16	rs255048	68012849	14	rs7153812	75173490	19	rs11669578	19385080
16	rs11643888	68013346	14	rs2287400	75182937	19	rs735273	19385411
16	rs255049	68013471	14	rs11844957	75224651	19	rs8103496	19386015
16	rs255054	68017356	14	rs2111705	75261641	19	rs74182631	19386738
16	rs1107767	68020063	14	rs11159103	75271354	19	rs757346	19387185
16	rs78539049	68021125	14	rs12588415	75278211	19	rs2010506	19387356
16	rs1133090	68021468	14	rs10483861	75279039	19	rs2074550	19387743
16	rs117782866	68023169	14	rs10138360	75293386	19	rs2074551	19388057
16	rs146418648	68023776	14	rs2058919	75310812	19	rs17750998	19388446
16	rs255052	68024995	14	rs2359239	75326771	19	rs17683766	19388888
16	rs73596365	68026126	14	rs722599	75327443	19	rs11555053	19390185
16	rs10527563	68030270	14	rs28447028	75340133	19	rs2301784	19390749
16	rs7204974	68033539	14	rs7146160	75346451	19	rs6511026	19391402
16	rs8059305	68036666	14	rs2268613	75408500	19	rs11671506	19391838
16	rs79577483	68036939	14	rs11850328	75426250	19	rs6511027	19391851
16	rs11075664	68038763	14	rs175502	75533679	19	rs2269873	19392401
16	rs7204192	68039309	14	rs175495	75542182	19	rs2023882	19405405
16	rs16957696	68039850	14	rs96358	75572265	19	rs2023883	19405480
16	rs76646352	68044797	14	rs7145159	75583268	19	rs74182633	19406707
16	rs9674047	68045046	14	rs12883497	75587014	19	rs12979148	19406869
16	rs56377913	68047851	14	rs1570712	103783847	19	rs10401969	19407718
16	rs78962063	68052481	14	rs17677788	103792698	19	rs2301785	19408164
16	rs2074575	68055378	14	rs8009817	103815090	19	rs4808939	19410480
16	rs13816	68055495	14	rs7155749	103825318	19	rs2315022	19413381
16	rs237831	68057096	14	rs3759579	103851272	19	rs2315023	19413393
16	rs149214641	68058643	14	rs8014182	103859962	19	rs114573949	19414379
16	rs74652527	68060474	14	rs2010281	103862322	19	rs3752201	19415628
16	rs76836953	68061763	14	rs12879663	103874215	19	rs1010207	19416045
16	rs12448227	68068627	14	rs12896612	103877868	19	rs117109209	19417145
16	rs11075665	68070774	14	rs12882130	103878774	19	rs76944848	19417512
16	rs7193935	68071147	14	rs2065017	103879579	19	rs4808943	19420840
16	rs7190079	68073834	14	rs12885762	103882200	19	rs1859287	19422187
16	rs80346622	68074419	14	rs12892062	103883512	19	rs113189294	19423086
16	rs73598006	68074454	14	rs7158822	103887868	19	rs11668104	19426181
16	rs1985437	68074857	14	rs12885234	103889181	19	rs80146298	19426317
16	rs58388686	68075439	14	rs17679127	103890091	19	rs2315025	19426609
16	rs12445533	68082527	14	rs12897976	103893010	19	rs8108647	19427623

16	rs12598256	68084021	14	rs8015712	103895840	19	rs3934667	19431423
16	rs76418810	68084479	14	rs1951389	103898525	19	rs4539728	19431480
16	rs79323429	68084594	14	rs12894275	103901424	19	rs12460764	19431963
16	rs11555011	68088485	14	rs2065015	103904061	19	rs117924058	19432416
16	rs75909028	68089578	14	rs7155822	103922390	19	rs4808194	19435680
16	rs75899576	68090521	14	rs10459573	103925457	19	rs4239639	19435876
16	rs2418739	68092850	14	rs4906327	103929039	19	rs7247309	19439631
16	rs111227007	68093138	14	rs8012127	103942762	19	rs3764567	19440066
16	rs118156865	68095291	14	rs3783397	103947289	19	rs1009136	19440428
16	rs35432211	68095449	14	rs3783398	103949378	19	rs12985655	19442434
16	rs113993355	68099122	14	rs12896919	103956713	19	rs17751109	19443334
16	rs79375669	68099262	14	rs17617994	103959289	19	rs10640109	19443406
16	rs7201742	68099821	14	rs13987	103969909	19	rs8103197	19443466
16	rs2285912	68100461	14	rs4906331	103971374	19	rs74980519	19443968
16	rs113896227	68101480	14	rs12885786	103972503	19	rs117923614	19443986
16	rs75733050	68101941	14	rs4906332	103980134	19	rs11085259	19445856
16	rs75150864	68101998	14	rs2246490	103984294	19	rs12463074	19445869
16	rs2285910	68104422	14	rs2296487	103996205	19	rs769267	19446936
16	rs2301814	68105171	14	rs2236281	104000518	19	rs1989867	19447470
16	rs6499157	68105474	14	rs7161305	104002234	19	rs756999	19448200
16	rs116670584	68106547	14	rs9324063	104006693	19	rs757000	19448301
16	rs75421006	68106712	14	rs7140558	104018651	19	rs2965190	19451591
16	rs3785108	68106846	14	rs4906335	104021141	19	rs2301668	19452249
16	rs79422074	68106900	14	rs7148456	104028270	19	rs7250368	19452763
16	rs74680231	68108898	14	rs2296482	104029819	19	rs2965191	19453521
16	rs7188085	68113873	14	rs12889403	104034746	19	rs2301669	19453560
16	rs74882119	68113972	14	rs7140568	104041374	19	rs2965193	19454828
16	rs76501820	68114582	14	rs8017993	104047734	19	rs17751203	19455864
16	rs1124993	68114626	14	rs2403197	104053764	19	rs35824797	19456264
16	rs2107369	68116018	14	rs8021368	104074810	19	rs58489806	19456917
16	rs3785109	68118311	14	rs729438	104092789	19	rs34858588	19457235
16	rs3743735	68119555	14	rs709400	104149475	19	rs2905421	19457908
16	rs78537727	68120606	14	rs12432907	104168378	19	rs968525	19459215
16	rs75739761	68122914	14	rs8548	104199580	19	rs12982276	19459800
16	rs74519164	68123086	14	rs12886637	104222892	19	rs73001065	19460541
16	rs59708959	68123916	14	rs722637	104261723	19	rs2965194	19460702
16	rs237834	68125316	14	rs12883337	104264137	19	rs2905422	19460703
16	rs12446198	68125972	14	rs4900597	104266664	19	rs117949526	19462567
16	rs75735284	68126635	15	rs12901536	37425937	19	rs11085261	19462606
16	rs1074143	68127278	15	rs2203929	37455975	19	rs2965195	19463032
16	rs12599178	68128104	15	rs16954389	37494817	19	rs9304960	19465529
16	rs114329216	68129101	15	rs1356779	37560789	19	rs2301671	19466269
16	rs74604428	68129638	15	rs16964908	37638018	19	rs62135557	19466293
16	rs76595854	68130233	15	rs4586385	37845804	19	rs756997	19467085
16	rs78961715	68131407	16	rs955905	6065358	19	rs2285626	19467545
16	rs9932251	68131732	16	rs8048168	6087895	19	rs2285627	19467937
16	rs7192187	68132109	16	rs11641907	6102413	19	rs2285628	19467996
16	rs8058690	68136622	16	rs6500744	6113661	19	rs118029212	19468260

16	rs72790386	68136932	16	rs4360938	6116418	19	rs2965196	19468525
16	rs78479045	68137486	16	rs929887	6121557	19	rs13964	19468710
16	rs117784871	68138091	16	rs34151263	6123096	19	rs15622	19468734
16	rs151193115	68140780	16	rs9927211	6124714	19	rs10403731	19469296
16	rs12447640	68150527	16	rs12931744	6125544	19	rs1985109	19469907
16	rs12599880	68152035	16	rs4124065	6128400	19	rs2965198	19473030
16	rs7205935	68152157	16	rs2343519	6128520	19	rs56255430	19477877
16	rs73612690	68153200	16	rs929889	6131347	19	rs2905426	19478022
16	rs74024145	68153382	16	rs8052082	6131788	19	rs2905427	19478023
16	rs74333408	68153492	16	rs4786811	6132787	19	rs2965201	19478051
16	rs76419201	68153553	16	rs6500751	6132810	19	rs59148799	19484008
16	rs2418736	68154862	16	rs17139340	6133414	19	rs34277109	19487302
16	rs12445396	68155121	16	rs889701	6134669	19	rs12973258	19488718
16	rs118005841	68157562	16	rs4786815	6135185	19	rs2965180	19491973
16	rs3815173	68160289	16	rs9934817	6136219	19	rs10406278	19494725
16	rs73612697	68162016	16	rs12928388	6136356	19	rs62136975	19495652
16	rs117922739	68163168	16	rs889702	6139140	19	rs2905435	19495954
16	rs12597573	68167521	16	rs9936351	6141379	19	rs2965186	19497195
16	rs74851284	68172568	16	rs12927079	6142023	19	rs1858999	19497669
16	rs12597380	68172587	16	rs7199064	6145849	19	rs200738892	19498867
16	rs116867512	68180834	16	rs7197966	6146331	19	rs117131852	19499589
16	rs9922165	68181177	16	rs8062784	6146634	19	rs60321073	19499598
16	rs9938553	68183064	16	rs1424121	6146730	19	rs17288409	19504167
16	rs7193701	68185160	16	rs8047363	6147082	19	rs10415849	19505087
16	rs114739995	68188464	16	rs9806914	6147645	19	rs10424702	19508013
16	rs12446007	68189054	16	rs7189501	6175429	19	rs12462698	19510363
16	rs8044995	68189340	16	rs6500760	6180375	19	rs2965181	19512317
16	rs55725810	68189636	16	rs17139480	6211906	19	rs10408596	19512657
16	rs117806247	68189730	16	rs17139486	6214224	19	rs2965179	19512755
16	rs77884900	68190239	16	rs12103273	6252217	19	rs35156688	19514029
16	rs8060893	68191608	16	rs12924056	6267057	19	rs118013132	19515562
16	rs181018248	68192672	16	rs17817199	6283228	19	rs1000237	19518316
16	rs117620519	68194889	16	rs12444771	6300009	19	rs2965189	19519518
16	rs4359427	68202747	16	rs4786847	6310233	19	rs2916069	19520358
16	rs185107840	68204790	16	rs7206839	6318193	19	rs182252596	19520536
16	rs8046355	68205214	16	rs2160166	6328022	19	rs193098400	19522481
16	rs118030546	68206660	16	rs1420029	6377499	19	rs2060276	19522491
16	rs75796305	68208791	16	rs10521041	6408915	19	rs2965188	19522557
16	rs12448677	68209741	16	rs12447711	6411728	19	rs2916070	19524105
16	rs8044014	68209872	16	rs11643342	6449389	19	rs2916072	19525712
16	rs8048034	68210604	16	rs2109457	6450463	19	rs2965185	19525792
16	rs62057372	68211249	16	rs8056982	6452536	19	rs11436420	19526776
16	rs28548264	68211321	16	rs1860563	6478898	19	rs2916073	19528324
16	rs117240558	68212641	16	rs9939300	6487958	19	rs1469713	19528806
16	rs75297764	68212820	16	rs9939653	6488303	19	rs1469712	19528821
16	rs116883401	68213125	16	rs4786869	6496333	19	rs78150994	19530210
16	rs74775711	68213680	16	rs2024208	6497757	19	rs2916074	19530270
16	rs73612222	68216072	16	rs17140313	6519332	19	rs2916075	19530590

16	rs7190307	68218336	16	rs8060506	6523142	19	rs11668386	19531910
16	rs12445915	68218748	16	rs878012	6531429	19	rs2060275	19536208
16	rs9932414	68219969	16	rs809702	6548389	19	rs8182472	19539891
16	rs181812905	68220688	16	rs17442167	6549889	19	rs36113815	19540183
16	rs7199588	68224582	16	rs7404493	6552165	19	rs113702251	19541147
16	rs12598	68225515	16	rs12449216	6553772	19	rs11313442	19541787
16	rs1125332	68227780	16	rs12449258	7433021	19	rs34498230	19544403
16	rs74692270	68227973	16	rs11640126	7473895	19	rs4808199	19545099
16	rs1125331	68228119	16	rs4787016	7476725	19	rs2965184	19545428
16	rs55757502	68228427	16	rs1034983	7492515	19	rs113564583	19545642
16	rs114645460	68238511	16	rs2109437	7493024	19	rs2965183	19545696
16	rs113502835	68238965	16	rs11077189	7535158	19	rs2916076	19545990
16	rs6499162	68239460	16	rs12929155	7535379	19	rs4808951	19550306
16	rs6499163	68243486	16	rs8059548	7538252	19	rs76801207	19550696
16	rs1073632	68248607	16	rs3785267	7574054	19	rs80200208	19554725
16	rs9938020	68251784	16	rs17676059	7574482	19	rs4808200	19554803
16	rs117992789	68253882	16	rs1507023	7588435	19	rs76761722	19557965
16	rs117410267	68256622	16	rs17677211	7594064	19	rs8105642	19558468
16	rs9931987	68256639	16	rs4786182	7595701	19	rs8110250	19559989
16	rs12598630	68258852	16	rs17144090	7597891	19	rs8110171	19560063
16	rs8056649	68260083	16	rs6500986	7613177	19	rs12971734	19562253
16	rs11043	68262958	16	rs12444690	7666200	19	rs10414830	19562332
16	rs8057119	68268836	16	rs3785233	7667510	19	rs12972397	19562349
16	rs141865143	68269479	16	rs10492763	7669594	19	rs1560687	19562902
16	rs7184821	68270229	16	rs758491	7681778	19	rs4808957	19567248
16	rs117571960	68271261	16	rs17144422	7690419	19	rs12977524	19568244
16	rs7187202	68273895	16	rs17144509	7709203	19	rs4808203	19568659
16	rs3826164	68280317	16	rs740676	7712416	19	rs75582668	19569023
16	rs1971546	68280893	16	rs17135054	7713728	19	rs74322277	19570428
16	rs10500543	68281311	16	rs763650	7729221	19	rs58074958	19570555
16	rs12444188	68282077	16	rs917543	7743747	19	rs7250658	19571100
16	rs117958985	68283976	16	rs1014125	7781985	19	rs4808958	19571752
16	rs1975802	68285847	16	rs1024647	7792248	19	rs4808959	19572108
16	rs17688065	68286002	16	rs1024646	7792414	19	rs28720066	19572220
16	rs116886316	68286291	16	rs2967569	7812952	19	rs4808960	19574277
16	rs2290700	68288983	16	rs2907359	7817528	19	rs200232664	19575945
16	rs200107219	68289059	16	rs2967557	7818955	19	rs2163804	19575965
16	rs8062085	68289313	16	rs3111558	7831214	19	rs4808961	19577215
16	rs12446418	68290200	16	rs3112738	7834885	19	rs2099334	19578450
16	rs117793858	68291767	16	rs4238884	7840181	19	rs754256	19578591
16	rs7672	68294800	16	rs4456518	7860392	19	rs73002956	19578743
16	rs2863981	68295598	16	rs12931037	7861208	19	rs754255	19578890
16	rs9888986	68297228	16	rs4620976	7873825	19	rs10405625	19579046
16	rs9888796	68297589	16	rs11077231	7874445	19	rs4808962	19579557
16	rs12447119	68297928	16	rs13337565	7876317	19	rs6511036	19582651
16	rs12102971	68298024	16	rs4290490	7885694	19	rs73002960	19582992
16	rs12162068	68298193	16	rs4608356	7915055	19	rs17684098	19584969
16	rs116580887	68299502	16	rs11075148	12829006	19	rs747050	19584987

16	rs117417273	68299697	16	rs11075151	12834880	19	rs56280531	19585788
16	rs12596500	68299771	16	rs7190046	12839092	19	rs1465695	19588546
16	rs77517043	68301410	16	rs12446662	12859891	19	rs148037950	19590022
16	rs72790400	68301727	16	rs4780476	12862007	19	rs75260583	19590232
16	rs9933029	68303410	16	rs8045393	12864070	19	rs118182642	19597026
16	rs185291585	68304116	16	rs4536458	12871639	19	rs10419912	19597055
16	rs8056893	68304392	16	rs4513082	12878592	19	rs2315610	19597240
16	rs78131538	68305712	16	rs2060360	12888567	19	rs77157813	19598611
16	rs9935025	68307447	16	rs4238618	12946129	19	rs77653002	19599389
16	rs9937244	68307559	16	rs11644362	12994097	19	rs79879961	19599742
16	rs4783552	68310312	16	rs11075174	13020416	19	rs118013768	19602760
16	rs112086775	68310917	16	rs7499101	13032136	19	rs751858	19602821
17	rs2281727	2117945	16	rs961946	13033361	19	rs751857	19602873
17	rs143499	2132324	16	rs6498368	13050541	19	rs751856	19602945
17	rs7217226	2136065	16	rs7192086	13061611	19	rs4808964	19603692
17	rs10852932	2143460	16	rs251921	13202828	19	rs11671253	19604206
17	rs34166400	2146392	16	rs11075184	13279908	19	rs2288852	19605348
17	rs170040	2165714	16	rs12922227	13302210	19	rs1047361	19606634
17	rs170041	2170216	16	rs149228	13304474	19	rs73002973	19606848
17	rs216196	2202943	16	rs1549292	71983664	19	rs6511038	19607564
17	rs391300	2216258	16	rs7204708	71986946	20	rs2425656	43506247
17	rs12951376	17752809	16	rs16973520	71996291	20	rs6017444	43528521
17	rs7503334	17779458	16	rs8053891	71997789	20	rs12031	43561660
17	rs4299203	17878159	16	rs28714527	71997862	20	rs17322289	43599951
17	rs7219320	17880877	16	rs148461313	71998016	20	rs11477080	43670340
17	rs2955377	17932818	16	rs4788587	72001136	20	rs6031972	43704943
17	rs2955383	17941364	16	rs9930930	72001971	20	rs734784	43723627
17	rs16960835	17960011	16	rs35800852	72002105	20	rs962550	43725727
17	rs854764	18011750	16	rs11644740	72002308	20	rs6032006	43755053
18	rs4131791	52747871	16	rs35259348	72003952	20	rs17423416	43758144
18	rs4309482	52750469	16	rs8055242	72006882	20	rs17332620	43777506
18	rs11874716	52750688	16	rs7197967	72007232	20	rs13037651	43782230
18	rs12966547	52752017	16	rs9925415	72007399	20	rs6032029	43786064
18	rs8092679	53034140	16	rs34972708	72008225	20	rs979641	43786336
18	rs1660241	53035650	16	rs10597344	72008826	20	rs2868237	43794189
18	rs1788030	53045198	16	rs12931964	72009676	20	rs17424696	43810734
18	rs1788025	53048678	16	rs11405422	72009857	20	rs6032044	43819416
18	rs9646596	53049212	16	rs1559401	72011181	20	rs7344269	43828668
18	rs17594526	53058238	16	rs1559399	72011261	20	rs6017511	43833132
18	rs17594665	53063719	16	rs77282701	72011439	20	rs13037087	43834313
18	rs17089778	53065342	16	rs78529492	72011610	20	rs6017519	43854677
18	rs17594721	53065892	16	rs7185272	72013797	20	rs2868240	43874039
18	rs11152369	53066328	16	rs72803749	72014322	20	rs4522674	43893276
18	rs17509991	53067184	16	rs7191900	72014866	20	rs13039345	43895932
18	rs2958175	53074958	16	rs8063499	72016906	20	rs991048	43898227
18	rs10503001	53075696	16	rs117115894	72017084	20	rs6017530	43899731
18	rs2919450	53084545	16	rs72803753	72017437	20	rs6032087	43902305
18	rs4500831	53097544	16	rs7187692	72017495	20	rs17335251	43902946

18	rs12963463	53099093	16	rs12935551	72017562	20	rs6017532	43904095
18	rs4468713	53104019	16	rs10492825	72018417	20	rs6104092	43906730
18	rs10503002	53109202	16	rs10492824	72018771	20	rs6104093	43908297
18	rs12606995	53121576	16	rs10492823	72018860	20	rs7272524	43908694
18	rs732779	53123172	16	rs4788589	72019948	20	rs6032095	43913255
18	rs2924321	53125435	16	rs12708923	72020134	20	rs761367	43913317
18	rs1377243	53134652	16	rs4788590	72020294	20	rs2743330	43919704
18	rs8090422	53146896	16	rs4788591	72020323	20	rs2743334	43919745
18	rs9960767	53155002	16	rs9927968	72021767	20	rs2743246	43928698
18	rs2060889	53159541	16	rs12051425	72021962	20	rs2743283	43931096
18	rs17596974	53166557	16	rs150143219	72021980			
18	rs2958158	53172105	16	rs9938025	72022041			
18	rs13313612	53458908	16	rs889545	72022065			
18	rs6650723	53524269	16	rs12600121	72022454			
18	rs17201415	53780234	16	rs12600132	72022534			
19	rs8105094	19374061	16	rs12051517	72022866			
19	rs8105984	19374068	16	rs4788592	72022941			
19	rs35854118	19375372	16	rs9925967	72024846			
19	rs78538967	19375822	16	rs12927205	72025077			
19	rs2074298	19377716	16	rs7204798	72025429			
19	rs117877390	19378416	16	rs4788595	72025759			
19	rs998732	19378671	16	rs10852510	72026041			
19	rs58542926	19379549	16	rs11075912	72026554			
19	rs72999063	19380596	16	rs7191148	72027191			
19	rs2074299	19380646	16	rs192541717	72027321			
19	rs72999068	19380685	16	rs7195072	72027579			
19	rs12974359	19381439	16	rs17664900	72027611			
19	rs2074301	19381715	16	rs34156336	72028062			
19	rs2074303	19381755	16	rs79217285	72028079			
19	rs116904583	19382215	16	rs34150651	72028112			
19	rs55822665	19383298	16	rs72787018	72028200			
19	rs10419245	19383755	16	rs35697801	72028220			
19	rs78640921	19384914	16	rs12919469	72028612			
19	rs11669578	19385080	16	rs7191717	72029303			
19	rs735273	19385411	16	rs12920844	72029304			
19	rs8103496	19386015	16	rs62055599	72029797			
19	rs74182631	19386738	16	rs7197883	72030072			
19	rs2017964	19387149	16	rs72787021	72030688			
19	rs757346	19387185	16	rs72787023	72031105			
19	rs2010506	19387356	16	rs78534681	72031172			
19	rs2074550	19387743	16	rs1035559	72031860			
19	rs2074551	19388057	16	rs72787025	72031865			
19	rs17750998	19388446	16	rs16973585	72032231			
19	rs17683766	19388888	16	rs1035560	72032730			
19	rs11555053	19390185	16	rs17588429	72032751			
19	rs2301784	19390749	16	rs60493669	72033580			
19	rs6511026	19391402	16	rs72787027	72033801			
19	rs11671506	19391838	16	rs7185601	72035082			

19	rs6511027	19391851	16	rs7186207	72035359
19	rs2269873	19392401	16	rs74343175	72035477
19	rs2023882	19405405	16	rs8051239	72036257
19	rs2023883	19405480	16	rs7195958	72036577
19	rs74182633	19406707	16	rs4788454	72037206
19	rs12979148	19406869	16	rs929866	72038363
19	rs10401969	19407718	16	rs2012817	72038659
19	rs2301785	19408164	16	rs56051977	72039207
19	rs4808939	19410480	16	rs1820249	72039446
19	rs2315022	19413381	16	rs1820248	72039605
19	rs2315023	19413393	16	rs9929256	72040032
19	rs114573949	19414379	16	rs34596355	72040791
19	rs3752201	19415628	16	rs3213419	72042428
19	rs1010207	19416045	16	rs34270657	72042635
19	rs117109209	19417145	16	rs3213422	72042682
19	rs76944848	19417512	16	rs4788597	72043039
19	rs4808943	19420840	16	rs4788598	72044238
19	rs1859287	19422187	16	rs2878404	72045186
19	rs113189294	19423086	16	rs8057016	72046211
19	rs11668104	19426181	16	rs1465457	72046268
19	rs80146298	19426317	16	rs8058214	72046305
19	rs2315025	19426609	16	rs8061140	72046461
19	rs8108647	19427623	16	rs752434	72047170
19	rs3934667	19431423	16	rs4788456	72047286
19	rs4539728	19431480	16	rs4788600	72048080
19	rs12460764	19431963	16	rs3764310	72048332
19	rs117924058	19432416	16	rs8062895	72048632
19	rs4808194	19435680	16	rs7184117	72049212
19	rs4239639	19435876	16	rs3812988	72049621
19	rs7247309	19439631	16	rs11864453	72050480
19	rs3764567	19440066	16	rs75329497	72052299
19	rs1009136	19440428	16	rs11648003	72052348
19	rs12985655	19442434	16	rs10048111	72053141
19	rs17751109	19443334	16	rs1862752	72054984
19	rs10640109	19443406	16	rs2081223	72055532
19	rs8103197	19443466	16	rs12446480	72056757
19	rs74980519	19443968	16	rs2288002	72057282
19	rs117923614	19443986	16	rs61733129	72057421
19	rs11085259	19445856	16	rs2288000	72058881
19	rs12463074	19445869	16	rs113383183	72059556
19	rs769267	19446936	16	rs55684229	72062019
19	rs1989867	19447470	16	rs190425537	72062357
19	rs756999	19448200	16	rs2550868	72066072
19	rs757000	19448301	16	rs72787038	72066102
19	rs2965190	19451591	16	rs74386648	72066442
19	rs2301668	19452249	16	rs12925078	72067263
19	rs7250368	19452763	16	rs2550043	72067491
19	rs2965191	19453521	16	rs117365732	72069740

19	rs2301669	19453560	16	rs79635500	72069966
19	rs2965193	19454828	16	rs2550870	72070129
19	rs17751203	19455864	16	rs182238023	72070574
19	rs35824797	19456264	16	rs188036452	72070575
19	rs58489806	19456917	16	rs251033	72073122
19	rs34858588	19457235	16	rs9932951	72075341
19	rs2905421	19457908	16	rs76809174	72076668
19	rs968525	19459215	16	rs12930702	72077000
19	rs12982276	19459800	16	rs763665	72078043
19	rs73001065	19460541	16	rs76057084	72078485
19	rs2965194	19460702	16	rs1424241	72078907
19	rs2905422	19460703	16	rs35283911	72078990
19	rs117949526	19462567	16	rs7197453	72079127
19	rs11085261	19462606	16	rs72787046	72079342
19	rs2965195	19463032	16	rs8182213	72080005
19	rs9304960	19465529	16	rs113530185	72080802
19	rs2301671	19466269	16	rs9924964	72085561
19	rs62135557	19466293	16	rs5467	72088280
19	rs756997	19467085	16	rs5468	72088331
19	rs2285626	19467545	16	rs8062041	72088964
19	rs2285627	19467937	16	rs12917999	72089451
19	rs2285628	19467996	16	rs2070937	72089740
19	rs118029212	19468260	16	rs5475	72094348
19	rs2965196	19468525	16	rs34682685	72096227
19	rs13964	19468710	16	rs7189115	72098952
19	rs15622	19468734	16	rs7188962	72098962
19	rs10403731	19469296	16	rs7190994	72099241
19	rs1985109	19469907	16	rs34042070	72101525
19	rs2965198	19473030	16	rs79469260	72103769
19	rs56255430	19477877	16	rs79679679	72104835
19	rs2905426	19478022	16	rs72787058	72105084
19	rs2905427	19478023	16	rs152838	72105702
19	rs2965201	19478051	16	rs11642506	72107865
19	rs59148799	19484008	16	rs2000999	72108093
19	rs34277109	19487302	16	rs11646364	72108687
19	rs12973258	19488718	16	rs55741258	72112480
19	rs2965180	19491973	16	rs74029810	72113345
19	rs10406278	19494725	16	rs2550035	72113416
19	rs62136975	19495652	16	rs117596524	72113914
19	rs2905435	19495954	16	rs217181	72114002
19	rs2965186	19497195	16	rs74029814	72114715
19	rs1858999	19497669	16	rs2854956	72115238
19	rs200738892	19498867	16	rs8059437	72116467
19	rs74604097	19499549	16	rs56901884	72117823
19	rs117131852	19499589	16	rs117783250	72119422
19	rs60321073	19499598	16	rs61282680	72119628
19	rs17288409	19504167	16	rs78310258	72120958
19	rs10415849	19505087	16	rs62055637	72121265

19	rs10424702	19508013	16	rs78873516	72121487
19	rs12462698	19510363	16	rs10492813	72122463
19	rs2965181	19512317	16	rs12708925	72122706
19	rs10408596	19512657	16	rs74644576	72122761
19	rs2965179	19512755	16	rs116949987	72123423
19	rs35156688	19514029	16	rs152828	72123886
19	rs118013132	19515562	16	rs7191127	72123992
19	rs1000237	19518316	16	rs7190995	72124157
19	rs2965189	19519518	16	rs7192730	72124219
19	rs2916069	19520358	16	rs2303285	72124495
19	rs182252596	19520536	16	rs146137507	72124787
19	rs193098400	19522481	16	rs3764311	72125617
19	rs2060276	19522491	16	rs3764312	72125733
19	rs2965188	19522557	16	rs10492814	72126514
19	rs2916070	19524105	16	rs3764314	72127078
19	rs2916072	19525712	16	rs7194397	72128299
19	rs2965185	19525792	16	rs12924413	72129745
19	rs11436420	19526776	16	rs35794819	72130103
19	rs2916073	19528324	16	rs11554764	72130125
19	rs1469713	19528806	16	rs1050361	72130203
19	rs1469712	19528821	16	rs9926927	72130556
19	rs78150994	19530210	16	rs1050362	72130815
19	rs2916074	19530270	16	rs17590101	72132048
19	rs2916075	19530590	16	rs6499559	72132064
19	rs11668386	19531910	16	rs11862813	72132356
19	rs2060275	19536208	16	rs2072142	72132713
19	rs8182472	19539891	16	rs17287146	72133789
19	rs36113815	19540183	16	rs2269918	72134831
19	rs113702251	19541147	16	rs1050363	72135014
19	rs11313442	19541787	16	rs8043606	72135319
19	rs34498230	19544403	16	rs9932062	72136055
19	rs4808199	19545099	16	rs61060922	72136154
19	rs2965184	19545428	16	rs2074627	72136769
19	rs113564583	19545642	16	rs12708928	72137227
19	rs2965183	19545696	16	rs2240243	72137561
19	rs2916076	19545990	16	rs12325142	72138112
19	rs4808951	19550306	16	rs2074626	72139184
19	rs76801207	19550696	16	rs150617	72139396
19	rs80200208	19554725	16	rs55772151	72139667
19	rs4808200	19554803	16	rs118168140	72139689
19	rs76761722	19557965	16	rs58152051	72140338
19	rs8105642	19558468	16	rs36033103	72142038
19	rs8110250	19559989	16	rs8051882	72142072
19	rs8110171	19560063	16	rs9925763	72142595
19	rs12971734	19562253	16	rs9302635	72144174
19	rs10414830	19562332	16	rs2241412	72144284
19	rs12972397	19562349	16	rs7940	72146441
19	rs1560687	19562902	16	rs187003456	72147186

19	rs4808957	19567248	16	rs12448315	72147242
19	rs12977524	19568244	16	rs17667053	72147431
19	rs4808203	19568659	16	rs7184169	72152933
19	rs75582668	19569023	16	rs4788460	72154509
19	rs74322277	19570428	16	rs17604185	72194763
19	rs58074958	19570555	16	rs12927014	72207843
19	rs7250658	19571100	16	rs17604676	72221984
19	rs4808958	19571752	16	rs16970670	72225187
19	rs4808959	19572108	16	rs7203307	72235076
19	rs28720066	19572220	16	rs12447857	72237535
19	rs4808960	19574277	16	rs8060878	72243624
19	rs200232664	19575945	16	rs7187517	72250217
19	rs2163804	19575965	16	rs811054	72251132
19	rs4808961	19577215	16	rs9938506	72252752
19	rs2099334	19578450	16	rs12934528	72256903
19	rs754256	19578591	16	rs811049	72260046
19	rs73002956	19578743	16	rs4238965	72283147
19	rs754255	19578890	16	rs11646048	72291732
19	rs10405625	19579046	16	rs16970751	72297706
19	rs4808962	19579557	16	rs7191673	72308631
19	rs6511036	19582651	16	rs12921986	72312727
19	rs73002960	19582992	16	rs9930344	72381557
19	rs17684098	19584969	16	rs7193859	72400879
19	rs747050	19584987	16	rs2336438	72409698
19	rs56280531	19585788	16	rs806742	72432949
19	rs1465695	19588546	16	rs806739	72437741
19	rs148037950	19590022	17	rs7220474	27341434
19	rs75260583	19590232	17	rs1844754	27341776
19	rs118182642	19597026	17	rs997996	27351320
19	rs10419912	19597055	17	rs7212295	27431425
19	rs2315610	19597240	17	rs2279959	27437208
19	rs77157813	19598611	17	rs8076604	27438469
19	rs77653002	19599389	17	rs882729	27449976
19	rs79879961	19599742	17	rs6505114	27499140
19	rs118013768	19602760	17	rs11080090	27502029
19	rs751858	19602821	17	rs9906043	27504921
19	rs751857	19602873	17	rs9906526	27555828
19	rs751856	19602945	17	rs9303624	27562990
19	rs4808964	19603692	17	rs7224755	27567793
19	rs11671253	19604206	17	rs2041156	27568780
19	rs2288852	19605348	17	rs16964949	27582578
19	rs1047361	19606634	17	rs8614	27588806
19	rs73002973	19606848	17	rs2429244	27597286
19	rs6511038	19607564	17	rs16964963	27603316
19	rs10402451	19608537	17	rs797973	27647630
19	rs3794990	19610030	17	rs11867749	27658233
19	rs3794991	19610596	17	rs11653144	27675226
19	rs3794993	19611550	17	rs7222766	27679725

19	rs7259773	19611774	17	rs1400790	27696371
19	rs892021	19613480	17	rs2138852	27703349
19	rs57009615	19613622	17	rs12938678	27710427
19	rs79370636	19614289	17	rs8076739	27714587
19	rs78010159	19615905	17	rs9900684	27732942
19	rs3752151	19616026	17	rs1568373	27760143
19	rs1063966	19616742	17	rs9900280	27769598
19	rs1054284	19616953	17	rs17225878	27779438
19	rs34667451	19617017	17	rs771428	27793559
19	rs2033481	19617134	17	rs12601608	27800863
19	rs41313155	19617655	17	rs508369	27801070
19	rs1054308	19617656	17	rs6505138	27809104
19	rs10282	19619317	18	rs2861863	36633908
19	rs6909	19619542	18	rs4239424	36763264
19	rs17655544	19620300	18	rs12456709	36779973
19	rs180818546	19620460	18	rs9966656	36876506
19	rs34183201	19621389	18	rs2163544	36885075
19	rs11085264	19621780	18	rs10502722	36886378
19	rs7250893	19625547	18	rs16971807	36898689
19	rs80007081	19626734	18	rs9304205	36899968
19	rs17684164	19626769	18	rs12964741	36905627
19	rs77254326	19626781	18	rs1431702	36910969
19	rs16996127	19627310	18	rs1367836	36934371
19	rs7252888	19628037	18	rs2849762	36966260
19	rs78883953	19630002	18	rs1030593	50374155
19	rs7250233	19630948	18	rs7506794	50375592
19	rs4808965	19631655	18	rs4384683	50379032
19	rs11669010	19632378	18	rs7234775	50426610
19	rs113611839	19634000	18	rs1943121	50445517
19	rs78176666	19636702	18	rs16956268	50695211
19	rs111406321	19637437	18	rs10502962	50711414
19	rs75453386	19637781	18	rs8084351	50726559
19	rs73002997	19637793	18	rs8098405	50752373
19	rs75525243	19638218	18	rs1451949	52290083
19	rs45631651	19638743	18	rs1037391	52367823
19	rs74950305	19639448	18	rs2198229	52369403
19	rs4808967	19640524	18	rs2864762	52373227
19	rs55927782	19642032	18	rs7238608	52375542
19	rs10422819	19643028	18	rs1833302	52421470
19	rs113527843	19643343	18	rs8092503	52479487
19	rs8100927	19643636	18	rs8090635	52665858
19	rs8101219	19643715	18	rs12954483	52734527
19	rs34539063	19643907	18	rs4131791	52747871
19	rs4808206	19645645	18	rs4309482	52750469
19	rs2304098	19646272	18	rs11874716	52750688
19	rs1054930	19648346	18	rs12966547	52752017
19	rs11669730	19648713	18	rs9965955	52764278
19	rs11670775	19649636	18	rs11665032	52886611

19	rs11670882	19649748	18	rs7237259	52902885
19	rs4808208	19650096	18	rs1261115	52940528
19	rs182557669	19650389	18	rs1261117	52949657
19	rs76790018	19652157	18	rs8092679	53034140
19	rs8102502	19652746	18	rs1660241	53035650
19	rs77616520	19652776	18	rs1788030	53045198
19	rs77427798	19652982	18	rs1788025	53048678
19	rs188263117	19653525	18	rs9646596	53049212
19	rs7252453	19654117	18	rs17594526	53058238
19	rs45556231	19654189	18	rs17594665	53063719
19	rs61744761	19656615	18	rs17089778	53065342
19	rs79765293	19657041	18	rs17594721	53065892
19	rs1036215	19657198	18	rs11152369	53066328
19	rs10402308	19657500	18	rs17509991	53067184
19	rs73924805	19657590	18	rs2958175	53074958
19	rs7245983	19657632	18	rs10503001	53075696
19	rs16964240	30982538	18	rs2919450	53084545
19	rs12980596	31015438	18	rs4500831	53097544
20	rs2254603	37381627	18	rs12963463	53099093
20	rs6128907	37387862	18	rs4468713	53104019
20	rs1006945	37458009	18	rs10503002	53109202
22	rs5750854	39990775	18	rs12606995	53121576
22	rs738168	40011697	18	rs732779	53123172
22	rs4821981	41415644	18	rs2924321	53125435
22	rs926914	41418154	18	rs1377243	53134652
22	rs8139287	41444686	18	rs8090422	53146896
22	rs5758209	41461865	18	rs9960767	53155002
22	rs139484	41627543	18	rs2060889	53159541
22	rs5751069	41627775	18	rs17596974	53166557
22	rs1953	41641984	18	rs2958158	53172105
22	rs8139515	41673592	18	rs627685	53186092
22	rs10483213	42339525	18	rs10401120	53192498
22	rs8140869	42344297	18	rs17512836	53194961
22	rs710194	42392560	18	rs17597926	53205938
22	rs133309	42398606	22	rs7287939	41396353
22	rs133315	42402005	22	rs4821981	41415644
22	rs133341	42418185	22	rs926914	41418154
22	rs16947	42523943	22	rs8139287	41444686
22	rs5751232	42564304	22	rs5758209	41461865
22	rs134871	42652716	22	rs139484	41627543
22	rs17478318	42658821	22	rs5751069	41627775
22	rs17002948	42688724	22	rs1953	41641984
23	rs1882411	5972382	22	rs8139515	41673592
23	rs11798753	6024351	22	rs7290005	41742312
			22	rs2073167	41791536
			22	rs727563	41867377

Where: all SNPs have a MAF >=1% in an European, high CVD-risk cohort; Positions are given for build 37.

STable4: Eye colour-associated loci

CHR	POS	STRONGEST SNP	RISK ALLELE	Locus start	Locus end
1	1625671	rs117489126	T	1225671	2025671
1	30831778	rs35772196	G	30431778	31231778
1	109511287	rs569438828	A	109111287	109911287
1	121629918	rs201338907	G	121229918	122029918
1	149341656	rs112349824	TA	148941656	149741656
1	157828210	rs2765493	A	157428210	158228210
1	162294228	rs6690162	G	161894228	162694228
1	189811365	rs66527028	GTTT	189411365	190211365
1	203732372	rs146210682	G	203332372	204132372
1	205143783	rs3795556	?	204743783	205543783
1	207739063	rs2761430	A	207339063	208139063
1	216460132	rs6679773	T	216060132	216860132
1	221444418	rs140309399	T	221044418	221844418
1	232724717	rs533931579	T	232324717	233124717
1	234665431	rs35591409	CT	234265431	235065431
2	5576303	rs12478516	A	5176303	5976303
2	41451646	rs140756008	A	41051646	41851646
2	77699676	rs137957525	TTG	77299676	78099676
2	91579868	rs2599144	T	91179868	91979868
2	113827055	rs72948686	G	113427055	114227055
2	149274161	rs79553038	G	148874161	149674161
2	169432257	rs140193700	A	169032257	169832257
2	173685544	rs141283719	A	173285544	174085544
2	184515045	rs34370146	C	184115045	184915045
2	184583504	rs359899	C	184183504	184983504
2	185205313	rs10171577	C	184805313	185605313
2	185323105	rs6704925	G	184923105	185723105
2	195124829	rs79386120	T	194724829	195524829
2	226266139	rs566561215	C	225866139	226666139
2	240081084	rs13432025	A	239681084	240481084
3	1120489	rs116546495	G	720489	1520489
3	19958037	rs147723444	G	19558037	20358037
3	36294583	rs56795768	C	35894583	36694583
3	60374918	rs146752463	A	59974918	60774918
3	105208874	rs556316811	CA	104808874	105608874
3	111151943	rs75754256	A	110751943	111551943
3	140360027	rs6789157	T	139960027	140760027
3	159520652	rs139426027	C	159120652	159920652
3	167381185	rs13064599	A	166981185	167781185
3	167762873	rs9819158	?	167362873	168162873
3	189361929	rs699322	G	188961929	189761929
4	13947352	rs74654257	G	13547352	14347352
4	37292968	rs7691308	T	36892968	37692968
4	55706027	rs35104894	TA	55306027	56106027
4	101246775	rs573407928	G	100846775	101646775

4	121396276	rs7665234	G	120996276	121796276
4	173360857	rs533833761	G	172960857	173760857
4	183859179	rs892837	C	183459179	184259179
4	186011847	rs1542371	A	185611847	186411847
5	16422835	rs2596389	A	16022835	16822835
5	22567147	rs431616	A	22167147	22967147
5	33951588	rs16891982	?	33551588	34351588
5	33958854	rs28777	?	33558854	34358854
5	61597684	rs142980341	C	61197684	61997684
5	157085760	rs67467610	T	156685760	157485760
6	396321	rs12203592	?	-3679	796321
6	2187322	rs75749315	T	1787322	2587322
6	4371835	rs73717307	T	3971835	4771835
6	23918570	rs114137292	G	23518570	24318570
6	27038151	rs563894686	TTATTATATATAACATATTATATATAATACA	26638151	27438151
6	66387014	rs9354421	C	65987014	66787014
6	100456684	rs140839696	C	100056684	100856684
6	106478001	rs74371164	T	106078001	106878001
6	156874846	rs2063714	G	156474846	157274846
7	15027099	rs12113831	T	14627099	15427099
7	18991407	rs4721735	A	18591407	19391407
7	41183370	rs113052989	T	40783370	41583370
7	44381155	rs11765303	T	43981155	44781155
7	44510263	rs13236147	A	44110263	44910263
7	47522928	rs370083525	CAAAAA	47122928	47922928
7	55603936	rs539325600	A	55203936	56003936
7	57254662	rs13238329	C	56854662	57654662
7	67432818	rs186582231	T	67032818	67832818
7	67634233	rs6946516	T	67234233	68034233
7	68005118	rs570477080	C	67605118	68405118
7	93968999	rs34911443	T	93568999	94368999
7	94117145	rs375313288	T	93717145	94517145
7	114774843	rs78186662	C	114374843	115174843
7	114900774	rs144133613	T	114500774	115300774
7	144780854	rs375080348	A	144380854	145180854
7	151421400	rs57687654	A	151021400	151821400
8	40805553	rs4434606	T	40405553	41205553
8	84617435	rs570913543	T	84217435	85017435
8	107510196	rs7003565	T	107110196	107910196
9	12682663	rs10809826	?	12282663	13082663
9	18868649	rs1433830	C	18468649	19268649
9	25502384	rs62533293	G	25102384	25902384
9	39083501	rs2210426	A	38683501	39483501
9	84136877	rs10512144	C	83736877	84536877
9	89843099	chr9:89843099	T	89443099	90243099
9	91577228	rs9409609	A	91177228	91977228
9	92578859	rs534245958	T	92178859	92978859
9	96130316	rs74879286	G	95730316	96530316

9	98827361	rs10987872	C	98427361	99227361
9	109408422	rs35463120	TAAAC	109008422	109808422
9	115315096	rs376886874	AT	114915096	115715096
9	125411976	rs146271698	T	125011976	125811976
10	2488391	rs2458713	A	2088391	2888391
10	29340037	rs1774936	A	28940037	29740037
10	29342623	rs789923	A	28942623	29742623
10	47618087	chr10:47618087	G	47218087	48018087
10	61867180	rs2588926	C	61467180	62267180
10	117804632	rs11198112	?	117404632	118204632
10	122558603	rs3758430	A	122158603	122958603
10	124880660	rs28634972	G	124480660	125280660
10	129677743	rs12256016	G	129277743	130077743
11	40678692	rs191959849	A	40278692	41078692
11	88778356	rs7118677	?	88378356	89178356
11	89284793	rs1126809	?	88884793	89684793
11	113529872	rs192035988	A	113129872	113929872
12	4935882	rs554663181	C	4535882	5335882
12	15177959	rs114383698	T	14777959	15577959
12	113376164	rs574157881	A	112976164	113776164
12	113979299	rs11066860	A	113579299	114379299
13	19679321	chr13:19679321	T	19279321	20079321
13	72438916	rs117076724	A	72038916	72838916
13	77243655	rs4884060	C	76843655	77643655
13	91332148	rs55783125	A	90932148	91732148
14	22809964	rs11157836	A	22409964	23209964
14	47517162	rs533475206	AT	47117162	47917162
15	27968704	rs4778219	?	27568704	28368704
15	27985172	rs1800407	?	27585172	28385172
15	27990627	rs1800404	?	27590627	28390627
15	28120472	rs12913832	?	27720472	28520472
15	28135372	rs4778249	?	27735372	28535372
15	28147115	rs12898729	A	27747115	28547115
15	28165345	rs12916300	T	27765345	28565345
15	28250810	rs12912427	G	27850810	28650810
15	48134287	rs1426654	G	47734287	48534287
15	48832207	rs12899937	A	48432207	49232207
15	80552517	rs73494721	A	80152517	80952517
15	88977935	rs56405824	A	88577935	89377935
15	91134327	rs1003759	C	90734327	91534327
16	13261212	rs142469774	G	12861212	13661212
16	18767203	rs2606433	T	18367203	19167203
16	19053308	rs11409750	CT	18653308	19453308
16	32044223	rs142065243	C	31644223	32444223
16	55869460	rs567091563	T	55469460	56269460
16	84057293	rs3785011	A	83657293	84457293
16	88227277	rs9930276	A	87827277	88627277
17	1194826	chr17:1194826	A	794826	1594826

17	2018703	rs117239777	T	1618703	2418703
17	12321905	rs2529687	C	11921905	12721905
17	12747980	rs144953267	T	12347980	13147980
17	13192259	rs12325725	A	12792259	13592259
17	39491979	chr17:39491979	A	39091979	39891979
17	65957237	rs118011793	A	65557237	66357237
18	7763365	rs594771	A	7363365	8163365
18	41848645	rs78036593	T	41448645	42248645
18	42651868	rs592278	A	42251868	43051868
18	46012347	rs3886769	A	45612347	46412347
18	47241746	rs141768436	G	46841746	47641746
18	48797495	rs62103272	T	48397495	49197495
19	14609046	rs145155710	T	14209046	15009046
19	57357511	rs34411126	C	56957511	57757511
20	23090394	rs6048544	A	22690394	23490394
20	45110478	rs17422688	?	44710478	45510478
21	16221066	rs553005340	G	15821066	16621066
21	28334243	rs138716730	GT	27934243	28734243
22	27003613	rs2516084	C	26603613	27403613
22	37028950	rs5756492	?	36628950	37428950
22	44342526	rs6519897	G	43942526	44742526
22	46425412	rs5767203	T	46025412	46825412

Where: Positions are given for build 37.

Supplemental Figure 1

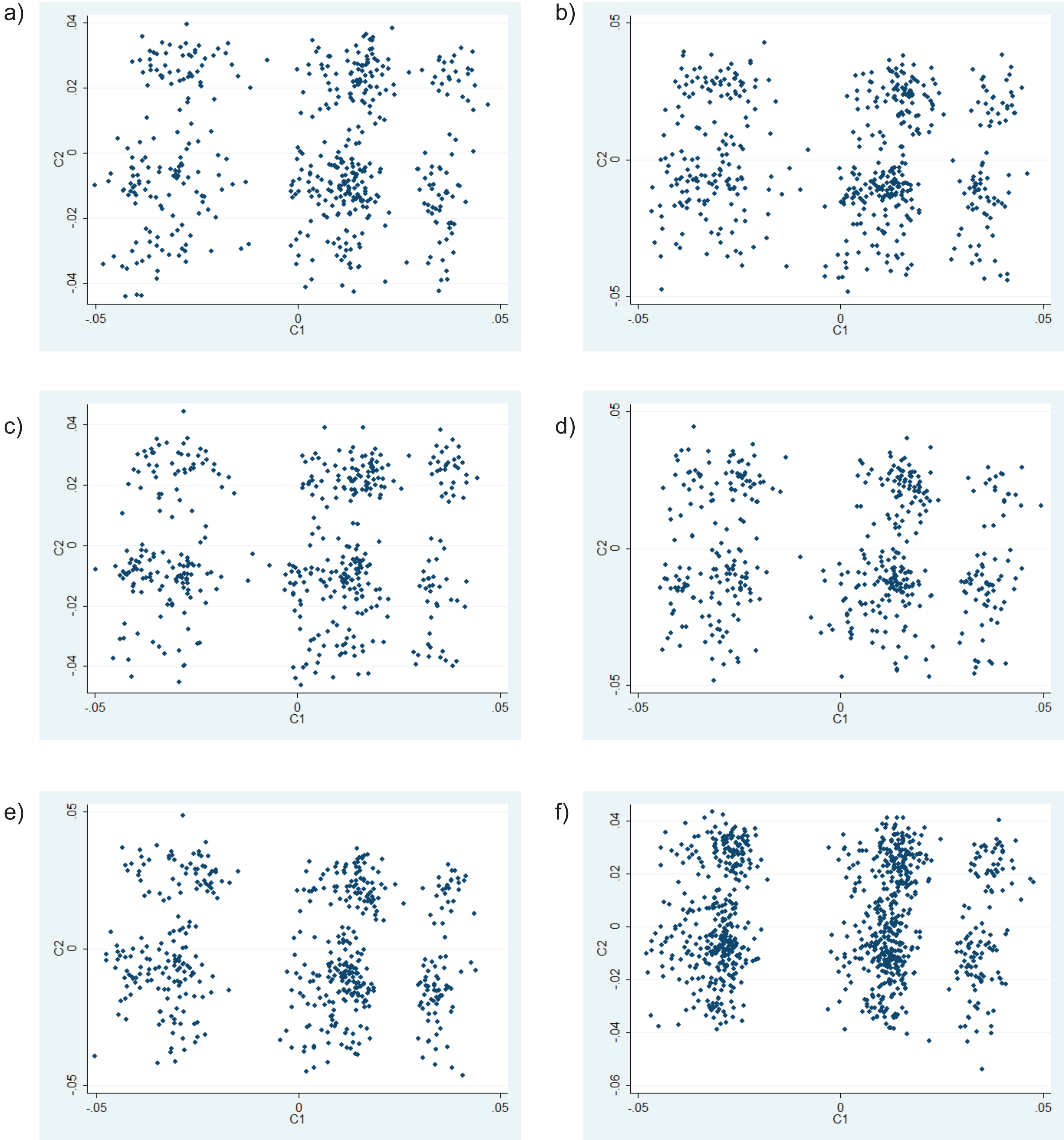

Supplementary Figure 2

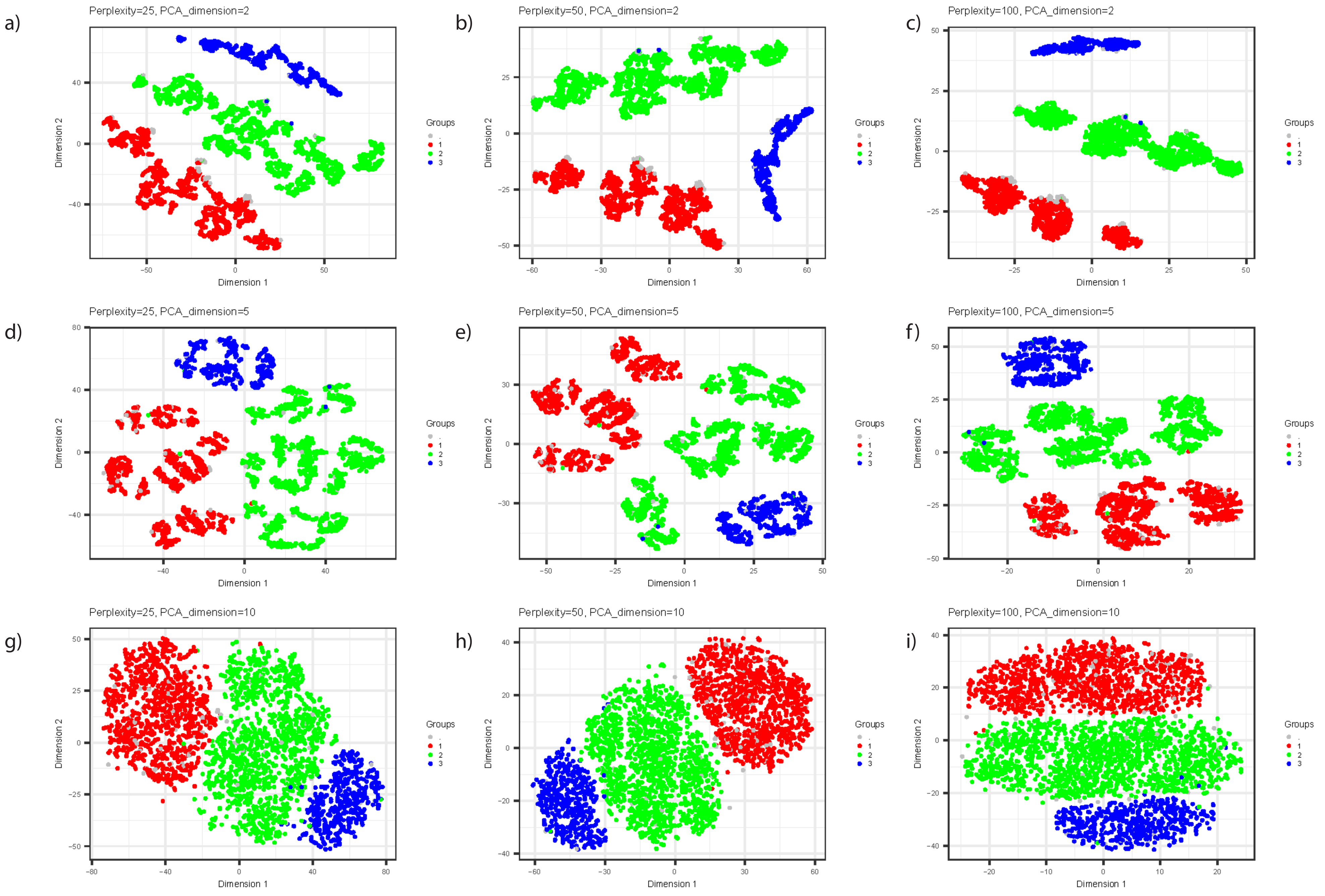

Supplementary Figure 3

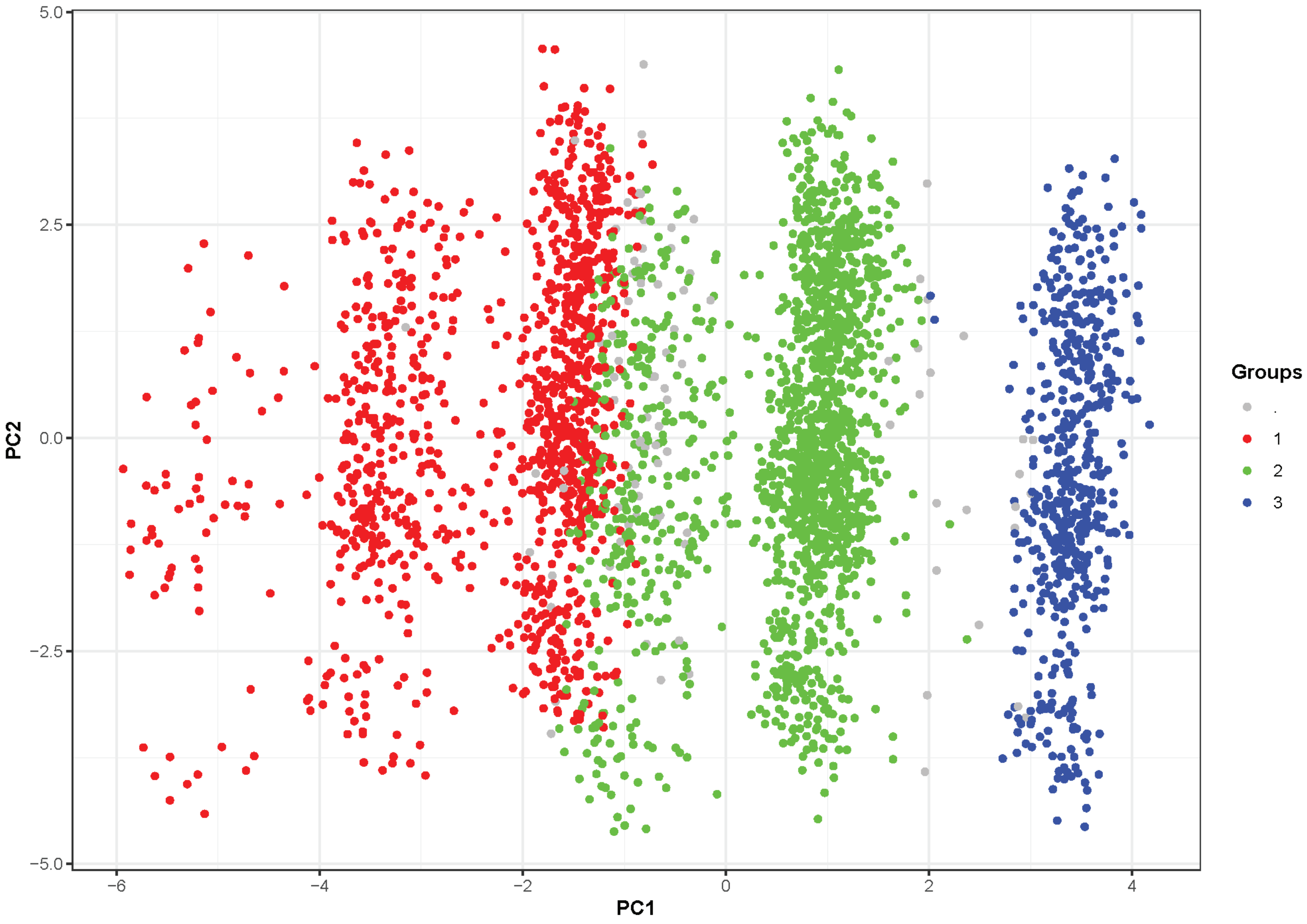

Supplementary Figure 4

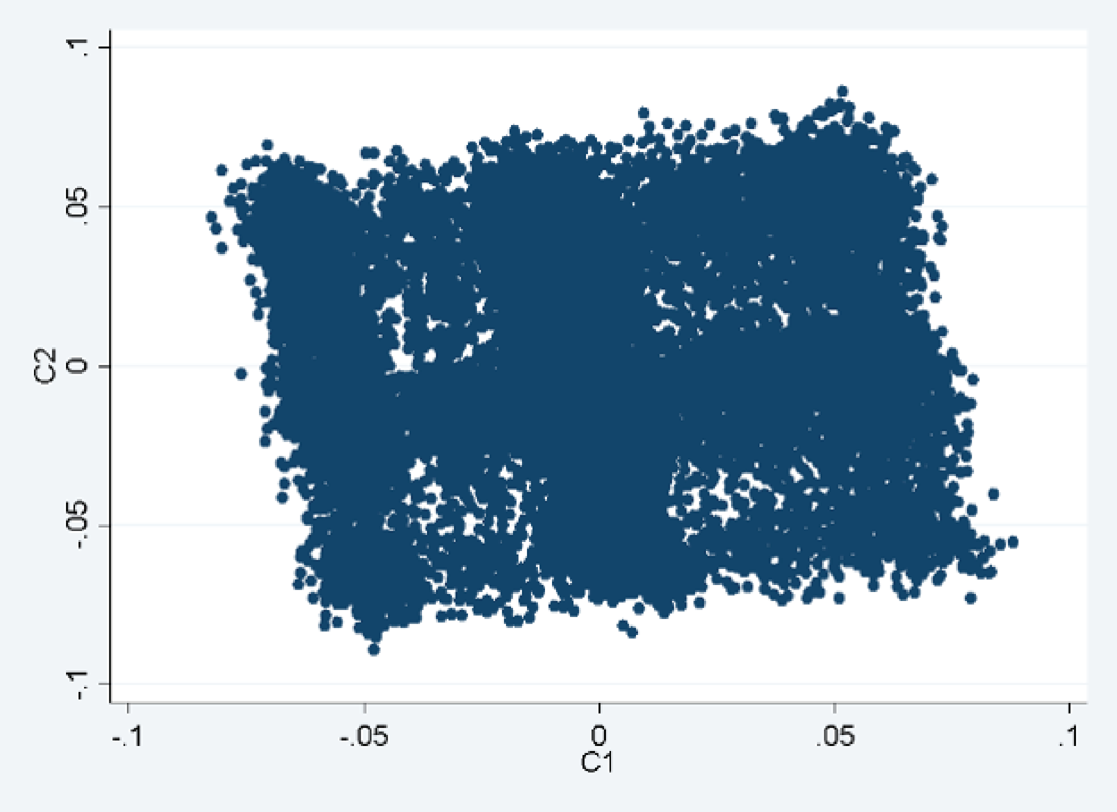

Supplementary Figure 5

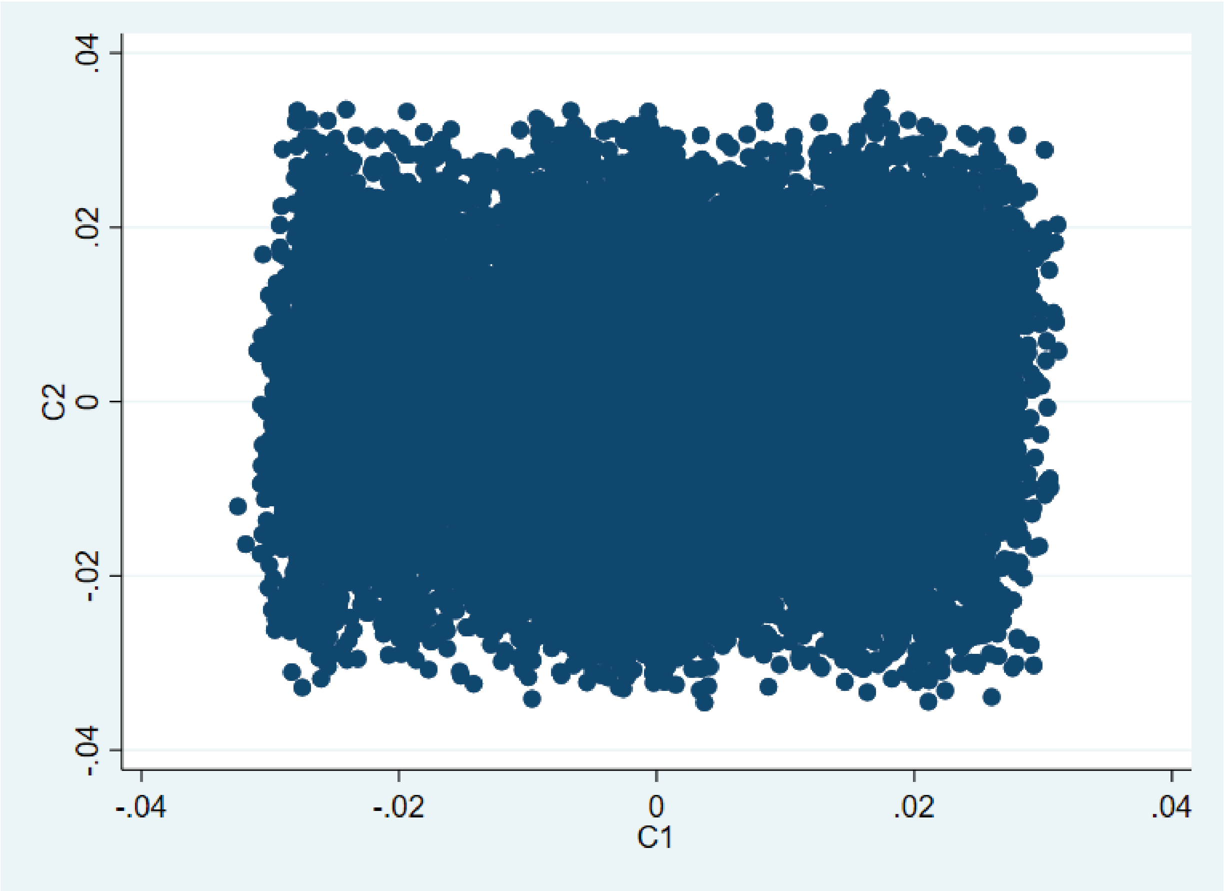

Supplement: Supplementary file 1 — Supplementary Information. [file 41598_2020_79964_MOESM1_ESM.pdf]
